# Sites for Dynamic Protein-Carbohydrate Interactions of *O*- and *C*-Linked Mannosides on the *E. coli* FimH Adhesin

**DOI:** 10.3390/molecules22071101

**Published:** 2017-07-03

**Authors:** Mohamed Touaibia, Eva-Maria Krammer, Tze C. Shiao, Nao Yamakawa, Qingan Wang, Anja Glinschert, Alex Papadopoulos, Leila Mousavifar, Emmanuel Maes, Stefan Oscarson, Gerard Vergoten, Marc F. Lensink, René Roy, Julie Bouckaert

**Affiliations:** 1 Pharmaqam, Department of Chemistry, Université du Québec à Montréal, P. O. Box 8888, Succ. Centre-ville, Montréal, QC H3C 3P8, Canada; mohamed.touaibia@umoncton.ca (M.T.); chichi_shiao@hotmail.com (T.C.S.); wange6988@yahoo.fr (Q.W.); usc.alex24@gmail.com (A.P.); leilyanmousavifar@gmail.com (L.M.); 2 Department of Chemistry and Biochemistry, Université de Moncton, Moncton, NB E1A 3E9, Canada; 3 Unité de Glycobiologie Structurale et Fonctionnelle (UGSF), UMR8576 du CNRS, Université de Lille, F-59000 Lille, France; eva-maria.krammer@univ-lille1.fr (E.-M.K.); Nao.Yamakawa@univ-lille1.fr (N.Y.); emmanuel.maes@univ-lille1.fr (E.M.); Gerard.Vergoten@univ-lille1.fr (G.V.); marc.lensink@univ-lille1.fr (M.F.L.); 4 Center for Synthesis and Chemical Biology (CSCB), University College Dublin, Belfield, Dublin 4, Ireland; Anja.Glinschert@gmx.de (A.G.); stefan.oscarson@ucd.ie (S.O.)

**Keywords:** *C*-glycosidic linkage, *ortho*-biphenyl mannose, FimH, anti-adhesive, uropathogenic *E. coli*, clamp loop, dynamic binding

## Abstract

Antagonists of the *Escherichia coli* type-1 fimbrial adhesin FimH are recognized as attractive alternatives for antibiotic therapies and prophylaxes against acute and recurrent bacterial infections. In this study α-d-mannopyranosides *O*- or *C*-linked with an alkyl, alkene, alkyne, thioalkyl, amide, or sulfonamide were investigated to fit a hydrophobic substituent with up to two aryl groups within the tyrosine gate emerging from the mannose-binding pocket of FimH. The results were summarized into a set of structure-activity relationships to be used in FimH-targeted inhibitor design: alkene linkers gave an improved affinity and inhibitory potential, because of their relative flexibility combined with a favourable interaction with isoleucine-52 located in the middle of the tyrosine gate. Of particular interest is a *C*-linked mannoside, alkene-linked to an *ortho-*substituted biphenyl that has an affinity similar to its *O*-mannosidic analog but superior to its *para-*substituted analog. Docking of its high-resolution NMR solution structure to the FimH adhesin indicated that its ultimate, *ortho*-placed phenyl ring is able to interact with isoleucine-13, located in the clamp loop that undergoes conformational changes under shear force exerted on the bacteria. Molecular dynamics simulations confirmed that a subpopulation of the *C*-mannoside conformers is able to interact in this secondary binding site of FimH.

## 1. Introduction

Pathogenic *E. coli* adheres via the FimH adhesin at the tip of their type-1 fimbriae to mannosylated glycan receptors on epithelial linings [1,2]. The use of mannose-based anti-adhesives for the selective inhibition of type 1-pilus mediated bacterial adhesion [3,4,5,6,7,8,9,10,11,12,13,14,15,16,17,18,19,20,21,22,23,24] has attracted great interest for the non-antibiotic treatment of urinary tract [1,25] and intestinal [2,17,26] infections caused by pathogenic *Escherichia coli*. These bacteria can express an arsenal of multiple adhesins, lectins with a variable immunoglobulin fold, for their attachment to and colonization of host cells.

**Figure 1:**
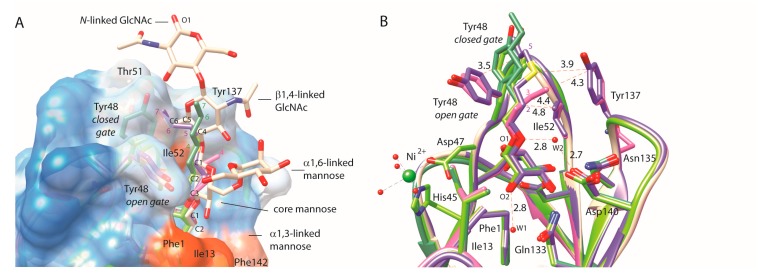
Crystal structures of *O*-alkylated α-d-mannopyranosides as FimH antagonists, with aglycones heptyl **5** (green) in the *closed* tyrosine gate (PDB entry 4buq [13]), thioalkyl **37** (prussian blue, sulfur atom in yellow) in the *open* gate (PDB entry 4avh [9]) and butyl **4** in the *closed* gate (apple green, PDB entry 1uwf [4]) and in the *open* gate (magenta, PDB entrz 1tr7 [4]). (**A**) The complexes have been superimposed onto the FimH lectin domain complex with oligomannose-3 (almond white), bound in the *open* tyrosine gate (PDB entry 2vco [5]) and displayed on a hydrophobicity-colored surface (dodger blue for the most hydrophilic, to white, to orange red for the most hydrophobic). Atom numbering in the aglycons starts from the first carbon atom following the glycosidic *O*-linkage; (**B**) The same compounds are bound as in (**A**), with potential van der Waals contacts and hydrogen bonds shown in orange, dashed lines labelled with the inter-atomic distances in Å. W1 and W2 denote structural water molecules important for the stabilization of the mannose that plugs into the mannose-binding pocket.

**Figure 2:**
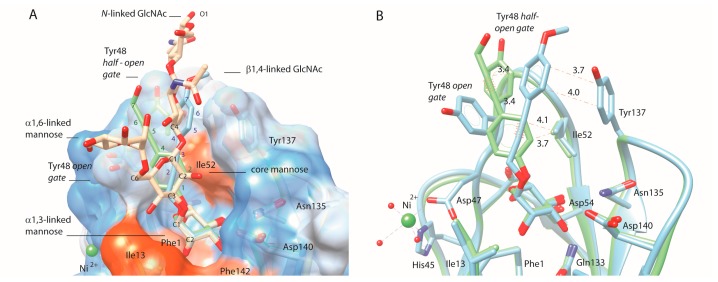
Compounds that only differ in its alkyne-phenyl order as bound in the FimH crystal structures: **56** (blue, PDB entry 4av0) and its analog **61** (green, PDB entry 4auy). A wire wrapped around the bond between carbon atoms at position two and three, or five and six, respectively in compound **56** or **61**, indicates the location of the alkyne. (**A**) Illustration of the overlay of the two ligand aglycons with the C1-C2-C3 carbon atoms of the core mannose of *N*-linked glycans, displayed on a hydrophobicity-colored surface presentation of the FimH lectin (dodger blue for the most hydrophilic, to white, to orange red for the most hydrophobic). The atom numbering in the aglycons starts from the first carbon following the *O*-glycosidic linkage; (**B**) Van der Waals contacts (≤4.5 Å, dashed lines in the same color as the structure) are indicating contacts made by the **56**-ligand (blue) in the *open* tyrosine gate and by the **61**-ligand (green) with the half-open conformations of Tyr48.

The α-d-mannopyranoside-binding lectin FimH recognizes, among other glycosylated receptors, the multiantennary glycoprotein uroplakin Ia and attaches the bacteria to these receptors leading to *cystitis* (bladder infections). The receptor-binding site of the FimH consists of a highly specific mannose-binding pocket, with a tyrosine gate (Tyr48, Ile52 and Tyr137) at one extension and a hydrophobic ridge (Ile13, Phe1, Phe142) at the other edge lining its entry [24,25] (Figure 1 and Figure 2).

The receptor-binding pockets in glycan-binding proteins often include tyrosine (and tryptophane) residues with their aromatic side chains stacking against the apolar face of the pyranoside ring [27]. Such strong CH-π stacking interactions also clamp oligomannoside-3 in the tyrosine gate of FimH [5]. The *open* tyrosine gate conformation (Tyr48 side chain torsion angles near χ1 = −57°, χ2 = 83°) allows the natural glycan receptor with highest affinity, oligomannoside-3, to pass in between the tyrosine gate residues. The *half-open* conformation, reflecting a major change in the χ1 torsion angle of the Tyr48 side chain (near χ1 = −164°, χ2 = −97°), prevents the ligands from becoming sandwiched between the tyrosine side chains [9]. Subsequent rotation around the χ2 torsion angle of the Tyr48 side chain (near χ1 = −160°, χ2 = 10.8°) closes the tyrosine gate. Thus far, the *half-open* and *closed* gate conformers have only been observed in complexes with synthetic anti-adhesive molecules.

Due to its unique design, the FimH binding site accommodates rather well α-d-mannopyranosides substituted with apolar aglycons, making these compounds very potent FimH inhibitors. Mannopyranosides α-substituted with heptyl [4,13,14,24,28] or with thiazolylamine [12,21,22,29] have been described as the most potent *O*-linked FimH ligands. However, *O*-linked compounds are possibly susceptible to degradation by glycosidases. Low bioavailability and half-life of *O*-mannosides can be attributed to the metabolic instability of the *O*-glycosidic bond [11,18]) and the hydrolysis by mannosidases [30,31]. Substitution of the *O*-glycosidic linkage by a *C*-glycosidic linkage was recently shown to lend important advantages in the activity of mannose-based anti-adhesives on the elimination of *E. coli* of intestinal cells [29] as well as for the treatment of urinary tract infections [18,21]. *para*-Biphenyl derivatives *O*- and *C*-linked to α-d-mannopyranosides are under drug development [6,7,18,32]. Based on the accumulated evidence that FimH interacts with hydrophobic substituents on α-d-mannopyranosides using its tyrosine gate [4,5,7,9,12,13,14,15,19,28], we set out to determine specificity- and selectivity-enhancing structural parameters for the optimization of the binding of drug-like anti-adhesive mannosides carrying either *O*- or *C*-glycosidic linkages to FimH.

In our search for challenging targets that might surpass the potency of known *O*-linked mannopyranosides, we prepared α-d-mannopyranoside derivatives with hydrophobic *O*- and *C*-linked substituents (for an exhaustive list and the synthetic details, see Supplementary Information) and sought for structure-activity relationships (SARs) explaining their binding to FimH. Amide, sulfonamide, thioalkyl or aliphatic alkyl, alkenyl or alkynyl functionalities have been introduced as spacers between the *O-* or *C*-anomeric atom of the glycosidic bond and the aglycons, in order to enable interactions in the extended high-mannose glycan-binding pocket of FimH [5]. The solution affinities of 55 mannosidic compounds with the *E. coli* FimH adhesin were measured using surface plasmon resonance (SPR) detection and, where available, compared with solution affinities obtained in isothermal titration calorimetry (ITC) measurements [9]. The mannosidic ligands with the most promising affinities were further tested for their potential to inhibit bacterial adhesion using a cellular assay, by inhibition of haemagglutination (HAI). The structural basis of the affinities was examined by screening for interactions made between ligands and the FimH binding site using available crystal structures and molecular simulation data of the complexes. As part of the SARs, we present the high-resolution NMR solution structure of a high-affinity *C*-linked *ortho*-biphenyl mannose derivative and its proposed binding modes to FimH, using induced-fit docking and molecular dynamics simulations. The results of this study were used to construct a general view of the dynamic binding modes of mannose-based anti-adhesives to the *E. coli* FimH. We hope this SARs study provides a valuable vocabulary for the rational drug design of anti-adhesive molecules against uropathogenic *E. coli* infections.

## 2. Results

### 2.1. Alkane Aglycons, the Nature and Dynamics of Their Interactions

A first series of compounds comprised alkylated α-d-mannopyranosides as FimH binders. Solution affinities of different mannosides for the FimH lectin domain (residues 1–158) have been measured using SPR by competition of the soluble compounds with an immobilized receptor. In initial measurements, the immobilized receptor was a Fab fragment derived from a monoclonal antibody (IC10 (MedImmune, Gaithersburg, MD, USA), K*_d_* = 220 nM) used previously to identify the highest-affinity natural epitope for FimH (K*_d_* = 20 nM for oligomannoside-3 (Figure S19) [33]. The measurements of the highest-affinity compounds were further refined by repeating the SPR experiments using 8-aminooctyl α-d-mannopyranoside (**5b**) coupled onto the carboxymethylated dextran of the sensor chip (CM5, Biacore3000, GE Healthcare Life Sciences, Marlborough, MA, USA) via usual amide formation [34]. The steady-state affinity of **5b** (Ss in Table 1) is determined by direct binding of FimH to the mannose derivative coupled to the sensor chip and is almost identical to its solution affinity of octyl α-d-mannopyranoside (**5a**, K*_d_* = 21 nM, Figure S19), indicating that **5b** can be bound by the FimH lectin without steric hindrance from the sensor chip surface, thus providing a simpler and effective bioanalysis.

Compounds **35**–**39** including a sulfur atom in the alkyl chain of *O*-mannosides were found to be weaker binders than *O*-mannopyranosides with plain alkyl chains (compounds **4**–**5**), for example **37** (hepta) and **38** (hexa) vs. heptyl (compound **5**) and hexyl (K*_d_* = 10 nM) [4] α-d-mannopyranosides (Table 1). Interestingly, ITC and HAI measurements confirmed that heptyl α-d-mannopyranoside **5** exhibited inhibition potency that was ~4-fold higher than its 4-thio analog **37**. This observation provided validation for the experimental methods in this affinity range. A property shared by alkane-substituted *O*-(**5**, **37**) and *C*-linked (**107**) mannopyranosides is the appearance of an optimum affinity for those mannosides with a chain length of seven atoms beyond the glycosidic linkage (Table 1). This length coincides with the optimal binding interactions of the natural oligomannose-3 receptor in the tyrosine gate of FimH (Figure 1).

The overlay of mannosides bound in the crystal structures shows how far the compound structures reach beyond the α1,3-linked mannose bound in the mannose-binding pocket of FimH and how they can mimic contacts of the oligomannoside-3 *N*-glycan receptor with the FimH protein (Figure 1). The alkyl substituents follow the hydrophobic “B”-side (C1-C2-C3) of the core mannose (Figure 1A). Together with the C2-C3 bond of the α1,3-linked mannose, carbon atoms two and three of the alkyl interact with the Ile52 side chain (Figure 1B). At atomic position four, the alkyl chains diverge for making CH-π interactions with either one of the tyrosine side chains. In the *open* gate, the atom at position four prefers Tyr137, as seen for the end-standing methyl group of **4** (magenta) and the sulfur atom of **37** (prussian blue). In the *closed* tyrosine gate, the interactions are predominant with the extended conformation of the Tyr48 side chain for the butyl **4** (apple green) and the heptyl **5** (green) α-d-mannopyranosides. In summary, ligands in the *open* gate interact more intensely with Ile52 and Tyr137, whereas residues in the *closed* gate interact more strongly with Ile52 and Tyr48. The first two or three atoms beyond the glycosidic oxygen interact with the Ile52 side chain.

In the crystal structure of FimH in complex with **37 [9]**, the electron-rich sulfur atom is polarized in its interaction towards the Tyr137 side chain. It contributes to interactions in the hydrophobic gate similar to the Manβ1,4GlcNAc glycosidic linkage in oligomannoside-3, namely four atoms beyond the glycosidic linkage (yellow, dashed line in Figure 1B). Carbon atoms at position five, six and seven after the glycosidic linkage of **37** (prussian blue) follow the C5-C6-O6 bond of the trimannose core-linked GlcNAc and stack with Tyr48 of the *open* tyrosine gate (Figure 1A). The *O*-linked alkyl mannosides presented thus mimic relatively well the natural oligomannose-3 receptor binding with the FimH lectin, also when they contain the sulfur at anomeric position four beyond the glycosidic linkage. For *C*-linked mannopyranosides **106**–**110** (Table 1) in contrast, the sulfur atom in the corresponding is positioned one atom earlier and would fall shortly near Ile52 (in the *open* gate conformer) or Tyr48 (in the *closed* gate) (Figure 1B). Its repulsion might have given rise to slightly lower affinities. This was indicative that a heteroatom’s position in the aglycon and thus in the tyrosine gate determines its translating into affinity. Bulkier and electron-rich atoms appear better positioned between positions four or six beyond the glycosidic linkage, but not on position two or three where they might undergo repulsion by either Ile52 or Tyr48. The following sections, detailing FimH affinities for compounds bearing alkane, alkene and alkyne linkers between the mannose and aromatic substituents, provide further evidence for this.

### 2.2. An Alkene, But Not an Alkyne, Linker Increases Affinity by Interaction with Ile52

The *O*-linked mannopyranosides **22**–**25** carrying an alkenyl aglycon were found to be better FimH inhibitors than their hydrogenated alkane analogs **26**–**29** (Table 2). K*_d_* values below 5 nM for **23**–**25** make these compounds at least equipotent to the reference FimH antagonist heptyl α-d-mannopyranoside **5**. It appears that the apolar interactions created between the double bond of the *O*-linked mannosides **22**–**25** and FimH contribute more favorably to the affinity than those created by a single bond. The double bond is made between atom positions two and three beyond the glycosidic linkage. These carbon atoms are most likely to make hydrophobic contacts with Ile52, a residue important in oligomannose-3 binding (Figure 1). Next we examined whether the affinity could be further improved by the introduction of a more rigid alkynyl linker between the aromatic moiety and the anomeric oxygen of mannose (Table 2 and Table 3).

Among alkyne, alkene and alkane-substituted *O*-mannopyranosides, the alkene with its double bond appears most suitable for inhibition of FimH (Table 2). Nevertheless, the HAI data indicated that alkanes are equal (compound **27**) or even 2-fold better (compound **29**) in inhibitory potency than the alkenes (compounds **23** and **25**, respectively) (Table 2 and Table S2). Alkyls are slightly less soluble than alkenyls and it is presumable that the inhibition potential by **27** and **29** has been slightly hampered by the lack of DMSO in the SPR experiment. 

Although the enhanced affinity of the alkenes over the alkanes may be negligible, it is directed by specific recognition events at post-glycosidic linkage atomic positions two to three with Ile52 and Tyr48 of the tyrosine gate (Figure 1B and Figure 2B), rather than singly by a hydrophobic effect. The alkynyl inhibitors **50**–**53** were much less active than the alkenyl linked mannopyranosides **22**–**25** and the alkyl-linked compounds **26**–**29**. In contrast to the alkenyl mannopyranosides, the rigidity of the *sp*^1^ hybridization of the alkynyl linker is likely to impede significantly the positioning of the aglycon in its most preferred binding conformation. This rigidity effect dominates over possible positive dispersion interactions between the alkynyl linker and the hydrophobic gate.

The substitution of the phenyl group of the alkyne derivative **50** (K*_d_* = 168 nM) by either *para* (**51**) or *ortho* (**52**) biphenyl showed improved potency (Table 2). The introduction of naphthalene (**53**, K*_d_* = 120 nM), 2-thiophene (**54**, K*_d_* = 113 nM), 3-pyridine (**55**, K*_d_* = 83 nM) and a *para*-methoxyphenyl group (**56**, K*_d_* = 53 nM) also progressively improved the affinity (Table 3). A nitro group at the *para* position of its phenyl group (**57**, K*_d_* = 816 nM) significantly diminished the affinity compared with **56**, most probably because this electron-withdrawing group decreases its stacking potential with Tyr137 (described in [13]). Compounds **56**, **61** and **62** each contain an aromatic ring and a propargyl group in their apolar tail, but with inverted order of spatial disposition for these functional groups. Among these three compounds, the affinity is highest for **61**. In the co-crystal structure of **61** bound to the FimH lectin domain (PDB entry 4auj), the ligand sterically clashes with Tyr48 in its *open* gate conformation: the bulky phenyl group of **61**, juxtaposed to its glycosidic linkage, dislocates the Tyr48 side chain and forces it into dynamic alternative conformations such as the *half*-*open* tyrosine gate conformation (Figure 2) [9,13].

The trend of increasing affinity of FimH for compounds **61** > **62** > **56** obtained in SPR measurements is confirmed in the HAI assay and by affinities previously obtained in ITC measurements [9] (Table 3). In order to correlate the affinity of **56** and **61** with their binding mode to FimH, these compounds had been soaked into crystals of ligand-free FimH in the trigonal space group (PDB entry 4auu) [9]. These crystals have the important advantage that no crystal packing contacts are present at the level of the tyrosine gate. Therefore, a compound that binds into the tyrosine gate of such FimH crystals experiences similar degrees of freedom as in solution. The thus obtained crystal structures of FimH show that the two aglycons indeed stack very differently in the tyrosine gate (Figure 2). Compound **61** also shows more potential for making close hydrophobic contacts (Figure 2A) when compared with **56** (Figure 2B), supported by its higher affinity for FimH (Table 3). The terminal hydroxyl group of **61** is in contact with the solvent, where it is coordinated in a hydrogen-bonding network via a water molecule to the hydroxyl groups of Tyr48 and Thr51. The observed hydrogen network cannot be formed by compound **62** that bears a methoxy instead of a hydroxyl group. This might be the structural reason behind the 2–3 fold improved affinity of compound **61** over **62** (Table 3).

### 2.3. Sulfonamide and Amide Spacers: Worse Fit and Bulky Compensation

Next, mannopyranosides with sulfonamide- and amide-based spacers were tested and the nature of the glycosidic aglycones was changed from *O*- to *C*-linkages. Several interesting SAR trends could be derived from this series (Table 4). The *O*-linked sulfonamides (**70**–**75**), just like the alkynyl in ligands **41** and **50**–**57** (Table 3), introduced stiffening just beyond the *O*-glycosidic linkage. This prohibits an arbitrary positioning of the ligand in the tyrosine gate according to best complementarity and may explain why the ligands profit from increased interaction possibilities through an increase in size and polarizability (Table 4). For example, there was a significantly improved potency for the tri-isopropyl-substituted phenyl derivative **74** (K*_d_* = 37 nM) that has its phenyl ring extended with bulky hydrophobic groups. Replacing the phenyl with a naphthalene group did not increase the affinity (**75**, K*_d_* = 148 nM), and the insertion of the electron-withdrawing nitro group had the opposite effect (**71**, K*_d_* = 408 nM) (Table 4).

We discovered that the FimH affinity towards *C*-linked mannosides **91**–**96** could be improved by the insertion of a flexible alkyl spacer between the phenyl ring and the amide function and that the length of this alkyl spacer was important. Indeed, while mannoside **92** (K*_d_* = 397 nM) that contains a methylene spacer was still as potent as the aromatic amide **91** (K*_d_* = 372 nM), the FimH inhibitory capacity rose 4-fold for **93** (K*_d_* = 105 nM) carrying a propyl spacer thus placing the aromatic moiety at the sixth position from the *C*-glycosidic linkage. The introduction of an ethyl linker had no enhancer effect (**96**, K*_d_* = 970 nM).

### 2.4. C-Mannopyranosides versus O-Mannopyranosides

In accordance with recent studies reporting that the FimH binding affinity and pharmacological characteristics of alkyl- and aryl α-d-mannopyranosides could be further improved by the insertion of extra substituents [9,16,17,19,20,21,22,23,24,25,26,29] substituting the *para* and *ortho* positions of the phenyl ring with an additional phenyl ring or inserting a naphthalene group most frequently surpassed the binding affinity of the unsubstituted phenyl derivative (Table 5). The inhibition potential of **116** and **117** exceeds the one of their parent **115** some 4- and 26-fold, respectively, indicating additional interactions of the extra phenyl substituent in the tyrosine gate.

The affinities for the *C*-linked compounds bearing amide linkers (Table 4) can be directly compared with analogs with alkene or alkane linkers (Table 5), because of their identical R-group in the aglycons. For example, the alkene-linked compound with a phenyl group, **115** (K*_d_* = 182 nM), is 2-fold more active than its amide-linked analog **91** (K*_d_* = 372 nM). We examined whether an alkane instead of an alkenyl spacer would increase the potency further. Three of the five aryl *C*-mannopyranosides (**119**, **120**, **122**) with alkyl linkers have better affinities than their corresponding alkenyl parents and HAI data confirmed this trend (see also Table S2).

For these compounds, the more limited conformational freedom of the double bond in the alkenyl mannopyranosides, relative to the single bond in the alkyl mannosides, may actually hinder the aryl moiety from occupying its preferred position in the hydrophobic groove of the FimH binding pocket. The crystal structures of FimH revealed that carbon atoms at positions two and three beyond the glycosidic atom make van der Waals contacts with Ile52, explaining why the double-bond character of this position would be beneficial (Figure 1).

The alkenyl occurs one atomic position earlier in our *C*-linked compounds, thus possibly generating less van der Waals contacts with Ile52, or pulling bulky aromatic groups deeper inside the tyrosine gate. Therefore, the affinities of alkenylated *C*-linked mannopyranosides for FimH are generally a bit lower than for their *O*-linked analogs, for example the affinity of the alkenyl phenyl (**115**, Table 5 vs. alkyl phenyl (**22**, Table 2) is indicative of the latter.

The **117**/**121** alkenyl/alkyl pair is an exception to this trend. This may be due to the different and specific interactions of **117** with FimH. The *ortho*-substituted **117** (Table 5) demonstrated an in vitro affinity similar to its *O*-analog **24** (Table 2). We decided to further explore the structural basis for its successful design using NMR spectroscopy, crystal structures, rigid and induced-fit docking approaches and molecular dynamics (MD) simulations.

### 2.5. Ortho-Biphenyl C-Mannopyranoside ***117***

**Figure 3:**
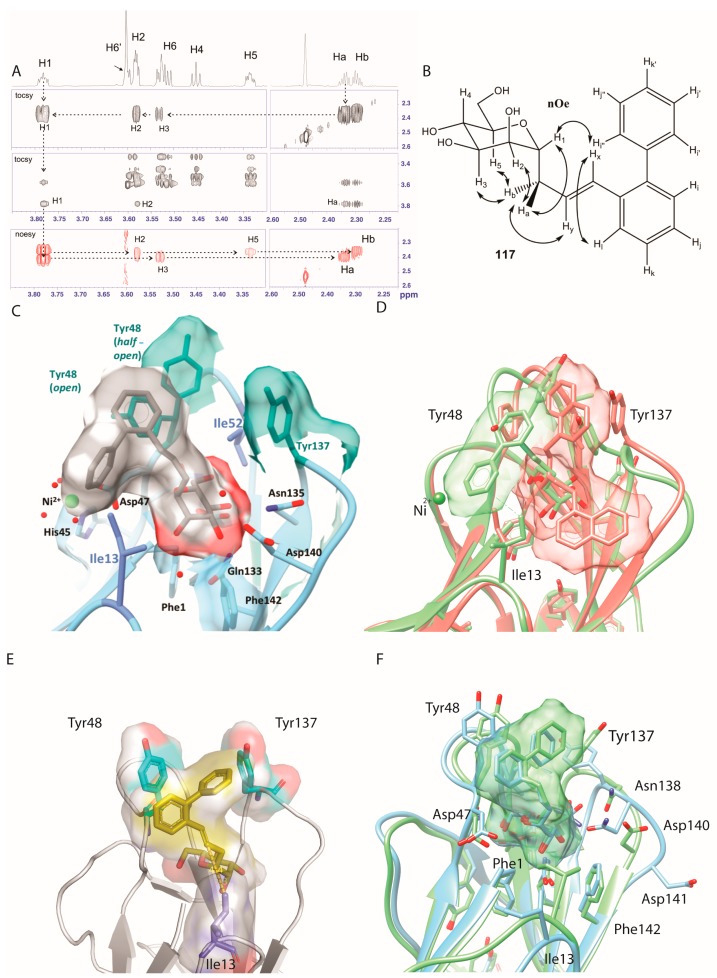
(**A**) Proton assignments based on NOESY and TOCSY, for compound **117** *C*-linked mannopyranoside. Quaternary carbons were attributed with the help of HMBC experiments; (**B**) Conformation of **117** at 293 K in DMSO-*d*_6_; (**C**) The NMR-derived solution conformation of **117** superposed in FimH of PDB entry 4auy; (**D**) Superposition of the simulated minor conformer of **117** (green), as extracted from the MD simulation without internal structural waters (minor, 11%), with PDB entry 5abz (salmon) presenting two alternative binding modes of a naphthyl mannoside; (**E**) Best solution for the binding of **117** from induced fit docking and (**F**) comparison with the major binding modes as simulated using MD, for 56% and 61% of the time in the form with the internal structural waters included (green) or not included (blue), respectively.

The three-dimensional structure of **117** was determined using high-resolution (900 MHz) NMR in DMSO-d_6_ solution. This *C*-linked analog is in the usual ^4^C_1_ conformation as judged by the total lack of nOe between H1 and H4/H6, together with strong nOe between the germinal aglycon Ha,b protons with the axial H3 and H5 protons (Figure 3A,B, Table S1, Figures S14–16). The NMR-derived structure of *C*-linked *ortho*-biphenyl ligand **117** was superposed onto the *O*-mannopyranoside of **61** (PDB entry 4auy) bound into the mannose-binding pocket of FimH that features both the *open* and *half-open* tyrosine gate conformers (Figure 3C) [9]. This revealed an as yet unexplored secondary mode of binding onto FimH, where its first phenyl ring interacts with Tyr48 and its second phenyl ring in the *ortho*-position docks near the isoleucine 13. Ile13 is located at the lower hydrophobic ridge of the mannoside-binding pocket of FimH, also called the clamp loop because it undergoes major conformational changes when FimH forms high-affinity catch bonds with mannosides [28]. Interestingly, this binding position was not retrieved as a low energy binding pose in different docking trials in which the binding site residues were either fully flexible or in which the tyrosine gate was held *open*, *half-open* or *closed*. Fully flexible docking always led to low-energy binding poses in a *half-open* or almost *closed* tyrosine gate, implying that Tyr48 makes an aromatic stacking interaction with the first phenyl ring of **117** (Figure 3E). The observed discrepancy between the docking and the NMR results led us to perform MD simulations.

**Figure 4:**
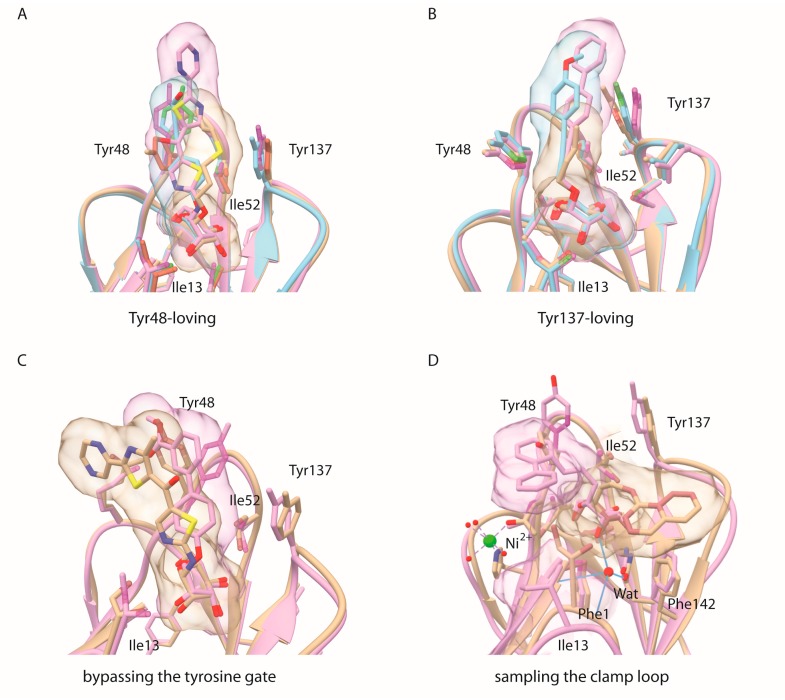
Different modes of binding to FimH. The way the carbohydrate-based inhibitors interact with the FimH lectin domain can be classified in major (**A**,**B**) and two minor (**C**,**D**) binding modes (**A**) Tyr48-loving: PDB entries 4avh (beige), 4auy (blue) and 5mts (fuchsia); (**B**) Tyr137-loving: 4av5 (fuchsia), 4av0 (blue), 1tr7 (beige); (**C**) bypassing the tyrosine gate: the ligand *para*-biphenyl α-d-mannose (PBD entry 3mcy, fuchsia) and a high-scoring docking pose of the ligand in PDB entry 5mts (beige), that both stack on the other side of Tyr48, outside the tyrosine gate; (**D**) sampling of aromatic substituents *C*-linked to mannose near Ile13 of the clamp loop, that traps the internal water molecule hydrogen bonding to the axial O2 hydroxyl of mannose: PDB entry 5abz (beige) and a MD cluster (11% of the total, when the internal structural water molecule was absent at the start of the simulations) of the *ortho*-biphenyl α-d-mannose **117**.

The best-scoring pose from the induced fit docking (Figure 3E), as well as the best-scoring poses from rigid dockings performed on *open*, *half-open* and *closed* tyrosine gate conformers, were subjected to MD simulations. Independent of the initial binding pose, the mannopyranoside of **117** always remained positioned in the same position and orientation in the MD trajectories. The non-mannosidic aglycon part was dynamic and featured four binding modes (Figure S21). Interestingly, the *closed*/Tyr48 conformer was very similar to what is observed for the best-scoring docking solution using induced-fit docking (Figure 3E) and the NMR-derived solution structure of **117** turned out to simulate well the *closed*/Ile13 binding conformer (compare Figure 3C with Figure 3D). However, visual inspection clearly pointed out that the *closed*/Ile13 ligand conformer was minor in presence and being visited only glancingly throughout the simulations. A possible explanation could be that the NMR solution structure of **117** was determined in DMSO and not in water. A simulation performed without the structural waters clearly highlighted that as long as these structural water were absent, the ligand was close to Ile13 (Figure 3D and Figure 4D) and that only after the water (W1, Figure 1B) was able to penetrate into the mannose-binding pocket, the ligand aglycon part orients itself towards the tyrosine gate (Figure 3F, movie provided in Supporting Information).

## 3. Discussion

Mammals possess many different types of α-d-mannopyranoside-binding lectins and thus target selectivity needs to be cautiously addressed [35]. Exposed at the edge of the type-1 pilus on the bacterial envelope, the FimH adhesin is maintained in a low-affinity conformation by interactions of the lectin domain with the pilin domain of FimH [36]. These quaternary interactions are disrupted when the bacteria adhered to a mannosylated surface and experience shear stress and consequently the FimH mannose-binding lectin domain is extended and forms catch bonds with the mannosides [37]. Despite that the crystal structures of the isolated lectin domain always contain the high-affinity FimH conformation, the binding site of FimH remains dynamic to enable the accommodate of a plurality of mannosylated receptors [24]. Tyr48 and Tyr137, together with Ile52, form the so-called tyrosine gate [4]. Their side chains are entropic adaptors to ligand structural changes and can adopt multiple conformations depending on ligand structure [9]. Indeed, several recent crystal structures of FimH complexes show the binding of hydrophobic aglycons substituents that fit poorly in the tyrosine gate and remain highly dynamic. Sometimes a high affinity can be acquired through such a dynamic binding, for example for compound **61**, where the interaction energy is mainly driven by dispersion [13]. In one of the two FimH molecules of the asymmetric unit of a crystal structure of the lectin domain in complex with a *C*-linked naphtyl mannose analog (PDB entry 5abz) [38], at least two alternative aglycon conformers were observed: a first one was bound between the two tyrosines in the *open* gate, whereas a second one hovered over the hydrophobic ridge above Phe142 (Figure 3D and Figure 4D). The same binding mode was found in docking experiments using bivalent mannosidic inhibitors, posing one arm in the *open* tyrosine gate and one arm above the hydrophobic ridge [39].

Derivatives of α-d-mannopyranoside with *O*- and *C*-glycosidic linkages bind the FimH adhesin, a mannose-specific lectin located at the tip of type 1 pili of pathogenic *E. coli*, and thereby inhibit bacterial adhesion. The structural basis of the affinities with the interactions made between ligands and the FimH binding site was examined using crystal structures of the complexes, which allows the construction of an overview of the dynamic binding modes of mannose-based anti-adhesives to the *E. coli* FimH adhesin. Alkyl and aryl derivatives of α-d-mannopyranoside with *O*- and *C*-glycosidic linkages can establish stacking interactions with the hydrophobic residues surrounding the mannose-binding pocket of the FimH adhesin of type 1-piliated uropathogenic *E. coli*. In earlier work, a *para*-biphenyl α-d-mannopyranoside with a short, flexible *O*-linkage, similar to the linker in compound **61**, was found to bypass the tyrosine gate and establish an aromatic stacking with Tyr48 on the outside of the tyrosine gate (PDB entry 3mcy, Figure 4C) [7]. With the structural studies of the *ortho*-biphenyl substituted *C*-linked mannopyranoside 117 (Figure 4D), more evidence is brought forward that the hydrophobic ridge exposing Ile13, Phe1 and Phe142 may also play an role as a secondary binding site in dynamic interactions of the FimH lectin with oligomannosylated receptors.

Only *C*-linked mannospyranosides derivatized with a *para*-substituted biphenyl have been further explored so far, both for their SARs [22,38,40] and their pharmacological characteristics [21,22,23,24]. Synthetic *C*-glycosides most often display a reduced activity compared to their *O*-linked parents [31]. However, *C*-glycosides are of higher stability, because they are not metabolized by the host enzymes [18,29,30]. Earlier comparisons of the bioactivities of *C*-glycosides with those of their *O*-glycosylated parents have uncovered several candidates with diminished or similar activity [8,9,18,29,41], but none exhibiting a significant improvement. Modification of the *O-*linked α-anomer by a *C*-linkage leads to a loss in polar character and thus hydrogen bonding capacity [29,38]. In the synthetic work presented here, it also implies a pharmacophore position shifting. In our studies, the affinities of the *C*-linked mannopyranosidic derivatives turned out to be relatively similar to their *O*-analogs. The *ortho*-biphenyl substituted *C*-linked mannopyranoside **117** constitutes a potent *C*-linked mannopyranoside derivative. Remarkably, the high-resolution NMR solution structure of **117** can be superposed in an active binding mode to a secondary site (including Ile13) that agrees with a minor binding mode from our MD simulations. The conformer adopted by the hydrophobic compound **117** when solubilised in DMSO-d_6_ can thus right away occupy an unusual hydrophobic patch on FimH guided by the Tyr48 and Ile13 residues in the hydrophobic surrounding of the FimH mannose-binding pocket. The *ortho*-biphenyl substituted *C*-linked mannopyranoside **117** has a higher affinity over the *para*-substituted analogue **116**. This can be explained by its particular binding modes: (1) the first phenyl ring stacks with the Tyr48 side chain; (2) the *ortho*-placed second phenyl ring interacts either with Tyr137 or with Ile13. Even so the simulations indicate that the position of **117** towards Ile13 is a minor conformation and is likely to occur in an early step of the binding event, before the waters can occupy their final place and being held in a hydrogen bonding network, this conformation is of particular interest as the change in conformation of the Ile13-containing loop is associated with shear-force enhanced bacterial adhesion.

Binding of anti-adhesives at the clamp loop holding Ile13 where large shear-force induced conformational changes occur, with the interplay of aglycon stacking with either the Ile13 or the Tyr48 side chain, is a mechanism as yet little explored in drug discovery of FimH antagonists of *E. coli* adhesion [42]. These novel structural insights, namely that *ortho*-biphenyl substituents on mannose can sample a binding interface on the FimH adhesin, notably one that is directly involved in the regulation of FimH affinity of pathogenic *E coli* under shear force, opens new perspectives for non-antibiotic therapies and anti-adhesive design [39,40,43].

## 4. Materials and Methods

### 4.1. Solution Affinity of Ligands for the FimH Adhesin Using SPR

#### 4.1.1. Steady State Affinity Measurement

The FimH lectin domain (Phe1-Thr158 of the *fimh* gene product of *E. coli* strain J96) was expressed and purified as described [5]. Affinity measurements using surface plasmon resonance (SPR) were performed on a Biacore 3000 as described earlier [4,33]. The equilibrium dissociation constant K*_d_* of FimH for the mannose derivatives was determined using a competition assay in solution. In this assay, the mannose derivatives compete with an immobilized Fab fragment from the monoclonal anti-FimH antibody 1C10 or with an immobilized 8-aminooctyl α-d-mannopyranoside (**5b**) for FimH binding, as described earlier [34]. The global kinetic constants k*_a_*, k*_d_* and R_max_ of the FimH-ligand interaction of binding were fitted using the BiaEval software. Subsequently, a constant concentration of FimH that was close to the K*_d_* of the FimH-ligand interaction was added to a concentration series of mannoside (2000 nM down to 1.95 nM) and flown over the Fab-immobilized or 8-aminooctyl α-d-mannopyranoside (**5b**) modified biosensor chip (Figure S18).

#### 4.1.2. Competition Method

The competition binding assay measures the amount of free FimH in solution using biosensor chips decorated with immobilised Fab fragments of a monoclonal antibody or with an immobilized 8-aminooctyl α-d-mannopyrannoside (**5b**). [FimH_nb_] = [FimH_0_] − [FimH-Man] (where [FimH_nb_] is the concentration of non-bound fraction of FimH, [FimH_0_] the intitial, total FimH concentration and [FimH-Man] the mannoside-bound FimH concentration). This amount of free FimH, measured by the SPR assay, is proportional to the dissociation constant for the FimH-mannoside binding. These concentrations of FimH unoccupied and able to bind to the ligand on the chip were then fitted by feeding the 1:1 Langmuir binding model, keeping the parameters k*_a_*, k*_d_* and R_max_ measured in the foregoing kinetic binding (to IC10 or **5b**) constant. For improved accuracy, two overlapping 2-fold dilution series of the mannoside derivatives were used to allow a smaller than 2-fold dilution concentration series and abundous data points. Using two parallel series 2-fold dilutions to create concentrations that were only 1.5-fold different, from 2000 nM down to 10 pM of mannoside derivatives, for most of the compounds, inhibition curves could be fitted to the equation (Figure S19), with some exceptions (Figure S20).

### 4.2. Haemagglutination Inhibition Assay

The SPR assay was not optimal for those mannosides with lower solubility because of incompatibility with the use of DMSO for the measurement of nanomolar affinities. Therefore we have cross-validated the compounds that displayed solubility issues and their analogues with results from another molecular assay (ITC) [9,13] and have performed one cellular assay, by haemagglutination inhibition (HAI). The inhibition of guinea pig red blood cell agglutination by the type-1 piliated uropathogenic *E. coli* strain UTI89 was evaluated in a 2-fold dilution series of the mannosides. UTI89 *E. coli* were grown statically overnight at 37 °C in LB (per liter of water: 10 g tryptone, 5 g yeast, 5 g NaCl). The bacterial pellet was washed by centrifugation and diluted in buffer (Na-Hepes 20 mM, NaCl 150 mM, pH 7.4) before determination of it haemagglutination titer. The mannosides (25 µL) were diluted in a 2-fold series in the same buffer as the bacteria, however 10% DMSO was maintained in the final volume (100 µL) of each well. The bacterial solution (25 µL) was added at a concentration of four times the bacterial haemagglutination titer. Finally, guinea pig red blood cells (50 µL), washed in buffer and diluted to 5%, were added and allowed to stand for 1 hour at 4 °C before read-out of the inhibition titer: the highest concentration of mannoside that can still inhibit the haemagglutination by bacteria. The titers for the mannosides are here expressed in µM.

### 4.3. Induced Fit Docking

Docking experiments were performed with the GOLD software (The Cambridge Crystallographic Data Centre, Cambridge, UK). The six heavy atoms of the mannose ring found in the coordinate file (PDB entry 4auy) were used as a scaffold in the active site. Two series of docking procedures were performed, with a rigid and a fully flexible ligand-binding site. The following residues in the binding site vicinity: Asp47, Tyr48, Ile52, Thr53, Asp54, Gln133, Thr134, Asn135, Asn136, and Tyr137 were also defined as flexible in the latter. For each ligand, 10 poses that were energetically reasonable were kept while searching for the correct binding mode of the ligand. The decision to keep a trial pose was based on a computed energy for the interaction of the ligand with receptor of that pose. The ChemPLP fitness scoring function is the default in GOLD version 5.2 used to rank poses. Additionally an empirical potential energy of interaction ∆E for the ranked complexes was evaluated using the simple expression ∆E(interaction) = E(complex) − (E(protein) + E(ligand) [44,45]. For that purpose, the Spectroscopic Emperical Potential Energy Function SPASIBA and the corresponding parameters are used [46,47]. Discovery Studio Visualizer 4.1 (Accelrys, San Diego, CA, USA) was used for viewing, Chimera version 1.8 (University of California, San Francisco, CA, USA) for figure preparation.

### 4.4. Molecular Dynamics Simulations

The best scoring complex of compound **117** and FimH from the induced fit docking with either the tyrosine gate helt *open* (system 1a), *closed* (system 2) or *half-open* (system 3) were used as the starting conformations for the MD simulations. The complex was solvated and the structural waters were added. The ionic concentration was set to 0.15 M NaCl. In accordance with propKa [48], the standard protonation state at pH 7 was used for all protonatable groups of FimH. The generated molecular system comprised about 45,000 atoms and had a size of about 66 × 90 × 70 Å. The CHARMM36 force field with CMAP corrections was used to describe protein, water, and ion atoms [49]. Missing force field parameters for compound **117** were initially generated with CgenFF [49] with standard parameters and afterwards adapted. The integrity of the compound was verified in a 50-ns long MD simulation of the compound alone in water using the adapted force field. Two independent simulations of the generated system were performed. In each of them, a three-step equilibration was applied: first, a 2.5-ns long equilibration of the water and ions molecules, second a 2.5 ns long equilibration in which only the protein backbone was fixed, and third unrestrained simulations was carried out for 2.5 ns. This was followed by a 30-ns long production run. In addition, a single simulation was performed using the same protocol as described above with the system 1a only without placing the structural waters (system 1b). All MD calculations were performed in the isothermal-isobaric ensemble at 300 K with the program NAMD2.9 [50] using periodic boundary conditions. Long-range electrostatic interactions were calculated using the particle-mesh Ewald method [51]. A smoothing function was applied to truncate short-range electrostatic interactions. The Verlet-I/r-RESPA multiple time-step propagator was used to integrate the equation of motions using a time step of 2 and 4 fs for short- and long-range forces, respectively [52]. All bonds involving hydrogen atoms were constrained using the Rattle algorithm [53]. The most abundant conformations of the ligand were determined by clustering one trajectory of the systems 1a and 1b using the G_CLUSTER tool from the MD suite GROMACS [54], based on the root-mean-square deviation matrix of the ligand.

## Figures and Tables

**Table 1:**
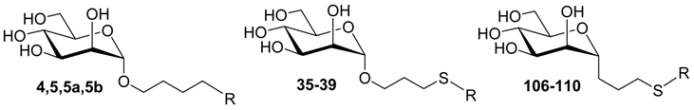
**Summary of SARs on alkane aglycons:** The solution affinity of the FimH lectin domain for and *O*- and *C*-linked alkanes and thioalkanes. Ss: steady state affinity for **5b**.

R	Cpd	K*_d_* SPR (nM) K*_d_* ITC (nM) HAI (µM)	R	Cpd	K*_d_* SPR (nM) K*_d_* ITC (nM) HAI (µM)	Cpd	K*_d_* SPR (nM) K*_d_* ITC (nM) HAI (µM)
H	**4**	151 [4] 156 ± 45 [9] 100	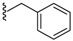	**35**	16 ± 2.4 -- --	**106**	25 ± 6.3 -- --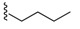	**5a**	21± 0.2 [4] -- --	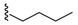	**36**	22 ± 2.7 -- --	**107**	22 ± 2.8 -- --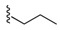	**5**	4.3 ± 0.3 [4] 7.3 ± 1.8 [9] 6.25	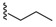	**37**	14 ± 5.0 59.5 ± 4.7 [9] 25	**108**	36 ± 9.0 -- --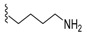	**5b**	18 ± 0.4 Ss 17 ± 0.4 --	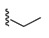	**38**	14 ± 7.6 -- --	**109**	78 ± 5.6 -- --
			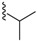	**39**	11 ± 6.1 -- --	**110**	32 ± 4.1 -- --

**Table 2:**
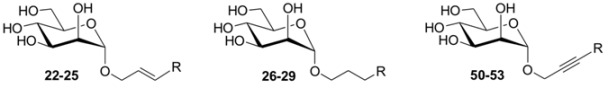
Affinities of FimH for *O*-linked alkene-(**22**–**25**), alkane-(**26**–**29**) and alkyne-(**50**–**53**) derivatized *O*-mannopyranosides, as measured in SPR and/or ITC experiments, and their minimal concentrations inhibiting haemagglutination (HAI).

R	Cpd	K*_d_* SPR (nM) K*_d_* ITC (nM) HAI (µM)	Cpd	K*_d_* SPR (nM) K*_d_* ITC nM) HAI (µM)	Cpd	K*_d_* SPR (nM) K*_d_* ITC (nM) HAI (µM)
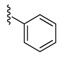	**22**	53 ± 7.5 -- --	**26**	59 ± 8.5 -- --	**50**	168 ± 15 -- --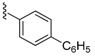	**23**	3.0 ± 1.1 -- 25	**27**	10 ± 0.2 1.5 ± 0.3 25	**51**	405 ± 24 61 ± 9.6 100
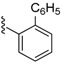	**24**	4.3 ± 0.8 -- --	**28**	13 ± 1.7 -- --	**52**	2250 ± 291 -- --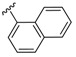	**25**	5.0 ± 1.4 71 ± 23 50	**29**	22 ± 3.0 -- 25	**53**	120 ± 18 -- 50

**Table 3:**
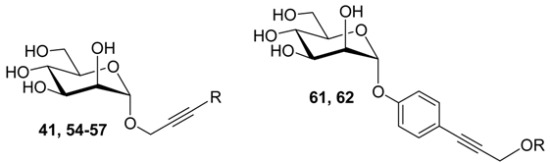
The affinity of the FimH lectin domain for alkyne *O*-mannopyranoside series **41**, **54**–**57** (**56**: PDB entry 4av0) and two compounds with an inverted aglycon structure compared to **56**:**61** (PDB entry 4auj) and **62**. * nf: SPR data could not be fitted, see also Figure S20.

Cpd	R	K*_d_* SPR (nM)	K*_d_* ITC (nM)	HAI (µM)
**41**	H	490 ± 55	--	--
**54**	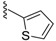	133 ± 31	--	--
**55**	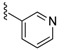	83 ± 5.6	105 ± 34	50
**56**	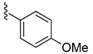	53 ± 2.3	104.6 ± 21.9	50
**57**	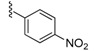	816 ± 105	--	--
**61**	H	36.9 ± 3.0	18.3 ± 5.9	12.5
**62**	Me	nf *	59.5 ± 2.3	25

**Table 4:**
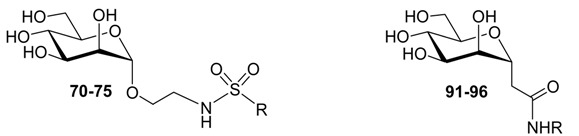
The affinities of FimH for *O*-linked mannopyranoside sulfamides **70**–**75** and their *C*-linked mannopyranoside amide analoges **91**, **92**, **93** and **94**, **95** and **96**, as measured using SPR detection.

Cpd	R	K*_d_* (nM)	Cpd	R	K*_d_* (nM)	Cpd	R	K*_d_* (nM)	Cpd	R	K*_d_* (nM)
**70**	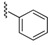	146 ± 26	**73**	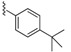	122 ± 11	**91**	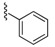	372 ± 26	**94**	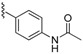	227 ± 54
**71**	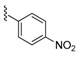	408 ± 52	**74**	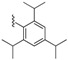	37 ± 5	**92**	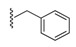	397 ± 83	**95**	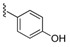	328 ± 63
**72**	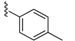	168 ± 64	**75**	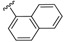	148 ± 18	**93**	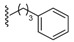	105 ± 44	**96**	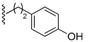	970 ± 382

**Table 5:**
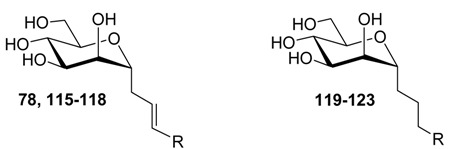
The affinity (using SPR) of FimH for *C*-linked mannopyranosides **78**, **115**–**118**, **119**–**123**. nf: no good fit could be obtained of the data to the Langmuir 1:1 binding model (Figure S20).

R	Cpd	K*_d_* (nM)	Cpd	K*_d_* (nM)
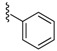	**115**	182 ± 25	**119**	49 ± 5.3
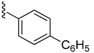	**116**	17 ± 3.5	**120**	nf
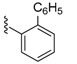	**117**	6.9 ± 5.7	**121**	123 ± 21
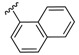	**118**	nf	**122**	nf
H	**78**	118 ± 20	**123**	266 ± 26

## Supplementary Materials

Supplementary materials are available online. For detailed descriptions and results on synthetic protocols of the compounds and their NMR structural analyses and the measurements of the potency of compounds using inhibition assays based on whole cell interactions (HAI) vs*.* based on molecular interactions either via competition (SPR) or direct (ITC), see SI.
