# Supplementary material for: Sites for Dynamic Protein-Carbohydrate Interactions of O- and C-Linked Mannosides on the E. coli FimH Adhesin
Source: Molecules. 2017 Jul 3;22(7):1101. doi: 10.3390/molecules22071101 (PMC6152123; doi:10.3390/molecules22071101)
Supplement: Supplementary file 1 [file molecules-22-01101-s001.zip › SI/Supporting Information Molecules.pdf]

Supporting Information

Sites for dynamic protein-carbohydrate interactions of *O*- and *C*-linked mannosides on the *E. coli* FimH adhesin

Mohamed Touaibia,[†,‡,*] Eva-Maria Krammer,[§,*] Tze C. Shiao,[†] Nao Yamakawa,[§] Qingan Wang,[†] Anja Glinschert,[⊥] Alex Papadopoulos,[†] Leila Mousavifar,[†] Emmanuel Maes,[§] Stefan Oscarson,[⊥] Gerard Vergoten,[§] Marc F. Lensink,[§] René Roy,[†,⌘] Julie Bouckaert,[§,⌘]

‡ M. Touaibia

Department of Chemistry and Biochemistry, Université de Moncton, Moncton, New Brunswick, Canada

§ E.-M. Krammer, N. Yamakawa, E. Maes, G. Vergoten, M. F. Lensink, J. Bouckaert

Univ. Lille, CNRS, UMR 8576 - UGSF - Unité de Glycobiologie Structurale et Fonctionnelle, F-59000 Lille, France Fax +33 (0) 320 43 65 55 E-mail: julie.bouckaert @univ-lille1.fr

† T.C. Shiao, Q. Wang, A. Papadopoulos, L. Mousavifar, R. Roy

Pharmaqam, Department of Chemistry, Université du Québec à Montréal, P. O. Box 8888, Succ. Centre-ville, Montréal, Québec, H3C 3P8, Canada

Fax: (+1) 514-987-4054, E-mail: roy.rene@uqam.ca

⊥ A. Glinschert, S. Oscarson

Center for Synthesis and Chemical Biology (CSCB), University College Dublin, Belfield, Dublin 4, Ireland

Corresponding Authors

Phone: +1 514987 3000 ext 2546. Email: roy.rene@uqam.ca;

Phone: +33 362 53 17 29. E-mail: julie.bouckaert@univ-lille1.fr

TABLE OF FIGURES 4

TABLE OF SCHEMES .... ERROR! BOOKMARK NOT DEFINED.

SYNTHESIS OF *O*- AND *C*-LINKED MANNOPYRANOSIDE LIBRARIES 6

RATIONAL FOR DESIGN AND RESULTS OF THE SYNTHESES **6**
FULL DETAILS OF SYNTHETIC PROTOCOLS AND STRUCTURAL CHARACTERIZATION FOR COMPOUNDS 18-123 .... **12**
GENERAL METHODS 12
GENERAL PROCEDURE OF HECK COUPLING FOR SYNTHESIS OF PROTECTED O- AND C-MANNOSIDES **18-21**, **111-114** 12
GENERAL PROCEDURE FOR DE-O-ACETYLATION 12
GENERAL PROCEDURE FOR HYDROGENATION (**26-29**, **119-123**) 12
GENERAL PROCEDURE OF THE RADICAL ADDITION FOR SYNTHESIS OF PROTECTED SULFUR SERIES **30-34**, **101-105** 13
GENERAL PROCEDURE OF SONOGASHIRA COUPLING FOR SYNTHESIS OF PROTECTED O- AND C-MANNOSIDES **42-49**, **59-60** 13
GENERAL PROCEDURE FOR SYNTHESIS OF PROTECTED SULFAMIDE SERIES **64-69** 13
GENERAL PROCEDURE FOR SYNTHESIS OF PROTECTED AMIDE SERIES **81-98** 13
GENERAL PROCEDURE FOR DEBENZYLATION: SYNTHESIS OF UNPROTECTED AMIDES SERIES **91-96** 13
IMPROVED PROCEDURE FOR THE PREPARATION OF N-HEPTYL α-D-MANNOPYRANOSIDE **5** 14
PROTON NMR STRUCTURAL ANALYSIS OF ALL COMPOUNDS **14**
Compound **18** 14
Compound **19** 14
Compound **20** 15
Compound **21** 15
Compound **22** 15
Compound **23** 16
Compound **24** 16
Compound **25** 16
Compound **26** 17
Compound **27** 17
Compound **28** 17
Compound **29** 17
Compound **30** 18
Compound **31** 18
Compound **32** 18
Compound **33** 19
Compound **35** 18
Compound **34** 19
Compound **36** 20
Compound **37** 20
Compound **38** 20
Compound **39** 21
Compound **40** 21
Compound **41** 21
Compound **42** 21
Compound **43** 21
Compound **44** 22
Compound **45** 22
Compound **46** 22
Compound **47** 23
Compound **48** 23

Compound **49** 23
Compound **50** 24
Compound **51** 24
Compound **52** 24
Compound **53** 24
Compound **54** 25
Compound **55** 25
Compound **56** 25
Compound **57** 25
Compound **59** 26
Compound **60** 26
Compound **61** 26
Compound **62** 27
Compound **63** 27
Compound **64** 27
Compound **65** 27
Compound **66** 28
Compound **67** 28
Compound **68** 28
Compound **69** 29
Compound **70** 29
Compound **71** 29
Compound **72** 29
Compound **73** 30
Compound **74** 30
Compound **75** 30
Compound **76** 31
Compound **77** : full structural characterization 31
Compound **78:** full structural characterization 33
Compound **79** 37
Compound **80** 37
Compound **81** 37
Compound **82** 38
Compound **83** 38
Compound **84** 38
Compound **85** 39
Compound **86** 39
Compound **87** 39
Compound **88** 40
Compound **89** : full structural characterization 39
Compound **90** 42
Compound **91** 42
Compound **92** 42
Compound **93** 42
Compound **94** 43
Compound **95** 43
Compound **96** 43
Compound **97** 44
Compound **98** 43
Compound **99** 44
Compound **100** 45
Compound **101** 46
Compound **102** 46

Compound **103** 46
Compound **104** 47
Compound **105** 47
Compound **106** 47
Compound **107** 48
Compound **108** 48
Compound **109** 48
Compound **110** 48
Compound **111** 49
Compound **112** 49
Compound **113** 49
Compound **114** 50
Compound **115** 50
Compound **116** 49
Compound **117** : full structural characterization 50
Compound **118** 52
Compound **119** 54
Compound **120** 54
Compound **121** 54
Compound **122** 55
Compound **123** 55

SURFACE PLASMON RESONANCE SOLUTION AFFINITY 55

SYNTHESIS OF 8-AMINOOCTYL A-D-MANNOPYRANOSIDE (5B) AS A COMPETITIVE STEADY STATE BINDER 55
STEADY STATE AFFINITY MEASUREMENT WITH COMPOUND 8-AMINOOCTYL A-D-MANNOPYRANOSIDE (5B) 57
SOLUTION AFFINITY OF LIGANDS FOR THE FIMH ADHESIN 57
COMPETITION METHOD 57
SPR MEASUREMENTS AND SOLUTION AFFINITY FITS FOR LEAD COMPOUNDS OLIGOMANNOSE-3, N-HEPTYL A-D-MANNOPYRANOSIDE (5) AND 8-AMINOOCTYL A-D-MANNOPYRANOSIDE (5A) 57

HAEMAGGLUTINATION INHIBITION ASSAY (HAI) 60

COMPARISONS OF THE POTENCY OF COMPOUNDS USING INHIBITION ASSAYS BASED ON WHOLE CELL INTERACTIONS (HAI) *VS* BASED ON MOLECULAR INTERACTIONS EITHER VIA COMPETITION (SPR) OR DIRECT (ITC) 60

MOLECULAR DYNAMICS SIMULATIONS 61

REFERENCES 62

Index of Figures, Tables and Movies

FIGURE S1. 600 MHz ^{1}H- AND ^{13}C-NMR ($CDCl_3$) OF **77** 32

FIGURE S2. 600 MHz COSY ^{1}H-NMR ($CDCl_3$) OF **77** ILLUSTRATING THE PROTONS CORRELATION 32

FIGURE S3. 600 MHz HMQC-NMR ($CDCl_3$) OF **77** ILLUSTRATING THE PROTON-CARBON CORRELATION USED FOR THE TOTAL SIGNAL ASSIGNMENTS 33

FIGURE S4. 600 MHz 1D-NOE NMR ($CDCl_3$) OF **77** CLEARLY ILLUSTRATING THE CONNECTIVITY BETWEEN H1-H6A-H4, THUS ILLUSTRATING THAT IT IS IN A SKEWED-BOAT CONFORMATION 33

FIGURE S5-S8: CONFORMATIONAL ANALYSIS OF **78**..33-35

Figure S9-S10: Conformational analysis of **78**..40

Figure S11-S12: Conformational analysis of **98**..43-44

FIGURE S13. 600 MHZ ^{1}H-NMR ($CDCL_3$) OF **89** ILLUSTRATING THE COMPLETE SIGNAL ANALYSIS OF H1 AND ITS CONNECTIVITY (NOE) TO H6A AND H4, HENCE A SKEWED-BOAT CONFORMATION..50

FIGURE S14. 600 MHZ ^{1}H-NMR ($CDCL_3$) OF **89** WITH INSET SHOWING THE EXO-METHYLENE HAB SYSTEM..................51

FIGURE S15. HIGH FIELD (900 MHZ) NMR ROESY SPECTRA OF **117** IN DMSO-D_6...51

FIGURE S16-S17. ^{1}H-NMR ANALYSIS OF **117** IN DMSO-D_6..52

TABLE S1. RESULTS FROM THE DETAILED ANALYSIS OF 117 BY 600 MHZ NMR..53

FIGURE S18. STEADY STATE AFFINITY (K_D = 16.9 NM ± 0.4, CHI^2 = 3.37) OF FIMH LECTIN WITH **5B**.............................57

FIGURE S19. INHIBITION CURVES AND FITTED EQUILIBRIUM CONSTANTS FOR LEAD COMPOUNDS WITH FIMH..................58

FIGURE S20. EX. OF A LINEAR TITRATION CURVE (FUCHSIA) THAT DOES NOT FOLLOW A SIGMOIDAL FIT (BLACK LINE)......59

TABLE S2: COMPARISON OF AFFINITY TRENDS USING THREE COMPLEMENTARY METHODS..60

FIGURE S21. MOLECULAR DYNAMICS SIMULATIONS..61

MOVIE S1. 61

Index of Schemes

SCHEME S1. REAGENTS AND CONDITIONS: I), AR-I, $PD(OAC)_2$, $NAHCO_3$, BU_4NBR, DMF, 60°C, II) MEONA, MEOH, RT, III) H_2, $PD(OH)_2$/C MEOH, IV) R1SH, AIBN, DIOXANE, 80°C. 7

SCHEME S2. REAGENTS AND CONDITIONS: I) $PD(PPH_3)_2CL_2$, R-I, ET_3N, DMF, 60°C, II) MEONA, MEOH, RT; III) $PD(PPH_3)_2CL_2$, HC≡CCH_2OH (59) OR HC≡CCH_2OME (60), ET_3N, DMF, 60°C...8

SCHEME S3. REAGENTS AND CONDITIONS: I) R_1-SO_2CL, ET_3N, DMF, 60°C, II) MEONA, MEOH, RT. 9

SCHEME S4. REAGENTS AND CONDITIONS: I) O_3, CH_2CL_2-MEOH (8:2), -78°C, ME_2S; II) JONES REAGENT, ACETONE, RT; III) BOP, DIEPA, R_1NH_2, CH_2CL_2, RT; IV) H_2, $PD(OH)_2$/C MEOH; V) NH_3(L), LI, THF, -78°C, THEN C_5H_5N, AC_2O; VI) R'SH, AIBN, DIOXANE, 80°C; VII) MEONA, MEOH, RT; VIII) AR-I, $PD(OAC)_2$, $NAHCO_3$, BU_4NBR, DMF, 60°C; IX) O_3, CH_2CL_2, -78°C, ZN, ACOH; X) $KMNO_4$, *T*-BUOH. 11

SCHEME S5. REAGENTS: (I) BZCL, DMAP, PYRIDINE, 0° C → RT, 36 H, QUANT.; (II) HBR (33% IN ACOH), ACOH, 0° C → RT, 36 H; (III) AGOTF, DCM, 0° C → RT, 24 H, 85% OVER 2 STEPS; (IV) NAOME, MEOH, RT, 2 H, 95%; (V) PD/C, H_2, MEOH, (H-CUBE), 80%. 55

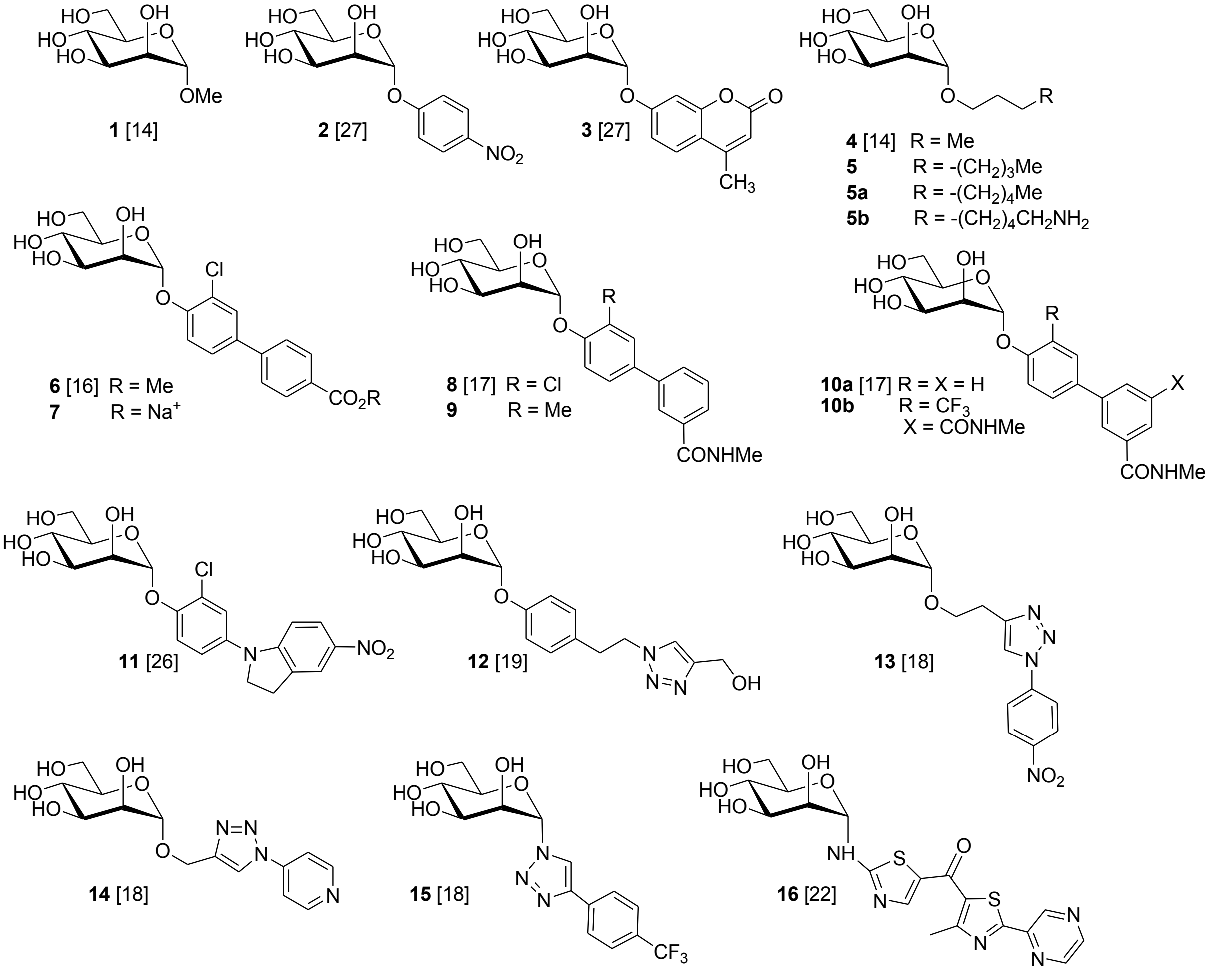

List of known FimH ligands

Synthesis of *O*- and *C*-linked mannopyranoside libraries

<u>Rational for design and results of the syntheses</u>

As shown in **Scheme S1**, key-starting material 3-(tetra-*O*-acetyl-α-D-mannopyranosyl)-1-propene **17** was converted to a series of (*E*)-styrene derivatives **18-21** in good yields. All acylated *O*-glycosides **18-21** were deprotected using a catalytic amount of sodium methoxide in methanol to provide unprotected derivatives **22-25** in almost quantitative yields. Catalytic hydrogenation of **22-25** was then used to afford saturated derivatives **26-29** in good to excellent yields. In parallel, treatment of *O*-allyl mannopyranoside **17** with a series of mercaptans in the presence of AIBN (thiol-ene) gave the sulfur-containing *O*-mannopyranosides **30-34** which after de-*O*-acetylation under Zemplén conditions (MeONa, MeOH) as above provided *O*-mannopyranosides **35-39**.

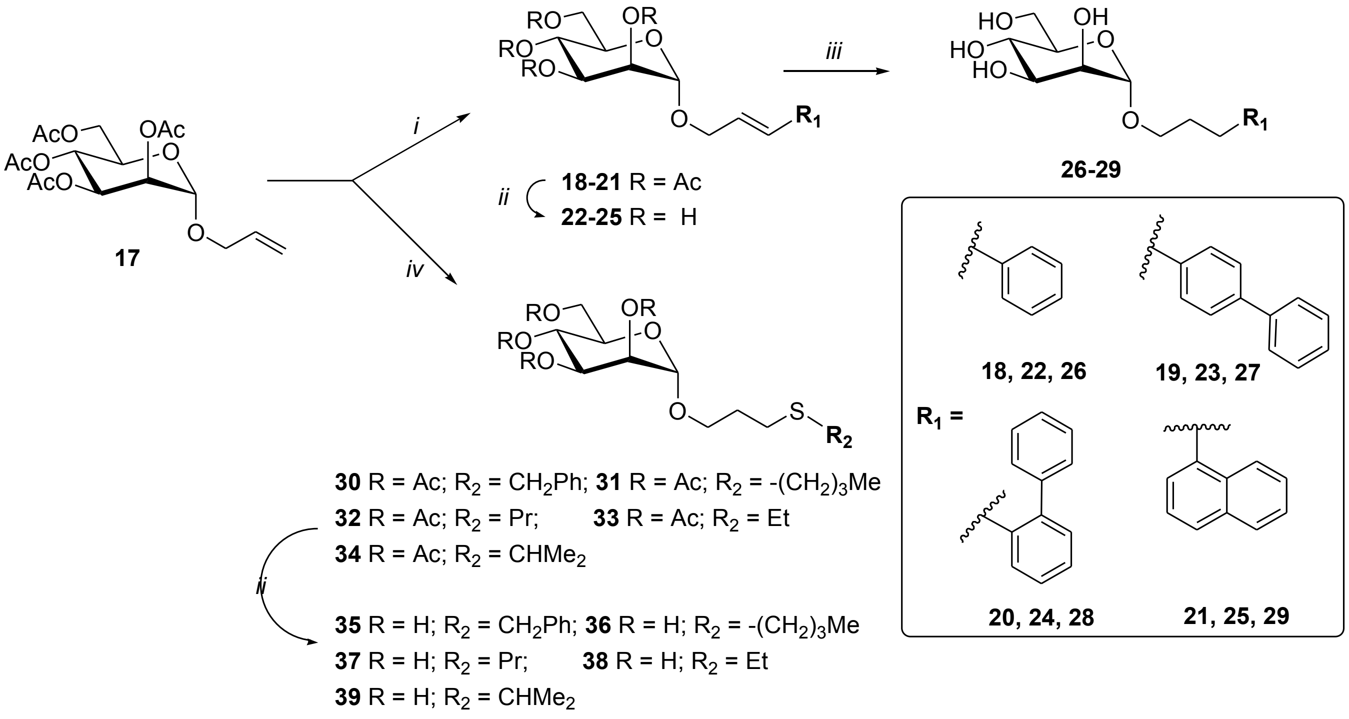

Scheme S1. Reagents and conditions: i), Ar-I, $Pd(OAc)_2$, $NaHCO_3$, Bu_4NBr, DMF, 60°C, ii) MeONa, MeOH, rt, iii) H_2, $Pd(OH)_2/C$ MeOH, iv) R1SH, AIBN, Dioxane, 80°C.

Given that alkyne spacers are even less flexible than the aliphatic or alkene linkages used above, we also considered using them to further exploit the relative positioning of the pharmacophoric aryl moieties within the tyrosine gate. Toward this goal, we selected alkynyl mannopyranoside **40** as the starting point for the next family of analogs. Thus, the use of Sonogashira cross-coupling conditions (triethylamine, copper iodide, $(Ph_3)_2PdCl_2$) with a range of iodoaryl derivatives and prop-2-ynyl 2,3,4,6-tetra-*O*-acetyl-α-D-mannopyranoside **40** [2,37] resulted into *O*-glycoside derivatives **42-49** in good yields. Their usual de-*O*-acetylation (MeONa, MeOH) uneventfully afforded the desired *O*-propargyl mannopyranoside derivatives **50-57** (**Scheme S2**).

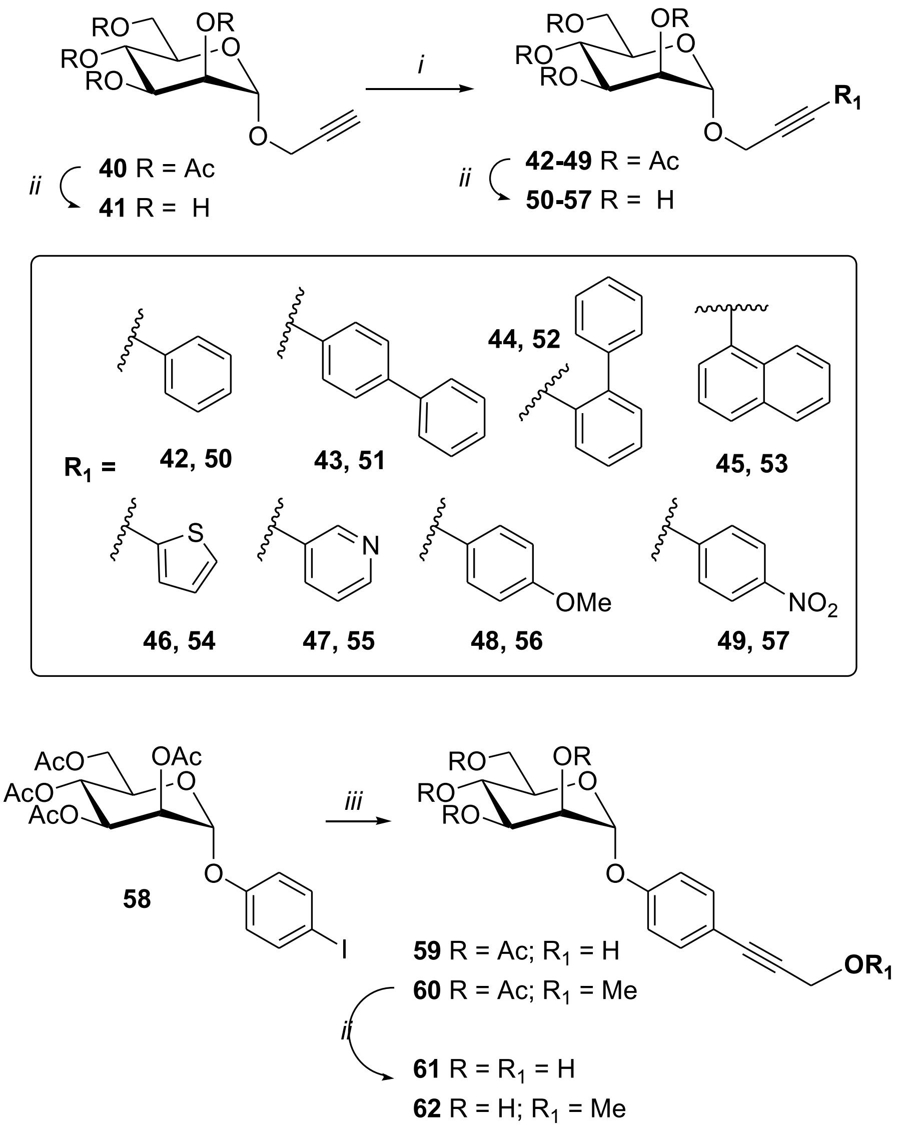

Scheme S2. Reagents and conditions: i) $Pd(PPh_3)_2Cl_2$, R-I, Et_3N, DMF, 60°C, ii) MeONa, MeOH, rt; iii) $Pd(PPh_3)_2Cl_2$, $HC{\equiv}CCH_2OH$ (**59**) or $HC{\equiv}CCH_2OMe$ (**60**), Et_3N, DMF, 60°C.

The effect of the relative positioning of the hydrophobic aglyconic residues upon binding to FimH and its tyrosine gate in particular was further deepened by interchanging the orders of alkyne-aryl introduction. To this end, analogs **61** and **62** were synthesized from the known p-iodophenyl 2,3,4,6-tetra-*O*-acetyl α-D-mannopyranoside **58** [38] using palladium-catalyzed Sonogashira cross-coupling (**Scheme 2**) and either propargyl alcohol or its methyl ether derivative.

Progressing toward the construction of other flexible *O*-linked ligands (family C, **Figure 2**), based on versatile 2-aminoethyl α-D-mannopyranoside **63**, may further increase our understanding of FimH *O*-

mannopyranosides with enhanced potencies. To this end, we next describe our efforts in identifying a novel class of sulfonamide containing *O*-mannopyranosides (**Scheme 3**). The new *O*-linked arylsulfonamides **64-69** were accordingly prepared by treatment of 2-aminoethyl 2,3,4,6-tetra-*O*-acetyl-α-D-mannopyranoside **63** [39] with the respective sulfonyl chlorides (**Scheme 3**), which upon standard de-*O*-acetylation (MeONa, MeOH) provided the expected analogs **70-75**.

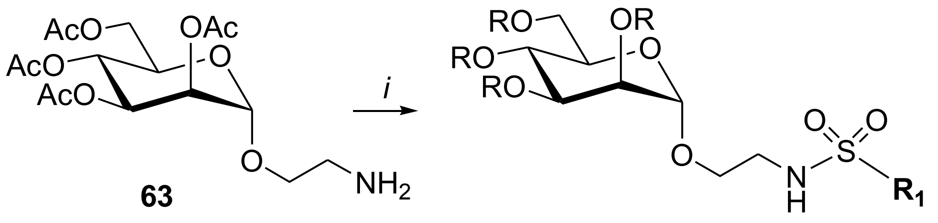

64 R = Ac; R_1 = Ph ; **65** R = Ac; R_1 = 4-NO_2Ph
66 R = Ac; R_1 = 4-MePh; **67** R = Ac; R_1 = 4-*t*BuPh
68 R = Ac; R_1 = 2,4,6-*i*Pr; **69** R = Ac; R_1 = 1-Naphthyl

ii

70 R = H; R_1 = Ph; **71** R = H; R_1 = 4-NO_2Ph
72 R = H; R_1 = 4-MePh; **73** R = H; R_1 = 4-*t*BuPh
74 R = H; R_1 = 2,4,6-*i*PrPh; **75** R = H; R_1 = 1-Naphthyl

Scheme S3. Reagents and conditions: i) R_1-SO_2Cl, Et_3N, DMF, 60°C, ii) MeONa, MeOH, rt.

Moving into the more biologically and chemically stable *C*-linked analogs (family D and E, **Figure 2**) and by similarity to their *O*-linked counterparts **25-29**, the next series of *C*-mannopyranoside derivatives have been synthesized according to **Scheme 4**. Key to the divergent synthesis of the inhibitors was the preparation of the peracetylated intermediate **77** from which several of the targeted compounds could be synthesized. Accordingly, *C*-allyl glycoside **77** was obtained as a pure α-anomer by debenzylation of known 3-(tetra-*O*-benzyl-α-D-mannopyranosyl)-1-propene **76** under Birch reduction conditions (Li, NH_3) and reprotection of the resulting alcohol with acetyl groups (Ac_2O, pyridine). This procedure was deemed necessary since inseparable mixture of anomers was obtained when the direct C-allylation was conducted from methyl tetra-O-acetyl-α-D-mannopyranoside under the Hosomi-Sakurai reaction conditions (BF_3, allyl trimethylsilane) [40]. Initially, ozonolysis of perbenzylated **76** followed by treatment with Jones reagent afforded the known 2-(2,3,4,6-tetra-O-benzyl-α-D-mannopyranosyl) acetic acid **79** [41]. As shown in **Scheme 4**, coupling with a systematic series of aromatic amines produced perbenzylated amides **81-86**, which could be readily converted to their corresponding unprotected mannopyranosides **91-96** by

classical benzyl deprotection (H_2,$Pd(OH)_2/C$). Analogously, peracetylated derivative **77** served as precursor toward known 2-(2,3,4,6-tetra-O-acetyl-α-D-mannopyranosyl) acetic acid **80**. As for perbenzylated amides **81-86**, coupling with a series of aromatic amines produced peracetylated amides **87-90**, which could be readily converted to unprotected *C*-mannosides **97-100** via acetyl deprotection (MeONa, MeOH).

In our ongoing effort to synthesize stable *C*-mannopyranosides that might surpass the potency of lead compound **5** (K_d = 5 nM), we next chose to prepare the nearest *C*-glycoside analogs **101-118** (family E, **Figure 2**) related to their *O*-linked counterparts (family A, **Figure 2**). Hence, the synthesis of sulfur-containing *C*-mannopyranosides harboring either aliphatic or aromatic substituents is summarized in **Scheme 4**. Key intermediate **77** was treated under thiol-ene conditions with a range of alkyl and aryl mercaptans in the presence of AIBN as radical initiator to give thioethers **101-105**, which upon standard de-*O*-acetylation under Zemplén conditions (MeONa, MeOH) afforded **106-110**. The next series was also prepared from **77** with the difference that the alkene was treated under palladium-catalyzed Heck conditions in the presence of aryl iodide to give (family E)-styrene derivatives **111-114**. Acylated glycosides **111-114** were deprotected using a catalytic amount of sodium methoxide in methanol as above to provide derivatives **115-118** in essentially quantitative yields. Finally, catalytic hydrogenation of *C*-glycosides **115-118** and **78** afforded their analogous saturated derivatives **119-122** and **123**, respectively in good yield.

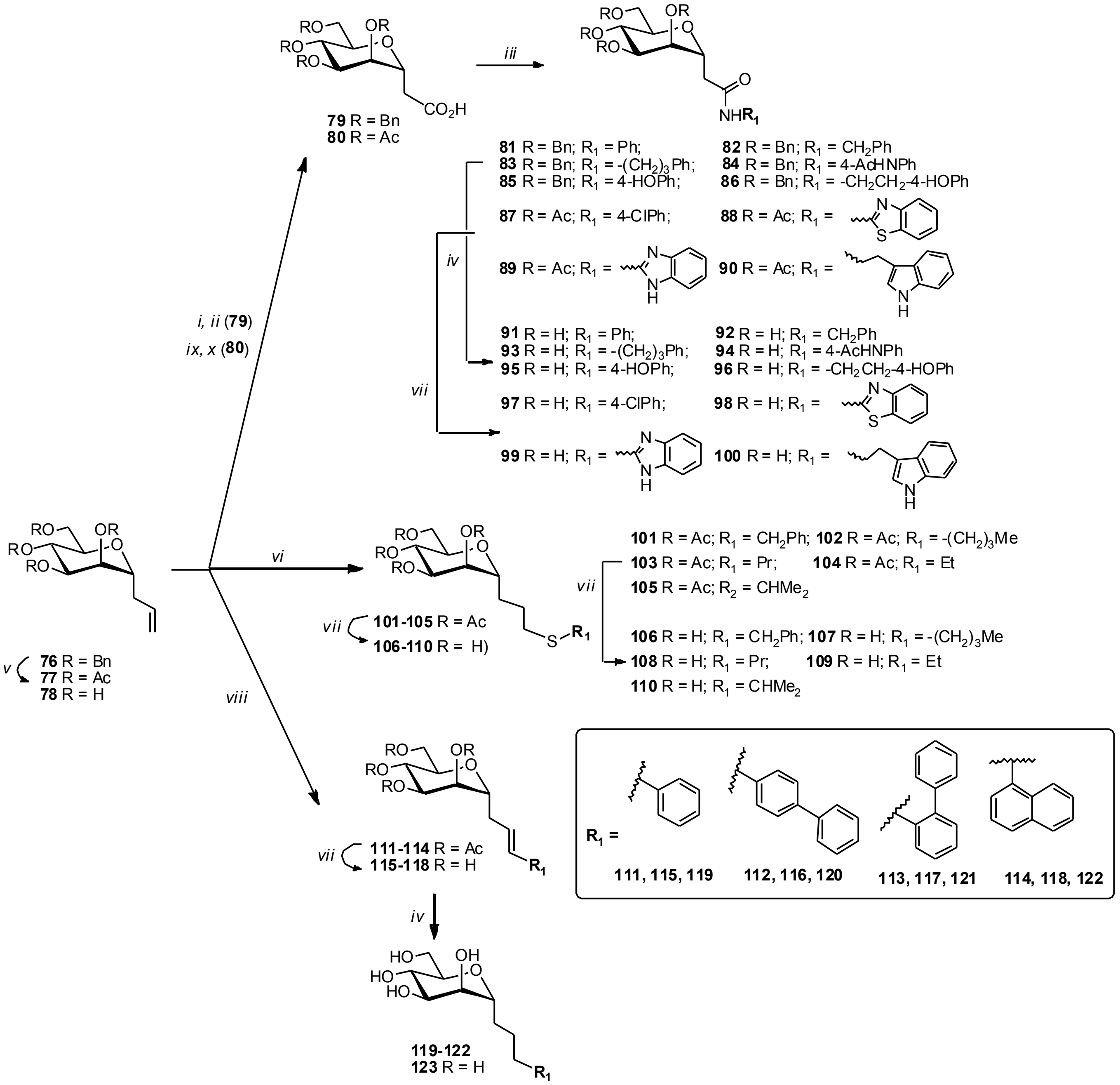

Scheme S4. Reagents and conditions: i) O_3, CH_2Cl_2-MeOH (8:2), -78°C, Me_2S; ii) Jones reagent, Acetone, rt; iii) BOP, DIEPA, R_1NH_2, CH_2Cl_2, rt; iv) H_2, $Pd(OH)_2$/C MeOH; v) NH_3(l), Li, THF, -78°C, then C_5H_5N, Ac_2O; vi) R'SH, AIBN, Dioxane, 80°C; vii) MeONa, MeOH, rt; viii) Ar-I, $Pd(OAc)_2$, $NaHCO_3$, Bu_4NBr, DMF, 60°C; ix) O_3, CH_2Cl_2, -78°C, Zn, AcOH; x) $KMnO_4$, *t*-BuOH.

Full details of synthetic protocols and structural characterization for compounds 18-123

General Methods

^{1}H NMR spectra were recorded using a Varian and Bruker ULTRASHIELD™ 300 MHz, Bruker Avance III HD 600 MHz, or Bruker Avance III 900 US2 spectrometers. Chemical shifts are reported in parts per million (d). ^{1}H Chemical shifts in $CDCl_3$ were referenced to the residual $CHCl_3$ (7.27 ppm); ^{13}C chemical shifts were referenced to the solvent ($CDCl_3$, 77.03 ppm). Where necessary, DEPT, APT, NOE, NOESY and two-dimensional ^{1}H-^{1}H COSY and HSQC experiments were performed for complete signal assignments. Accurate mass measurements were performed on a LC-MSD-Tof instrument from Agilent technologies in positive electrospray. Either protonated ions $(M+H)^+$ or sodium adducts $(M+Na)^+$ were used for empirical formula confirmation. Flash chromatography was performed using Merck silica gel 60 (40-63 μm). TLC was performed on Kieselgel 60 F254 plates from Merck. Detection was carried out under UV light or by spraying with 20% ethanolic sulfuric acid or molybdate solution followed by heating. Solvents were dried by distillation from drying agents as follows: THF and Et_2O (sodium/benzophenone), CH_2Cl_2 (P_2O_5), Et_3N and pyridine (CaH_2).

General procedure of Heck coupling for synthesis of protected *O*- and *C*-mannosides 18-21, 111-114

To a 0.16 M solution of mannoside **17** or **76** in DMF were added iodoaryl (2 equiv), 10% palladium(II) acetate, tetrabutylammonium bromide (1 equiv), and sodium bicarbonate (3 equiv). The reaction mixture was heated at 85 ^{0}C under N_2. The course of reaction was followed by TLC. The solution was evaporated under reduced pressure and the residue was purified by flash column chromatography on silica gel (0-20% AcOEt-Hexanes).

General procedure for de-*O*-acetylation

The acetylated acetylated mannoside was dissolved in dry MeOH (3 mL), a solution of sodium methoxide (1 M in MeOH, 0.5 equiv) was added and the reaction mixture was stirred at room temperature until disappearance of the starting material. The solution was neutralized by addition of ion-exchange resin (Amberlite IR 120), filtered, washed with MeOH and then the solvent was removed in vacuo. The residue was then lyophilized to yield the fully deprotected mannoside.

General procedure for hydrogenation (26-29, 119-123)

To a solution of a mixture of mannosides **22-25**, **78**, or **115-118** (0.06 mmol) in CH_3OH (1 mL) and AcOEt (1 mL) was added 20 wt % of $Pd(OH)_2/C$ (8 mg). The solution was stirred under 1 atm of H_2 for 4 h at room temperature. The catalyst was then removed by filtration through celite followed by washing

with CH_3OH. The filtrate was then concentrated and lyophilized to yield the fully hydrogenated mannoside.

General procedure of the radical addition for synthesis of protected sulfur series 30-34, 101-105

A mixture of allyl intermediate **17** or **77**, thiol (1.5 equiv), and AIBN (0.5 equiv) in dioxane (3 mL) was heated for 3 h at 80°C. The solution was cooled and evaporated to dryness. Sulfur derivatives were purified by column chromatography (0-20% AcOEt-Hexanes).

General procedure of Sonogashira coupling for synthesis of protected *O*- and *C*-mannosides 42-49, 59-60

To a solution of mannopyranoside **40** or **58**, dichlorobis(triphenylphosphine)palladium(II) (5 mol%)) and CuI (2.5 mol%) in 3 mL of dry DMF was degassed under nitrogen atmosphere using ultrasonic cleaner. After 5 min, propargyl alcool (2 equiv.) and triethylamine (3 mL) were added into the solution. The reaction mixture was stired at room temperature. The TLC (hexane/AcOEt 1:1) showed the complete dispearance of the starting material. The solvent and triethylamine were evaporated under reduced pressure. AcOEt (30 mL) was added to the reaction mixture and washed with saturated aqueous NH_4Cl (3x 50 mL), brine (3x 50 mL) and water (3x 50 mL). The organic layers were dried over $MgSO_4$ and evaporated under reduced pressure, the resulting crude product was purified by flash chromatography on silica gel (hexane/AcOEt 1:1) to give the desired mannoside.

General procedure for synthesis of protected sulfamide series 64-69

1-*O*-(2'-Aminoethyl)- 2,3,4,6-tetra-*O*-acetyl-α-d-mannopyranoside **63** and triethylamine (1.2 equiv) were dissolved in dry DCM. Sulfonyl chloride (1.2equiv per amine) was added to the mixture at 0°C. After stirring for 12 h at rt the solution was diluted with DCM and the organic layer washed with water and brine. After drying ($MgSO_4$) and concentration, amide derivatives were purified by column chromatography (0–10% MeOH-DCM).

General procedure for synthesis of protected amide series 81-98

Mannopyranosyl acid **79** or **80**, amine derivative (1.2 equiv as free amine or HCl salt) were dissolved in dry DCM. BOP (1.2equiv per amine) and DIPEA (3 equiv) were added to the mixture. After stirring for 16h at rt the solution was diluted with DCM and the organic layer washed with 1N $KHSO_4$, water and brine. After drying ($MgSO_4$) and concentration, amide derivatives were purified by column chromatography (0–5% MeOH-DCM).

General procedure for debenzylation: synthesis of unprotected amides series 91-96

To a solution of a mixture of benzylated mannoside (0.06 mmol) in CH_3OH (1 mL) and AcOEt (1 mL) was added 20 wt % of $Pd(OH)_2/C$ (8 mg). The solution was stirred under 1 atm of H_2 for 4 h at room

temperature. The catalyst was then removed by filtration through Celite followed by washing with CH_3OH. The filtrate was then concentrated and lyophilized to yield the fully deprotected product in a ~90% yield.

Improved procedure for the preparation of n-heptyl α-D-mannopyranoside 5

A solution of D-mannose (180 mg, 1.00 mmol) and a catalytic amount of CSA in 1-heptanol (2.0 mL) was heated under reflux for 2 h. Then, the reaction was kept at 120 °C for 5 h. The crude reaction mixture was directly purified by flash chromatography on silica gel using DCM-EtOAc-MeOH 8:1:1 or CH_3CN-H_2O (98:2) then lyophilized to give the title compound **4** as a white solid (217 mg, 0.78 mmol, 78%). R_f = 0.20, DCM-EtOAc-MeOH 8:1:1 ; R_f = 0.33, CH_3CN-H_2O 98:2 ; m.p.: 64-66 °C ; $[\alpha]^{23}_D$ + 58 (c=1.0, MeOH) ; ^{1}H NMR (600 MHz, D_2O, 23 °C): δ = 4.82 (sl, 1H, H-1), 3.92 (sl, 1H, H-2), 3.88-3.68 (m, 5H, H-3, 4, 6a and OCH_2), 3.55 (m, 1H, H-6b), 3.45 (m, 1H, H-5), 1.68-1.55 (m, 2H, OCH_2CH_2), 1.41-1.13 (m, 8H, 4 x CH_2), 0.91 ppm (t, 3H, $^3J_{H-H}$ = 6.5 Hz, CH_3) ; ^{13}C NMR (150 MHz, D_2O, 23 °C): δ = 99.5 (d, $^1J_{C-1,H1}$ = 169.3 Hz, C-1), 72.2 (C-5), 70.5 (C-2), 70.0 (C-3), 67.1 (C-4), 65.9 (OCH_2), 60.2 (C-6), 31.2, 28.8, 28.6, 25.5 (4 x CH_2), 22.1 (CH_2CH_3), 13.3 ppm (CH_3). ESI$^+$-HRMS: m/z calcd for $C_{13}H_{30}NO_6$ [M + $NH_4]^+$: 296.2068; found: 296.2065.

Proton NMR structural analysis of all compounds

Compound 18

Compound **18** was prepared according to general procedure for Heck cross coupling described above. Colourless oil, 102 mg, 86% yield. Rf: 0.52 (AcOEt: Hexanes 3: 7); $[\alpha]^{20}_D$ +57.5 (c = 1, $CHCl_3$). ^{1}H NMR (300 MHz, $CDCl_3$): δ 7.42-7.32 (5 H, m, Har), 6.66-6.61 (1 H, d, J = 15.6 Hz, H-3'), 6.31-6.22 (1 H, m, H-2'), 5.43-5.38 (1 H, dd, J = 3.4, 10.0 Hz), 5.36-5.28 (2 H, m), 4.943-4.937 (1 H, d, J = 1.6 Hz, H-1), 4.39-4.27 (2 H, m, H-1'), 4.24-4.20 (1 H, dd, J = 6.8, 12.6 Hz), 4.15-4.10 (1 H, dd, J = 2.4, 12.3 Hz), 4.09-4.04 (1 H, m), 2.16-2.00 (12 H, 4 x s, 4 x OAc); ^{13}C NMR (75 MHz, $CDCl_3$): δ 170.6, 169.9, 169.8, 169.7, 136.1, 134.0, 128.6, 128.0, 126.5, 123.8, 96.4, 69.6, 69.1, 68.5, 68.3, 66.1, 62.4, 20.8, 20.7, 20.6; MS (ESI): 487.32 $[M+Na]^+$, calcd. 487.16 for $C_{23}H_{28}O_{10} + Na^+$

Compound 19

Compound **19** was prepared according to general procedure for Heck cross coupling described above. Colourless oil, 285 mg, 82% yield. Rf: 0.31 (AcOEt: Hexanes 3: 7); $[\alpha]^{20}_D$ +17.3 (c = 1, $CHCl_3$). ^{1}H NMR (300 MHz, $CDCl_3$): δ 7.62-7.57 (4 H, m, Har), 7.49-7.45 (4 H, m, Har), 7.42-7.35 (1 H, m, Har), 6.66

(1H, d, J = 15.8 Hz, CH=CHPh), 6.35-6.28 (1 H, m, CH=CHPh), 5.44-5.29 (3 H, m, H-4, H-3, H-2), 4.96-4.95 (1 H, d, J = 1.6 Hz, H-1), 4.41-4.24 (2 H, m, H-5, H-6), 4.12-4.04 (3 H, m, H-6, OCH_2), 2.17, 2.13, 2.08, 2.06 (12 H, 4 x s, 4 x OAc); ^{13}C NMR (75 MHz, $CDCl_3$): δ 171.4, 170.7, 170.4, 170.2, 133.6, 128.8, 127.4, 127.3, 127.0, 126.9, 123.9, 118.5, 96.5, 69.6, 69.1, 68.6, 68.4, 66.2, 62.5, 20.9, 20.9, 20.7, 20.7; MS (ESI): 563.3 $[M+Na]^+$, calcd. 563.2 for $C_{29}H_{32}O_{10} + Na^+$

Compound 20

Compound **20** was prepared according to general procedure for Heck cross coupling described above. Colourless oil, 281 mg, 81% yield. Rf: 0.27 (AcOEt: Hexanes 3: 7); $[\alpha]^{20}_D$ +44.3 (*c* = 1, $CHCl_3$). ^1H NMR (300 MHz, $CDCl_3$): δ 7.65-7.62 (1 H, m, Har), 7.42-7.29 (8 H, m, Har), 6.61 (1H, d, J = 15.9 Hz, CH=CHPh), 6.25-6.16 (1 H, m, CH=CHPh), 5.41-5.26 (3 H, m, H-4, H-3, H-2), 4.88-4.78 (1 H, m, H-1), 4.29-4.25 (2 H, m, H-5, H-6), 4.14-3.79 (3 H, m, H-6, OCH_2), 2.15, 2.11, 2.08, 2.06 (12 H, 4 x s, 4 x OAc); ^{13}C NMR (75 MHz, $CDCl_3$): δ 170.57, 169.96, 169.78, 169.68, 141.04, 134.11, 133.29, 130.17, 129.70, 128.04, 127.89, 127.49, 127.07, 126.16, 124.78, 96.42, 69.61, 69.06, 68.57, 68.48, 66.19, 62.48, 20.83, 20.64, 20.58; MS (ESI): 563.3 $[M+Na]^+$, calcd. 563.2 for $C_{29}H_{32}O_{10} + Na^+$

Compound 21

Compound **21** was prepared according to general procedure for Heck cross coupling described above. Colourless oil, 430 mg, 93% yield. Rf: 0.52 (CH_2Cl_2-acetone 96:4); $[\alpha]^{20}_D$ +19.3 (*c* = 1, $CHCl_3$). ^1H NMR (300 MHz, $CDCl_3$): δ 8.10 (2 H, d, J = 7.7 Hz, Har), 7.87-7.79 (2 H, m, Har), 7.63-7.37 (5 H, m, 4 Har, CH=CHPh), 6.33-6.28 (1 H, m, CH=CHPh), 5.47-5.43 (3 H, m, H-4, H-3, H-2), 5.02-5.01 (1 H, m, H-1), 4.46-4.31 (2 H, m, H-5, H-6), 4.18-4.09 (3 H, m, H-6, OCH_2), 2.19, 2.15, 2.09, 2.03 (12 H, 4 x s, 4 x OAc); ^{13}C NMR (75 MHz, $CDCl_3$): δ 170.62, 170.0, 169.85, 169.63, 142.64, 140.31, 133.08, 131.04, 128.49, 128.26, 127.61, 127.13, 126.78, 125.51, 123.94, 109.42, 96.49, 69.61, 68.82, 68.35, 66.14, 62.47, 61.85, 20.85, 20.79, 20.69, 20.64; HRMS (ESI): 537.1722 $[M+Na]^+$, calcd. 537.1731 for $C_{27}H_{30}O_{10} + Na^+$

Compound 22

Compound **22** was prepared according to general procedure of de-*O*-acetylation described above. 33 mg, 95% yield. $[\alpha]^{20}_D$ +80.2 (*c* = 1, CH_3OH). ^1H NMR (300 MHz, CD_3OD): δ 7.41-7.38 (2 H, m, Har), 7.31-7.21 (3 H, m, Har), 6.66-6.61 (1 H, d, *J* = 15.9 Hz, CH=CHPh), 6.37-6.28 (1 H, td, *J* = 6.0, 15.9 Hz, CH=CHPh), 4.86 (1 H, H-1), 4.39-4.32 (1 H, d, *J* = 1.4 Hz, H-1), 4.20-4.13 (1 H, ddd, *J* = 12.9, 6.5 Hz, H-6), 3.88-3.81 (2 H, m, H-5, H-2), 3.77-3.70 (3 H, m, H-6, H-4, H-3), 3.66-3.60 (2 H, m, OCH_2); ^{13}C

NMR (75 MHz, $CDCl_3$): δ 138.1, 133.9, 129.6, 128.7, 127.5, 126.3, 100.7, 74.7, 72.6, 72.2, 68.6, 62.9; HMS (ESI): 319.1150 $[M+Na]^+$, calcd. 319.1152 for $C_{15}H_{20}O_6 + Na^+$

Compound 23

Compound **23** was prepared according to general procedure of de-*O*-acetylation described above. 22.5 mg, 94% yield. $[\alpha]^{20}_D$ +55.8 (c = 1, CH_3OH). ^1H NMR (300 MHz, DMSO-d6): δ 7.68-7.32 (9 H, m, Har), 6.64 (1 H, d, J = 16.2 Hz, CH=CHPh), 6.49-6.40 (1 H, m, CH=CHPh), 4.75-4.73 (1 H, m, H-1), 4.59-4.49 (2 H, m, H-2, H-3), 4.32-4.26 (1 H, m, H-4), 4.15-4.08 (1 H, m, H-6), 3.71-3.65 (3 H, m, H-5, OCH_2), 3.48-3.42 (1 H, m, H-6); ^{13}C NMR (75 MHz, DMSO-d6): δ 139.59, 139.22, 135.54, 131.07, 128.94, 127.46, 126.96, 126.82, 126.45, 99.21, 74.15, 71.01, 70.32, 67.07, 66.62, 61.31, HRMS (ESI): 359.1470 $[M+Na]^+$, calcd. 359.1465 for $C_{21}H_{24}O_6 + Na^+$

Compound 24

Compound **24** was prepared according to general procedure of de-*O*-acetylation described above. 38 mg, 93% yield. $[\alpha]^{20}_D$ +68.3 (c = 1, CH_3OH). ^1H NMR (300 MHz, DMSO-d6): δ 7.65-7.61 (1H, m, Har), 7.44-7.23 (8 H, m, Har), 6.58 (1 H, d, J = 15.9 Hz, CH=CHPh), 6.29-6.21 (1 H, m, CH=CHPh), 4.79 (1 H, d, J = 1.6 Hz, H-1), 4.25 (1 H, dd, J = 12.9, 5.6 Hz, H-6), 4.05 (1 H, dd, J = 12.9, 6.3 Hz, H-5), 3.80 (1 H, dd, J = 2.4, 11.8 Hz, H-2), 3.75-3.49 (3 H, m, H-6, H-4, H-3), 3.33-3.31 (2 H, m, OCH_2); ^{13}C NMR (75 MHz, DMSO-d6): δ 142.31, 142.19, 135.85, 132.54, 131.09, 130.74, 129.21, 128.70, 128.59, 128.19, 127.37, 127.12, 100.67, 74.67, 72.58, 72.58, 72.16, 68.61; HRMS (ESI): 395.1470 $[M+Na]^+$, calcd. 395.1465 for $C_{21}H_{24}O_6 + Na^+$

Compound 25

Compound **25** was prepared according to general procedure of de-*O*-acetylation described above. 166 mg, 90% yield. Rf: 0.32 (CH_2Cl_2-acetone-MeOH 8:1:1); $[\alpha]^{20}_D$ +48.2 (c = 1, CH_3OH). ^1H NMR (300 MHz, $CDCl_3$): δ 8.17-8.07 (1 H, m, Har), 7.93-7.83 (2 H, m, Har), 7.80-7.69 (1 H, m, Har), 7.58-7.29 (3 H, m, Har), 6.76 (1 H, d, J = 15.6 Hz, CH=CHPh), 6.51-6.32 (1 H, m, CH=CHPh), 4.88-4.82 (1 H, m, H-1), 4.62-4.53 (3 H, m, H-2, H-3, H-4), 4.22-4.13 (1 H, m, H-6), 3.79-3.69 (3 H, m, H-5, OCH_2), 3.53-3.49 (1 H, m, H-6); ^{13}C NMR (75 MHz, $CDCl_3$): δ 133.96, 133.63, 133.41, 131.49, 128.45, 128.13, 126.37, 126.14, 125.77, 123.53, 99.40, 72.44, 71.61, 71.01, 68.09, 66.22, 60.97 ppm ; HRMS (ESI): 369.1305 $[M+Na]^+$, calcd. 369.1309 for $C_{19}H_{22}O_6 + Na^+$

Compound 26

Compound 26 was prepared according to general procedure of hydrogenation described above. 36 mg, 97% yield. $[\alpha]^{20}_D$ +46.4 (*c* = 1, CH_3OH). ^1H NMR (300 MHz, CD_3OD): δ 7.27-7.14 (5 H, m, Har), 4.723-4.718 (1 H, d, *J* = 1.73 Hz, H-1), 3.81-3.59 (7 H, m, OCH_2, H-6, H-4, H-3, H-2), 3.55-3.52 (1 H, m, H-5), 2.71-2.65 (2 H, m, $OCH_2CH_2C\underline{H}_2$), 1.93-1.86 (2 H, m, $OCH_2C\underline{H}_2CH_2$); ^{13}C NMR (75 MHz, CD_3OD): δ 143.1, 129.4, 129.4, 126.8, 101.6, 74.6, 72.7, 72.2, 68.6, 67.8, 62.8, 33.4, 32.4; HRMS (ESI): 321.1301 $[M+Na]^+$, calcd. 321.1308 for $C_{15}H_{22}O_6 + Na^+$

Compound 27

Compound **27** was prepared according to general procedure of hydrogenation described above. 63 mg, 91% yield. $[\alpha]^{20}_D$ +21.8 (*c* = 1, CH_3OH). ^1H NMR (300 MHz, DMSO-d6): δ 7.47 (1 H, d, J = 7.9 Hz, Har), 7.41 (1 H, d, J = 7.7 Hz, Har), 3.27 (4 H, t, J = 7.8 Hz, Har), 7.18-7.11 (3 H, m, Har), 4.63-4.60 (1 H, m, H-1), 4.58-4.52 (2 H, m, H-2, H-3), 4.29 (1 H, bs, H-4), 3.45-3.17 (4 H, m, 2 H-6, H-5, OCH_2), 2.53-2.46 (2 H, m, $OCH_2CH_2C\underline{H}_2$), 1.72-1.65 (2 H, m, $OCH_2C\underline{H}_2CH_2$); ^{13}C NMR (75 MHz, DMSO-d6): δ 140.99, 140.09, 137.67, 128.90, 128.88, 127.14, 126.59, 126.47, 99.88, 74.03, 71.01, 70.39, 66.99, 61.24, 31.44, 30.76; HRMS (ESI): 397.1632 $[M+Na]^+$, calcd. 397.1621 for $C_{21}H_{26}O_6 + Na^+$

Compound 28

Compound **28** was prepared according to general procedure of hydrogenation described above. 63 mg, 91% yield. $[\alpha]^{20}_D$ +19.6 (*c* = 1, CH_3OH). ^1H NMR (300 MHz, DMSO-d6): δ 7.45-7.15 (9 H, m, Har), 4.48 (1 H, m, bs, H-1), 3.61-3.35 (4 H, m, H-5, H-4, H-3, H-2), 3.24-3.13 (4 H, m, H-6, OCH_2), 2.62-2.48 (2 H, m, $OCH_2CH_2C\underline{H}_2$), 1.67-1.61 (2 H, m, $OCH_2C\underline{H}_2CH_2$); ^{13}C NMR (75 MHz, DMSO-d6): δ 141.32, 141.22, 129.76, 129.28, 128.87, 128.19, 127.43, 12691, 125.88, 99.65, 73.86, 70.96, 70.27, 66.97, 65.59, 61.18, 30.51, 29.17; HRMS (ESI): 397.1609 $[M+Na]^+$, calcd. 397.1621 for $C_{21}H_{26}O_6 + Na^+$

Compound 29

Compound **29** was prepared according to general procedure of hydrogenation described above. 18.5 mg, 93% yield. $[\alpha]^{20}_D$ +52.3 (*c* = 1, CH_3OH). ^1H NMR (300 MHz, DMSO-d6): δ 8.07-8.04 (1 H, m, Har), 7.83-7.67 (2 H, m, Har), 7.51-7.38 (1 H, m, Har), 7.09-6.98 (3 H, m, Har), 4.87-4.83 (1 H, m, H-1), 3.87-3.76 (4 H, m, H-2, H-3, H-4, H-6), 3.74-3.58 (4 H, m, H-5, H-6, OCH_2), 2.77-2.62 (2 H, m, $OCH_2CH_2C\underline{H}_2$), 1.77-1.72 (2 H, m, $OCH_2C\underline{H}_2CH_2$); ^{13}C NMR (75 MHz, DMSO-d6): δ 140.95, 139.18,

138.22, 137.96, 135.43, 129.96, 128.40, 128.07, 127.26, 124.74, 101.54, 74.62, 72.25, 68.07, 62.83, 34.44, 24.64; HRMS (ESI): 371.1462 $[M+Na]^+$, calcd. 371.1465 for $C_{19}H_{24}O_6 + Na^+$

Compound 30

Compound **30** was prepared according to general procedure of radical addition described above. 274 mg, 83% yield. Rf: 0.19 (AcOEt: Hexanes 3: 7); $[\alpha]^{20}_D$ +29.6 (*c* = 1, $CHCl_3$). ^{1}H NMR (300 MHz, $CDCl_3$): δ 7.36-7.21 (5 H, m, Ph), 5.33-5.23 (2 H, m, H-3, H-4), 5.22 (1 H, dd, J = 1.9, 3.3 Hz, H-2), 4.77 (1 H, d, J = 1.6 Hz, H-1), 4.27 (1 H, dd, J = 5.2, 12.3 Hz, H-6), 4.09 (1 H, dd, J = 2.2, 12.1 Hz, H-6), 3.97-3.93 (1 H, m, H-5), 3.80-3.73 (1 H, m, OCHH), 3.70 (2 H, s, SCH_2), 3.52-3.45 (1 H, m, OCHH), 2.51 (2 H, t, J = 7.1 Hz, CH_2S), 2.15-1.99 (12 H, 4 x s, 4 x OAc), 1.86-1.81 (2 H, m, OCH_2CH_2); ^{13}C NMR (75 MHz, $CDCl_3$): δ 170.48, 169.88, 169.71, 169.55, 138.16, 128.66, 128.36, 126.85, 97.41, 69.38, 68.93, 68.35, 66.41, 65.95, 62.26, 36.06, 28.49, 27.66, 20.74, 20.59, 20.53; MS (ESI): 535.4 $[M+Na]^+$, calcd. 535.5 for $C_{24}H_{32}O_{10}S + Na^+$

Compound 31

Compound **31** was prepared according to general procedure of radical addition described above. 114 mg, 88% yield. Rf: 0.22 (AcOEt: Hexanes 4: 6); $[\alpha]^{20}_D$ +38.4 (*c* = 1, $CHCl_3$). ^{1}H NMR (300 MHz, $CDCl_3$): δ 5.32 (1 H, dd, J = 3.5, 9.6 Hz, H-4), 5.30-5.22 (2 H, m, H-, H-2), 4.81-4.80 (1 H, d, J = 1.6 Hz, H-1), 4.28 (1 H, dd, J = 5.2, 12.3 Hz, H-6), 4.10 (1 H, dd, J = 2.4, 12.3 Hz, H-6), 4.02-3.97 (1 H, m, H-5), 3.81 (1 H, dt, J = 6.0, 9.6 Hz, OCHH), 3.53 (1 H, dt, J = 6.0, 9.7 Hz, OCHH), 2.61 (2 H, t, J = 6.6 Hz, CH_2S), 2.52 (2 H, t, J = 7.1 Hz, SCH_2), 2.15-1.99 (12 H, 4 x s, 4 x OAc), 1.94-1.85 (2 H, m, OCH_2CH_2), 1.61-1.51 (2 H, m, SCH_2CH_2), 1.46-1.36 (2 H, m, $SCH_2CH_2CH_2$), 0.91 (3 H, t, J = 7.4 Hz, $SCH_2CH_2CH_2CH_3$); ^{13}C NMR (75 MHz, $CDCl_3$): δ 170.64, 170.04, 169.86, 169.70, 97.56, 69.56, 69.08, 68.48, 66.56, 66.12, 62.42, 31.77, 31.65, 28.98, 28.53, 21.95, 20.85, 20.72, 20.66, 13.63; MS (ESI): 501.29 $[M+Na]^+$, calcd. 501.18 for $C_{21}H_{34}O_{10}S + Na^+$

Compound 32

Compound **32** was prepared according to general procedure of radical addition described above. 220 mg, 73% yield. Rf: 0.22 (AcOEt: Hexanes 3: 7); $[\alpha]^{20}_D$ +42.8 (*c* = 1, $CHCl_3$). ^{1}H NMR (300 MHz, $CDCl_3$): δ 5.29-5.18 (3 H, m, H-2, H-3, H-4), 4.77 (1 H, s, H-1), 4.23 (1 H, dd, J = 3.8, 11.0 Hz, H-6), 4.06 (1 H, d, J = 12.1 Hz, H-6), 3.98-3.94 (1 H, m, H-5), 3.82-3.74 (1 H, m, OCHH), 3.53-3.46 (1 H, m, OCHH), 2.56 (2 H, t, J = 8.1 Hz, CH_2S), 2.47-2.42 (2 H, m, SCH_2), 2.11-1.94 (12 H, 4 x s, 4 x OAc), 1.88-1.82 (2 H, dd, J

= 6.6, 12.3 Hz, $OCH_2C\underline{H_2}$), 1.60-1.53 (2 H, dd, J = 7.7, 14.56 Hz, $C\underline{H_2}CH_3$), 0.97-0.92 (3 H, t, J = 7.42 Hz, $CH_2C\underline{H_3}$); ^{13}C NMR (75 MHz, $CDCl_3$): δ 170.48, 169.89, 169.72, 169.56, 97.42, 69.42, 68.95, 68.36, 66.39, 65.98, 62.28, 33.99, 28.86, 28.32, 22.74, 20.73, 20.60, 20.53, 13.32; MS (ESI): 465.1 $[M+H]^+$, calcd. 464.9 for $C_{20}H_{32}O_{10}S + H^+$

Compound 33

Compound **33** was prepared according to general procedure of radical addition described above. 112.5 mg, 88% yield. Rf: 0.19 (AcOEt: Hexanes 3: 7); $[\alpha]^{20}_D$ +48.4 (*c* = 1, $CHCl_3$). ^{1}H NMR (300 MHz, $CDCl_3$): δ 5.33-5.20 (3 H, m, H-2, H-3, H-4), 4.79 (1 H, d, J = 1.65 Hz, H-1), 4.26 (1 H, dd, J = 5.2, 12.3 Hz, H-6), 4.09 (1 H, dd, J = 2.2, 12.1 Hz, H-6), 4.01-3.95 (1 H, m, H-5), 3.80 (1 H, dt, J = 5.7, 9.9 Hz, $OC\underline{H}H$), 3.52 (1 H, dt, J = 6.0, 9.8 Hz, $OCH\underline{H}$), 2.60 (2 H, t, J = 6.6 Hz, $SC\underline{H_2}CH_3$), 2.52 (2 H, dd, J = 7.4, 14.5 Hz, $OCH_2CH_2C\underline{H_2}$), 2.14-1.97 (12 H, 4 x s, 4 x OAc), 1.93-1.83 (2 H, m, $OCH_2C\underline{H_2}$), 1.24 (3 H, t, J = 6.6 Hz, $SCH_2C\underline{H_3}$); ^{13}C NMR (75 MHz, $CDCl_3$): δ 170.57, 169.98, 169.81, 169.65, 97.50, 69.51, 69.03, 68.46, 66.50, 66.08, 62.38, 28.87, 27.98, 25.80, 20.80, 20.67, 20.60, 20.61, 14.63; MS (ESI): 473.31 $[M+Na]^+$, calcd. 473.14 for $C_{19}H_{30}O_{10}S + Na^+$

Compound 34

Compound **34** was prepared according to general procedure of radical addition described above. 245 mg, 82% yield. Rf: 0.19 (AcOEt: Hexanes 3: 7); $[\alpha]^{20}_D$ +30.4 (*c* = 1, $CHCl_3$). ^{1}H NMR (300 MHz, $CDCl_3$): δ 5.31 (1 H, dd, J = 3.57, 9.89 Hz, H-4), 5.29-5.25 (1 H, m, H-3), 5.21 (1 H, dd, J = 1.6, 3.3 Hz, H-2), 4.79 (1 H, d, J = 1.9 Hz, H-1), 4.29-4.23 (1H, dd, J = 5.5, 12.3 Hz, H-6), 4.12-4.07 (1 H, dd, J = 2.2, 13.1 Hz, H-6), 4.02-3.97 (1 H, m, H-5), 3.81 (1 H, dt, J = 6.8, 9.6 Hz, $OC\underline{H}H$), 3.51 (1 H, dt, J = 6.3, 9.8 Hz, $OCH\underline{H}$), 2.89 (1 H, q, J = 7.6 Hz, SCH), 2.61 (2 H, dt, J = 2.2, 7.4 Hz, CH_2S), 2.14-1.97 (12 H, 4 x s, 4 x OAc), 1.93-1.82 (2 H, m, $OCH_2C\underline{H_2}$), 1.25 (6 H, dd, J = 1.1, 6.8 Hz, 2 x CH_3); ^{13}C NMR (75 MHz, $CDCl_3$): δ 170.57, 169.99, 169.81, 169.65, 97.48, 69.52, 69.04, 68.44, 66.51, 66.08, 62.37 (OCH_2), 34.80 (SCH), 29.09, 26.87, 23.29, 23.28, 20.82, 20.69, 20.62; MS (ESI): 465.0 $[M+H]^+$, calcd. 464.9 for $C_{20}H_{32}O_{10}S + H^+$

Compound 35

Compound **35** was prepared according to general procedure of de-*O*-acetylation described above. 35 mg, 91% yield. $[\alpha]^{20}_D$ +46.6 (*c* = 1, CH_3OH). ^{1}H NMR (300 MHz, D_2O): δ 6.99-6.89 (5 H, m, Har), 4.57 (1 H, m, H-1), 3.71-3.54 (6 H, m, 2 H-6, H-5, H-2, $OC\underline{H_2}$), 3.45-3.42 (2 H, m, H-4, H-3), 3.34 (2 H, s, $C\underline{H_2}Ph$),

2.16 (2 H, t, J = 6.8 Hz, CH_2S), 1.52-1.48 (2 H, m, $OCH_2C\underline{H_2}$); ^{13}C NMR (75 MHz, D_2O): δ 134.93, 128.41, 128.04, 126.51, 99.32, 72.23, 70.41, 69.80, 65.99, 65.43, 60.30, 34.96, 28.10, 27.12; HRMS (ESI): 367.1187 $[M+Na]^+$, calcd. 367.1185 for $C_{16}H_{24}O_6S + Na^+$

Compound 36

Compound **36** was prepared according to general procedure of de-*O*-acetylation described above. 59 mg, 92% yield. $[\alpha]^{20}_D$ +19.6 (*c* = 1, CH_3OH). ^1H NMR (300 MHz, D_2O): δ 4.74 (1 H, bs, H-1), 3.80-3.79 (1 H, m, H-6), 3.70-3.55 (5 H, m, H-6, H-4, H-3, H-2), 3.47-3.42 (2 H, m, OCH_2), 2.49 (2 H, t, J = 7.1 Hz, CH_2S), 2.42 (2 H, t, J = 7.3 Hz, SCH_2), 1.79-1.74 (2 H, m, $OCH_2C\underline{H_2}$), 1.49-1.40 (2 H, m, $SCH_2C\underline{H_2}$), 1.34-1.25 (2 H, m, $SCH_2CH_2C\underline{H_2}$), 0.79 (3 H, t, J = 7.4 Hz, CH_3); ^{13}C NMR (75 MHz, D_2O): δ 99.45, 72.26, 70.36, 69.81, 65.99, 65.68, 60.30, 30.95, 30.80, 28.63, 27.79, 21.27, 12.92; HRMS (ESI): 333.1342 $[M+Na]^+$, calcd. 333.1342 for $C_{13}H_{26}O_6S + Na^+$

Compound 37

Compound **37** was prepared according to general procedure of de-*O*-acetylation described above. 66 mg, 92% yield. $[\alpha]^{20}_D$ +19.3 (*c* = 1, CH_3OH). ^1H NMR (300 MHz, D_2O): δ δ 4.71 (1 H, bs, H-1), 3.79-3.75 (3 H, m, H-6, H-5, H-2), 3.71-3.63 (3 H, m, H-6, H-4, H-3), 3.59-3.41 (2 H, m, $OC\underline{H_2}$), 2.51 (2H, t, J = 7.1 Hz, $OCH_2CH_2C\underline{H_2}$), 2.41 (2 H, t, J = 7.2 Hz, $SC\underline{H_2}CH_2CH_3$), 1.78-1.74 (2 H, m, $OCH_2C\underline{H_2}CH_2$), 1.52-1.39 (2 H, m, $SCH_2C\underline{H_2}CH_3$), 0.81 (3 H, t, J = 7.2 Hz, $SCH_2CH_2C\underline{H_3}$); ^{13}C NMR (75 MHz, D_2O): δ 99.31, 72.29, 70.21, 69.67, 66.21, 65.70, 60.41, 32.92, 28.23, 27.39, 21.87, 12.36; HRMS (ESI): 297.1375 $[M+H]^+$, calcd. 297.1366 for $C_{12}H_{24}O_6S + H^+$

Compound 38

Compound **38** was prepared according to general procedure of de-*O*-acetylation described above. 52 mg, 93% yield. $[\alpha]^{20}_D$ +21.8 (*c* = 1, CH_3OH). ^1H NMR (300 MHz, D_2O): δ 4.73 (1 H, bs, H-1), 3.81-3.79 (2 H, m, H-6, H-2), 3.77-3.60 (4 H, m, H-6, H-5, H4, H-3), 3.53-3.43 (2 H, m, $OC\underline{H_2}$), 2.53 (2 H, t, J = 7.1 Hz, $OCH_2CH_2C\underline{H_2}$), 2.45 (2 H, q, J = 7.4 Hz, $SC\underline{H_2}CH_3$), 1.82-1.73 (2 H, m, $OCH_2C\underline{H_2}CH_2$), 1.10 (3 H, t, J = 7.4 Hz, $SCH_2C\underline{H_3}$); ^{13}C NMR (75 MHz, D_2O): δ 99.27, 72.28, 70.21, 69.66, 66.22, 65.71, 60.42, 28.13, 26.93, 24.73, 13.51; HRMS (ESI): 305.1034 $[M+Na]^+$, calcd. 305.1020 for $C_{11}H_{22}O_6S + Na^+$

Compound 39

Compound **39** was prepared according to general procedure of de-*O*-acetylation described above. 131 mg, 94% yield. $[\alpha]^{20}_{D}$ +41.2 (c = 1, CH_3OH). ^{1}H NMR (300 MHz, D_2O): δ 4.72 (1 H, bs, H-1), 3.80-3.78 (1 H, m, H-6), 3.75 (1 H, dd, J = 9.8, 7.9 Hz, H-2), 3.69-3.61 (2 H, m, H-6, H-5), 3.57-3.41 (2 H, m, H-4, H-3), 2.88 (1 H, sep, J = 6.8 Hz, SCH), 2.54 (2 H, t, J = 7.4 Hz, CH_2S), 1.78-1.75 (2 H, m, OCH_2CH_2), 1.25 (6 H, d, J = 6.6 Hz, 2 x CH_3); ^{13}C NMR (75 MHz, D_2O): δ 99.29, 72.25, 70.15, 69.61, 66.17, 65.77, 60.37, 34.14, 28.33, 25.93, 22.06; HRMS (ESI): 319.1191 $[M+Na]^+$, calcd. 319.1185 for $C_{12}H_{24}O_6S$ + Na^+

Compound 40 was prepared according to a published procedure [1].

Compound 41

Compound **41** was prepared according to general procedure of de-*O*-acetylation described above. White solid, mp 122-123 °C, 93 mg, 83% yield. $[\alpha]^{20}_{D}$ +19.6 (c = 1, CH_3OH).^{1}H NMR (300 MHz, CD_3OD): δ 4.95 (1 H, d, J = 1.4 Hz, H-1), 4.26 (2 H, d, J = 2.4 Hz OCH_2), 3.85-3.77 (2 H, m, H-6, H-5), 3.72-3.65 (2 H, m, H-6, H-2), 3.57-3.49 (2 H, m, H-4, H-3); ^{13}C NMR (75 MHz, CD_3OD): δ 99.8, 80.0, 75.9, 75.1, 72.5, 72.0, 68.5, 62.8, 54.8; MS (ESI): calcd for $C_9H_{14}O_6Na$ $[M + Na]^+$: 241.1 ; found : 241.1.

Compound 42

Compound **42** was prepared according to general procedure for Sonogashira coupling described above. Colourless oil, 08 mg, 82% yield. Rf: 0.19 (AcOEt: Hexanes 3: 7); $[\alpha]^{20}_{D}$ +18.3 (c = 1, $CHCl_3$). ^{1}H NMR (300 MHz, $CDCl_3$): δ 7.46-7.43 (2 H, m, Har), 7.34-7.29 (3 H, m, Har), 5.39 (1 H, dd, J = 10.1 3.4 Hz, H-3), 5.37-5.29 (2 H, m, H-4, H-2), 5.12 (1 H, bs, H-1), 4.50 (2 H, s, OCH_2), 4.31 (1 H, dd, J = 12.1 5.2 H-6), 4.14-4.05 (2 H, m, H-6, H-5), 2.17, 2.09, 2.03, 2.01 (12 H, 4 x s, 4 x OAc); ^{13}C NMR (75 MHz, $CDCl_3$): δ 170.64, 169.92, 169.84, 169.69, 131.81, 128.75, 128.32, 122.05, 96.19, 87.14, 83.10, 69.42, 68.98, 68.95, 66.04, 62.32, 55.70, 20.86, 20.69, 20.67, 20.64; MS (ESI): 485.41 $[M+Na]^+$, calcd. 485.14 for $C_{23}H_{26}O_{10}$ + Na^+

Compound 43

Compound **43** was prepared according to general procedure for Sonogashira cross coupling described above. Colourless oil, 148 mg, 71% yield. Rf: 0.23 (AcOEt: Hexanes 3: 7); $[\alpha]^{20}_{D}$ +12.7 (c = 1, $CHCl_3$). ^{1}H NMR (300 MHz, $CDCl_3$): δ 7.79-7.34 (9H, m, Har), 5.44-5.30 (3H, m, H-3, 4, 2), 5.15 (1H, d, J = 1.6

Hz, H-1), 4.53 (2H, s, OC*H*$_2$), 4.33 (1H, dd, J = 12.2, 5.0 Hz, H-6a), 4.17-4.07 (2H, m, H-6b and H-5), 2.18, 2.11, 2.06, 2.01 (12H, 4 x s, OAc) ; ^{13}C NMR (75 MHz, CDCl$_3$): δ 170.6, 169.9, 169.8, 169.7, 141.5, 140.2, 138.2, 132.2, 128.8, 127.7, 127.0 120.9, 96.2, 87.0, 83.7, 69.4, 69.0, 69.0, 66.1, 62.3, 55.8, 20.9, 20.7, 20.7, 20.6; MS (ESI): 561.18 $[M+Na]^+$, calcd. 561.18 for $C_{29}H_{30}O_{10}$ + Na^+

Compound 44

Compound **44** was prepared according to general procedure for Sonogashira cross coupling described above. Colourless oil, 88 mg, 66% yield. Rf: 0.19 (AcOEt: Hexanes 3: 7); $[\alpha]^{20}_D$ +19.8 (*c* = 1, CHCl$_3$). ^{1}H NMR (300 MHz, CDCl$_3$): δ 7.57-7.54 (3 H, m, Har), 7.44-7.29 (6 H, m, Har), 5.33 (1 H, dd, J = 10.1, 2.9 Hz, H-3), 5.28-5.25 (2 H, m, H-4, H-2), 4.93 (1 H, d, J = 1.6 Hz, H-1), 4.38 (2 H, s, OC$\underline{H_2}$), 4.25 (1 H, dd, J = 12.1, 5.1 H-6), 4.03 (1 H, dd, J = 12.4, 2.3 Hz, H-5), 3.97-3.93 (1 H, m, H-6), 2.17, 2.05, 2.03, 2.01 (12 H, 4 x s, 4 x OAc); ^{13}C NMR (75 MHz, CDCl$_3$): δ 170.59, 169.81, 169.65, 143.99, 142.16, 133.21, 129.50, 129.09, 128.90, 127.91, 127.51, 126.99, 120.38, 95.89, 86.76, 85.83, 69.28, 69.03, 68.80, 65.95, 62.28, 55.41, 20.86, 20.65, 20.63; MS (ESI): 561.31 $[M+Na]^+$, calcd. 561.17 for $C_{29}H_{30}O_{10}$+ Na^+

Compound 45

Compound **45** was prepared according to general procedure for Sonogashira cross coupling described above. Colourless oil, 144 mg, 72% yield. Rf: 0.19 (AcOEt: Hexanes 3: 7); $[\alpha]^{20}_D$ +22.3 (*c* = 1, CHCl$_3$). ^{1}H NMR (300 MHz, CDCl$_3$): δ 8.28 (1 H, d, J = 7.9 Hz, Har), 7.84 (2 H, d, J = 8.2 Hz, Har), 7.59-7.40 (3 H, m, Har), 5.41 (1 H, dd, J = 9.8, 3.1 Hz, H-3), 5.37-5.29 (2 H, m, H-4, H-2), 5.23 (1 H, d, J = 1.4 Hz, H-1), 4.66 (2 H, s, OC$\underline{H_2}$), 4.32 (1 H, dd, J = 12.6, 5.2 H-6), 4.16-4.12 (2 H, m, H-5, H-6), 2.16, 2.10, 2.05, 2.01 (12 H, 4 x s, 4 x OAc); ^{13}C NMR (75 MHz, CDCl$_3$): δ 170.59, 169.87, 169.83, 169.65, 133.21, 133.02, 130.89, 129.23, 128.23, 126.93, 126.45, 125.91, 125.08, 119.63, 96.24, 78.89, 85.23, 69.42, 68.99, 66.02, 62.30, 55.84, 20.82, 20.64, 20.62; MS (ESI): 513.18 $[M+H]^+$, calcd. 513.17 for $C_{27}H_{28}O_{10}$ + H^+

Compound 46

Compound **46** was prepared according to general procedure for Sonogashira cross coupling described above. Colourless oil, 65 mg, 53% yield. Rf: 0.27 (AcOEt: Hexanes 1: 1); $[\alpha]^{20}_D$ +43.6 (*c* = 1, CHCl$_3$). ^{1}H NMR (300 MHz, CDCl$_3$): δ 7.26-7.21 (1 H, m, H-3`), 7.23-7.21 (1 H, m, H-5`), 6.97-6.93 (1 H, m, H-4`), 5.35 (1 H, dd, J = 10.1, 3.2 Hz, H-3), 5.37-5.26 (2 H, m, H-4, H-2), 5.06 (1 H, d, J = 1.1 Hz, H-1), 4.48 (2 H, s, OC$\underline{H_2}$), 4.32 (1 H, dd, J = 12.3, 4.9 H-6), 4.08 (1 H, dd, J = 12.1, 2.4 Hz, H-5), 4.07-4.04 (1 H, m,

H-6), 2.14, 2.11, 2.05, 2.02 (12 H, 4 x s, 4 x OAc); ^{13}C NMR (75 MHz, $CDCl_3$): δ 170.45, 169.81, 169.74, 169.59, 132.84, 127.77, 126.93, 121.79, 96.28, 87.15, 80.37, 69.26, 68.91, 65.89, 62.19, 55.73, 20.77, 20.63, 20.59, 20.55; MS (ESI): 491.34 $[M+Na]^+$, calcd. 491.10 for $C_{21}H_{24}O_{10}S + Na^+$

Compound 47

Compound **47** was prepared according to general procedure for Sonogashira cross coupling described above. Colourless oil, 112 mg, 94% yield. Rf: 0.27 (AcOEt: Hexanes 1: 1); $[\alpha]^{20}_D$ +13.7 (*c* = 1, $CHCl_3$).^{1}H NMR (300 MHz, $CDCl_3$): δ 8.61 (1H, d, J = 1.3 Hz, Har), 8.49 (1H, dd, J = 4.9, 1.5 Hz, Har), 7.68 (1H, m, Har), 7.23-7.19 (1H, m, Har), 5.34-5.21 (3H, m, H-3,4,2), 5.04 (1H, d, J = 1.5 Hz, H-1), 4.45 (2H, m, OCH_2), 4.24 (1H, dd, J = 12.2, 5.1 Hz, H-6a), 4.08-3.97 (2H, m, H-6b and H-5), 2.10, 2.03, 1.99, 1.93 (4 x s, OAc); ^{13}C NMR (75 MHz, $CDCl_3$): δ 170.5, 169.9, 169.8, 169.6, 152.3, 150.0, 138.8, 123.0, 119.2, 96.1, 86.6, 83.8, 69.3, 69.0, 68.8, 65.9, 62.2, 55.4, 20.8, 20.6, 20.6, 20.6; MS (ESI): 464.32 $[M+H]^+$, calcd. 464.16 for $C_{22}H_{25}NO_{10} + H^+$

Compound 48

Compound **48** was prepared according to general procedure for Sonogashira cross coupling described above. Colourless oil, 81 mg, 63% yield. Rf: 0.21 (AcOEt: Hexanes 3: 7); $[\alpha]^{20}_D$ +31.6 (*c* = 1, $CHCl_3$). ^{1}H NMR (300 MHz, $CDCl_3$): δ 7.38 (2 H, dd, J = 6.8, 2.2 Hz, Har), 6.83 (2 H, dd, J = 6.8, 2.2 Hz, Har), 5.37 (1 H, dd, J = 9.9, 3.3 Hz, H-3), 5.34-5.30 (2 H, m, H-4, H-2), 5.11 (1 H, d, J = 1.6 Hz, H-1), 4.48 (2 H, s, O$C\underline{H_2}$), 4.29 (1 H, dd, J = 12.6, 5.2 H-6), 4.13-4.03 (2 H, m, H-6, H-5), 3.81 (3 H, s, O$C\underline{H_3}$), 2.16, 2.08, 2.04, 2.01 (12 H, 4 x s, 4 x OAc); ^{13}C NMR (75 MHz, $CDCl_3$): δ 170.64, 169.91, 169.83, 169.68, 159.92, 133.34, 114.08, 113.93, 96.10, 87.13, 81.73, 69.43, 69.01, 68.89, 66.02, 62.30, 55.78, 55.24, 20.85, 20.69, 20.66, 20.63; MS (ESI): 515.27 $[M+Na]^+$, calcd. 515.15 for $C_{24}H_{28}O_{11} + Na^+$

Compound 49

Compound **49** was prepared according to general procedure for Sonogashira cross coupling described above. Colourless oil, 187 mg, 85% yield. Rf: 0.13 (AcOEt: Hexanes 3: 7); $[\alpha]^{20}_D$ +24.8 (*c* = 1, $CHCl_3$). ^{1}H NMR (300 MHz, $CDCl_3$): δ 8.19 (2 H, dd, J = 6.8, 2.2 Hz, Har), 7.60 (2 H, dd, J = 6.8, 2.2 Har), 5.38 (1 H, dd, J = 9.8, 3.1 Hz, H-3), 5.35-5.29 (2 H, m, H-4, H-2), 5.09 (1 H, d, J = 1.6 Hz, H-1), 4.58-4.46 (2 H, bs, O$C\underline{H_2}$), 4.30 (1 H, dd, J = 12.3, 5.1 H-6), 4.12 (1 H, dd, J = 12.3, 2.4 Hz, H-5), 4.07-4.02 (1 H, m, H-6), 3.81 (3 H, s, O$C\underline{H_3}$), 2.17, 2.11, 2.05, 2.02 (12 H, 4 x s, 4 x OAc); ^{13}C NMR (75 MHz, $CDCl_3$): δ 170.54, 169.95, 169.85, 169.62, 147.39, 132.55, 128.82, 123.57, 96.23, 88.40, 85.19, 69.35, 69.07,

68.801, 65.93, 62.26, 55.39, 20.84, 20.68, 20.64, 20.62; MS (ESI): 530.32 [M+Na]$^+$, calcd. 530.13 for $C_{23}H_{25}NO_{12}$ + Na^+

Compound 50

Compound **50** was prepared according to general procedure of de-*O*-acetylation described above. 12 mg, 86% yield. $[\alpha]^{20}_D$ +43.2 (*c* = 1, CH_3OH). ^{1}H NMR (300 MHz, CD_3OD): δ 7.43-7.38 (2 H, m, Har), 7.35-7.31 (3 H, m, Har), 5.03 (1 H, d, J = 1.6 Hz, H-1), 4.49 (2 H, s, $OC\underline{H_2}$), 3.87-3.82 (2 H, m, H-6, H-5), 3.74-3.63 (2 H, m, H-6, H-2), 3.60-3.53 (2 H, m, H-4, H-3); ^{13}C NMR (75 MHz, CD_3OD): δ 132.69, 129.66, 129.50, 123.88, 99.97, 87.08, 85.43, 75.14, 72.51, 72.08, 68.48, 62.84, 55.54; MS (ESI): 611.30 [M+Na]$^+$, calcd. 611.21 for $C_{15}H_{18}O_6$ + Na^+

Compound 51

Compound **51** was prepared according to general procedure of de-*O*-acetylation described above. 69 mg, 87% yield. $[\alpha]^{20}_D$ +19.2 (*c* = 1, CH_3OH). ^{1}H NMR (300 MHz, DMSOd$_6$): δ 7.70-7.67 (3 H, m, Har), 7.55-7.35 (6 H, m, Har), 4.85 (1 H, bs, H-1), 4.54-4.52 (2 H, m, $OC\underline{H_2}$), 3.71-3.64 (2 H, m, H-6, H-5), 3.49-3.35 (4 H, m, H-6, H-4, H-3, H-2); ^{13}C NMR (75 MHz, DMSOd$_6$): δ 140.37, 139.07, 132.09, 129.05, 127.94, 126.89, 126.67, 120.82, 98.36, 86.34, 85.40, 74.51, 70.94, 70.12, 66.87, 61.14, 53.73; HRMS (ESI): 393.1313 [M+Na]$^+$, calcd. 393.1308 for $C_{21}H_{22}O_6$ + Na^+

Compound 52

Compound **52** was prepared according to general procedure of de-*O*-acetylation described above. 49 mg, 91% yield. $[\alpha]^{20}_D$ +9.1 (*c* = 1, CH_3OH). ^{1}H NMR (300 MHz, CD_3OD): δ 7.54-7.51 (3 H, m, Har), 7.44-7.28 (6 H, m, Har), 4.91 (1 H, d, J = 1.6 Hz, H-1), 4.36 (2 H, bs, $OC\underline{H_2}$), 3.80 (1 H, dd, J = Hz, H-5), 3.76-3.70 (2 H, m, H-6, H-2), 3.64-3.46 (3 H, m, H-6, H-4, H-3); ^{13}C NMR (75 MHz, CD_3OD): δ 145.37, 141.73, 134.27, 130.58, 130.16, 129.94, 129.12, 128.65, 128.18, 122.04, 99.64, 88.45, 86.73, 75.01, 72.50, 72.03, 68.45, 62.81, 55.26; MS (ESI): 339.40 [M+Na]$^+$, calcd. 339.39 for $C_{21}H_{22}O_6$ + Na^+

Compound 53

Compound **53** was prepared according to general procedure of de-*O*-acetylation described above. 69 mg, 87% yield. $[\alpha]^{20}_D$ +21.3 (*c* = 1, CH_3OH). ^{1}H NMR (300 MHz, CD_3OD): δ 8.17 (1 H, dd, J = 7.9, 0.8 Hz, Har), 7.78-7.72 (2 H, m, Har), 7.55 (1 H, dd, J = 7.1, 1.1 Har), 7.49-7.30 (3 H, m, Har), 5.04 (1 H, d, J = 1.6 Hz, H-1), 4.56 (2 H, s, $OC\underline{H_2}$), 3.79-3.75 (2 H, m, H-6, H-5), 3.67-3.60 (2 H, m, H-6, H-2), 3.58-3.52

(2 H, m, H-4, H-3); ^{13}C NMR (75 MHz, CD_3OD): δ 134.66, 134.53, 132.61, 131.73, 130.16, 129.43, 129.23, 129.15, 128.76, 127.97, 127.76, 127.58, 126.82, 126.27, 121.27, 100.06, 90.54, 85.06, 75.22, 72.55, 72.12, 68.50, 62.86, 55.70, 55.63; HRMS (ESI): 345.13329 $[M+H]^+$, calcd. 345.13326 for $C_{21}H_{22}O_6 + H^+$

Compound 54

Compound **54** was prepared according to general procedure of de-*O*-acetylation described above. 37 mg, 92% yield. $[\alpha]^{20}_D$ +41.2 (c = 1, CH_3OH). ^{1}H NMR (300 MHz, CD_3OD): δ 7.41-7.38 (1 H, m, H-3\`), 7.23-7.21 (1 H, m, H-5\`), 7.01-6.98 (1 H, m, H-4\`), 5.0 (1 H, d, J = 1.4 Hz, H-1), 4.51 (2 H, s, OC$\underline{H_2}$), 3.78-3.81 (2 H, m, H-6, H-5), 3.72-3.63 (2 H, m, H-6, H-2), 3.57-3.52 (2 H, m, H-4, H-3); ^{13}C NMR (75 MHz, CD_3OD): δ 133.67, 128.84, 128.16, 123.44, 100.08, 89.42, 80.25, 75.15, 72.49, 72.03, 68.43, 62.80, 55.63; MS (ESI): 323.30 $[M+Na]^+$, calcd. 323.32 for $C_{13}H_{16}O_6S + Na^+$

Compound 55

Compound **55** was prepared according to general procedure of de-*O*-acetylation described above. 29 mg, 93% yield. $[\alpha]^{20}_D$ +46.2 (c = 1, CH_3OH). ^{1}H NMR (300 MHz, CD_3OD): δ 8.62-8.52 (2 H, m, H-3\`, H-6\`), 7.88 (1 H, d, J = 7.7 Hz, H-4\`), 7.44-7.41 (1 H, m, H-5\`), 5.02 (1 H, bs, H-1), 4.53 (2 H, s, OC$\underline{H_2}$), 3.85 (1 H, dd, J = 9.1, 1.9 Hz, H-5), 3.73-3.64 (2 H, m, H-6, H-2), 3.63-3.57 (3 H, m, H-6, H-4, H-3); ^{13}C NMR (75 MHz, CD_3OD): δ - 152.60, 149.48, 140.76, 125.13, 100.23, 89.71, 83.24, 75.22, 72.47, 72.00, 68.46, 62.85, 55.45, 30.73; MS (ESI): 296.40 $[M+H]^+$, calcd. 296.30 for $C_{14}H_{17}NO_6 + H^+$

Compound 56

Compound **56** was prepared according to general procedure of de-*O*-acetylation described above. 26 mg, 93% yield. $[\alpha]^{20}_D$ +11.7 (c = 1, CH_3OH). ^{1}H NMR (300 MHz, CD_3OD): δ 7.34 (2 H, d, J = 8.7 Hz, Har), 6.86 (2 H, d, J = 8.7 Hz, Har),, 5.03 (1 H, bs, H-1), 4.65 (2 H, s, OC$\underline{H_2}$), 3.86-3.82 (2 H, m, H-6, H-5), 3.78 (3 H, s, OC$\underline{H_3}$), 3.73-3.63 (2 H, m, H-6, H-2), 3.60-3.55 (2 H, m, H-4, H-3); ^{13}C NMR (75 MHz, CD_3OD): δ 161.85, 134.08, 115.88, 113.58, 99.46, 86.68, 83.96, 74.78, 72.29, 72.06, 68.12, 62.53, 56.06, 55.94; MS (ESI): 347.4 $[M+Na]^+$, calcd. 347.1 for $C_{16}H_{20}O_7 + Na^+$

Compound 57

Compound **57** was prepared according to general procedure of de-*O*-acetylation described above. 77.8 mg, 88% yield. $[\alpha]^{20}_D$ +22.3 (c = 1, CH_3OH). ^{1}H NMR (300 MHz, CD_3OD): δ 8.22 (2 H, d, J = 8.2 Hz,

Har), 7.66 (2 H, d, J = 8.2 Hz, Har), 5.01 (1 H, d, J = 1.6 Hz, H-1), 4.55 (2 H, s, OC$\underline{H_2}$), 3.87-3.82 (2 H, m, H-6, H-5), 3.73-3.67 (2 H, m, H-6, H-2), 3.58-3.56 (2 H, m, H-4, H-3); ^{13}C NMR (75 MHz, CD_3OD): δ - 148.79, 133.70, 130.55, 124.673, 100.325, 90.90, 85.21, 75.24, 72.48, 72.01, 68.47, 62.85, 55.48; MS (ESI): 3632.3 $[M+Na]^+$, calcd. 362.1 for $C_{15}H_{17}NO_8 + Na^+$

Compound 59

Compound **59** was prepared according to general procedure for Sonogashira coupling described above 106 mg, 98%. R_f = 0.28 (hexane/AcOEt 1:1); mp 63-64°C; $[\alpha]^{20}_D$ +70.3 (*c* 1.0, $CHCl_3$). ^{1}H NMR ($CDCl_3$) *δ* 7.38 (d, 2H, J = 8.8 Hz, H_{ar}), 7.03 (d, 2H, J = 8.8 Hz, H_{ar}), 5.54 (dd, 1H, J = 3.3, 10.0 Hz, H-3), 5.52 (d, 1H, J = 1.9 Hz, H-1), 5.43 (dd, 1H, $J_{1,2}$ 1.9 Hz, J = 3.3 Hz, H-2), 5.35 (t, 1H, J = 10.0 Hz, H-4), 4.48 (d, 2H, J = 6.1Hz, CCH_2O), 4.27 (dd, 1H, J = 5.9, 12.6 Hz, H-6a), 4.08-4.03 (m, 2H, H-5 and H-6b), 1.94 (t, 1H, J = 6.1 Hz, OH), 2.20, 2.05, 2.04, 2.03 ppm (4s, 4x3H, $COCH_3$); ^{13}C NMR ($CDCl_3$) *δ* 170.5, 170.0, 169.9, 169.7, 155.5, 133.2, 117.2, 116.4, 95.6, 86.7, 84.9, 69.2, 69.2, 68.7, 65.8, 62.0, 51.5, 20.8-20.6 ppm; MS (ESI): 479.1 $[M+H]^+$, calcd. 479.1 for $C_{23}H_{26}O_{11} + H^+$

Compound 60

Compound **60** was prepared according to general procedure for Sonogashira cross coupling described above. Colourless oil, 105 mg, 94% yield. Rf: 0.5 (hexane/AcOEt 1:1); $[\alpha]^{20}_D$ +43.3 (*c* = 1, $CHCl_3$). ^{1}H NMR (300 MHz, $CDCl_{3,}$ $CDCl_3$): δ 7.39 (2 H, d, J = 8.7 Hz, Har), 7.02 (2 H, d, J = 8.7 Hz, Har), 5.55-5.43 (2 H, m, H-3, H-1), 5.43-5.31 (2 H, m, H-4, H-2), 4.29 (2 H, s, OC$\underline{H_2}$), 4.27 (1 H, dd, J = 12.3, 6.04 Hz, H-6), 4.07-4.03 (2 H, m, H-6, H-5), 3.43 (3 H, s, OC$\underline{H_3}$), 2.19, 2.05, 2.03, 2.02 (12 H, 4 x s, 4 x OAc); ^{13}C NMR (75 MHz, $CDCl_{3,}$ $CDCl_3$): δ 170.44, 169.89, 169.86, 169.66, 155.44, 133.21, 117.26, 116.35, 95.85, 85.62, 84.30, 69.24, 69.20, 68.71, 65.81, 62.01, 60.35, 57.62, 20.78, 20.62, 20.59; MS (ESI): 493.0 $[M+H]^+$, calcd. 493.1 for $C_{24}H_{28}O_{11} + H^+$

Compound 61

Compound 61 was prepared according to general procedure of de-*O*-acetylation described above. 58 mg, 91% yield. $[\alpha]^{20}_D$ +38.6 (*c* = 1, CH_3OH). ^{1}H NMR (300 MHz, CD_3OD): δ 7.36-7.31 (2 H, m, Har), 7.08-7.04 (2 H, m, Har), 5.49 (1 H, d, J = 1.6 Hz, H-1), 4.36 (2 H, s, CC$\underline{H_2}$O), 3.99-3.81 (1 H, m, H-3), 3.87 (1 H, dd, J = 9.3, 3.4 Hz, H-5), 3.76-3.66 (3 H, m, H-6, H-4, H-2), 3.57-3.53 (1 H, m, H-6); ^{13}C NMR (75 MHz, CD_3OD): δ - 157.84, 134.00, 118.04, 117.69, 99.97, 87.72, 85.15, 75.47, 72.32, 71.83, 68.23, 62.59, 51.21; HRMS (ESI): 333.09488 $[M+Na]^+$, calcd. 333.09447 for $C_{15}H_{18}O_7 + Na^+$

Compound 62

Compound **62** was prepared according to general procedure of de-*O*-acetylation described above. 49 mg, 93% yield. $[\alpha]^{20}_{D}$ +32.8 (c = 1, CH_3OH). ^1H NMR (300 MHz, CD_3OD): δ 7.38-7.33 (2 H, m, Har), 7.10-7.05 (2 H, m, Har), 5.50 (1 H, d, J = 1.9 Hz, H-1), 4.28 (2 H, s, $CC\underline{H}_2O$), 4.0-3.98 (1 H, m, H-3), 3.87 (1 H, dd, J = 9.3, 3.2 Hz, H-5), 3.77-3.63 (3 H, m, H-6, H-4, H-2), 3.57-3.51 (1 H, m, H-6); ^{13}C NMR (75 MHz, CD_3OD): δ - 157.97, 134.12, 117.71, 117.65, 99.93, 86.91, 84.75, 75.47, 72.30, 71.80, 68.20, 62.55, 61.01, 57.72; HRMS (ESI): 347.11012 $[M+H]^+$, calcd. 347.11076 for $C_{16}H_{20}O_7 + Na^+$

Compound 63 was prepared according to a published procedure [2].

Compound 64

Compound **64** was prepared according to general procedure for sulfamide synthesis described above. Colourless oil, 68 mg, 89% yield. Rf: 0.27 (MeOH: CH_2Cl_2 5: 95); $[\alpha]^{20}_{D}$ +48.3 (c = 1, $CHCl_3$). ^1H NMR (300 MHz, $CDCl_3$): δ 7.91-7.86 (2 H, m, Har), 7.62-7.53 (3 H, m, Har), 4.25-5.19 (3 H, m, H-4, H-3, H-2), 4.71 (1 H, d, J = 1.3 Hz, H-1), 4.24 (1 H, dd, J = 12.3, 5.3 Hz, H-6), 4.06 (1 H, dd, J = 12.3, 2.4 Hz, H-5), 3.95-3.89 (1 H, m, H-6), 3.79-3.72 (1 H, m, $OC\underline{H}_2CH_2N$), 3.52-3.47 (1 H, m, $OC\underline{H}_2CH_2N$), 3.32-3.17 (2 H, m, $OCH_2C\underline{H}_2N$), 2.11, 2.08, 2.06, 2.04 (12 H, 4 x s, 4 x OAc); ^{13}C NMR (75 MHz, $CDCl_3$): δ 170.62, 169.93, 169.63, 139.96, 132.74, 129.21, 126.93, 69.14, 68.86, 68.73, 67.25, 65.90, 62.35, 42.63, 20.78, 20.68, 20.636; MS (ESI): 554.37 $[M+Na]^+$, calcd. 554.13 for $C_{22}H_{29}NO_{12}S + Na^+$

Compound 65

Compound **65** was prepared according to general procedure for sulfamide synthesis described above. Colourless oil, 78 mg, 71% yield. Rf: 0.22 (MeOH: CH_2Cl_2 10: 90); $[\alpha]^{20}_{D}$ +16.8 (c = 1, $CHCl_3$). ^1H NMR (300 MHz, $CDCl_3$): δ 8.41-8.36 (2 H, m, Har), 8.11-8.07 (2 H, m, Har), 5.46-5.44 (1 H, m, NH), 5.29-5.14 (3 H, m, H-4, H-3, H-2), 4.72 (1 H, d, J = 1.3 Hz, H-1), 4.25 (1 H, dd, J = 12.3, 5.3 Hz, H-6), 4.09 (1 H, dd, J = 12.3, 2.7 Hz, H-5), 3.93-3.87 (1 H, m, H-6), 3.83-3.80 (1 H, m, $OC\underline{H}_2CH_2N$), 3.56-3.50 (1 H, m, $OC\underline{H}_2CH_2N$), 3.32-3.21 (2 H, m, $OCH_2C\underline{H}_2N$), 2.15, 2.07, 2.05, 2.0 (12 H, 4 x s, 4 x OAc); ^{13}C NMR (75 MHz, $CDCl_3$): δ 170.66, 170.15, 170.02, 169.63, 150.131, 145.89, 128.25, 124.55, 97.73, 69.03, 68.85, 68.79, 67.02, 65.93, 62.44, 42.82, 20.78, 20.71, 20.64; MS (ESI): 599.3 $[M+Na]^+$, calcd. 599.1 for $C_{22}H_{28}N_2O_{14}S + Na^+$

Compound 66

Compound **66** was prepared according to general procedure for sulfamide synthesis described above. Colourless oil, 48 mg, 69% yield. Rf: 0.27 (MeOH: CH_2Cl_2 5: 95); $[\alpha]^{20}_{D}$ +29.4 (c = 1, $CHCl_3$). ^{1}H NMR (300 MHz, $CDCl_3$): δ 7.75 (2 H, d, J = 8.1 Hz, Har), 7.31 (2 H, d, J = 8.1 Hz, Har), 5.25-5.20 (3 H, m, H-4, H-3, H-2), 5.13-5.09 (1 H, m, NH), 4.71 (1 H, d, J = 1.3 Hz, H-1), 4.25 (1 H, dd, J = 12.1, 5.2 Hz, H-6), 4.09 (1 H, dd, J = 12.1, 2.3 Hz, H-5), 3.95-3.91 (1 H, m, H-6), 3.78-3.71 (1 H, m, $OC\underline{H_2}CH_2N$), 3.52-3.45 (1 H, m, $OC\underline{H_2}CH_2N$), 3.20-3.16 (2 H, m, $OCH_2C\underline{H_2}N$), 2.42 (3 H, s, $C\underline{H_3}$), 2.12, 2.11, 2.09, 2.07 (12 H, 4 x s, 4 x OAc); ^{13}C NMR (75 MHz, $CDCl_3$): δ 170.64, 169.92, 169.65, 143.57, 136.91, 129.81, 126.99, 97.78, 69.13, 68.85, 68.70, 67.28, 65.88, 62.33, 42.61, 29.63, 21.48, 20.80, 20.69, 20.65, 20.63; MS (ESI): 568.35 $[M+Na]^+$, calcd. 568.14 for $C_{23}H_{31}NO_{12}S + Na^+$

Compound 67

Compound **67** was prepared according to general procedure for sulfamide synthesis described above. Colourless oil, 68.6 mg, 91% yield. Rf: 0.20 (MeOH: CH_2Cl_2 5: 95); $[\alpha]^{20}_{D}$ +16.3 (c = 1, $CHCl_3$). ^{1}H NMR (300 MHz, $CDCl_3$): δ 7.81-7.77 (2 H, m, Har), 7.55-7.52 (2 H, m, Har), 5.26-5.20 (3 H, m, H-4, H-3, H-2), 5.13-5.11 (1 H, m, NH), 4.73 (1 H, d, J = 1.3 Hz, H-1), 4.25 (1 H, dd, J = 12.2, 5.3 Hz, H-6), 4.07 (1 H, dd, J = 12.2, 2.3 Hz, H-5), 3.98-3.94 (1 H, m, H-6), 3.80-3.74 (1 H, m, $OC\underline{H_2}CH_2N$), 3.54-3.47 (1 H, m, $OC\underline{H_2}CH_2N$), 3.21-3.15 (2 H, m, $OCH_2C\underline{H_2}N$), 2.14, 2.09, 2.05, 2.02 (12 H, 4 x s, 4 x OAc), 1.32 (9 H, s, $C(CH_3)_3$); ^{13}C NMR (75 MHz, $CDCl_3$): δ 170.63, 169.93, 169.65, 156.54, 136.75, 126.82, 126.19, 69.16, 68.88, 68.73, 67.38, 65.91, 62.35, 42.61, 35.10, 31.01, 20.79, 20.68, 20.64; MS (ESI): 610.35 $[M+Na]^+$, calcd. 610.19 for $C_{26}H_{37}NO_{12}S + Na^+$

Compound 68

Compound **68** was prepared according to general procedure for sulfamide synthesis described above. Colourless oil, 61.4 mg, 73% yield. Rf: 0.22 (MeOH: CH_2Cl_2 5: 95); $[\alpha]^{20}_{D}$ +22.7 (c = 1, $CHCl_3$). ^{1}H NMR (300 MHz, $CDCl_3$): δ 7.17 (2 H, s, Har), 5.29-5.21 (3 H, m, H-4, H-3, H-2), 4.89-4.85 (1 H, m, NH), 4.78 (1 H, d, J = 1.1 Hz, H-1), 4.25 (1 H, dd, J = 12.3, 5.4 Hz, H-6), 4.18-4.06 (3 H, m, H-6, 2 $C\underline{H}(CH_3)_2$), 3.99-3.94 (1 H, m, H-6), 3.89-3.82 (1 H, m, $OC\underline{H_2}CH_2N$), 3.58-3.52 (1 H, m, $OC\underline{H_2}CH_2N$), 3.25-3.13 (2 H, m, $OCH_2C\underline{H_2}N$), 2.94-2.85 (1 H, m, $C\underline{H}(CH_3)_2$), 2.14, 2.09, 2.05, 2.02 (12 H, 4 x s, 4 x OAc), 1.28-1.24 (18 H, s, 3 $CH(CH_3)_2$); ^{13}C NMR (75 MHz, $CDCl_3$): δ 170.60, 169.90, 169.75, 169.62, 152.88, 150.17, 131.91, 123.84, 97.85, 69.09, 68.82, 68.77, 67.34, 65.88, 62.31, 42.33, 34.08, 29.64,

29.57, 24.78, 23.51, 20.78, 20.66, 20.64, 20.59; MS (ESI): 680.44 $[M+Na]^+$, calcd. 680.27 for $C_{31}H_{47}NO_{12}S + Na^+$

Compound 69

Compound **69** was prepared according to general procedure for sulfamide synthesis described above. Colourless oil, 50 mg, 62% yield. Rf: 0.19 (MeOH: CH_2Cl_2 5: 95); $[\alpha]^{20}_D$ +28.7 (c = 1, $CHCl_3$). ^{1}H NMR (300 MHz, $CDCl_3$): δ 8.64 (1 H, d, J = 8.4 Hz, Har), 8.26 (1 H, dd, J = 7.4, 1.1 Hz, Har), 8.07 (1 H, d, J = 8.2 Hz, Har), 7.94 (1 H, d, J = 8.4 Hz, Har), 7.71-7.66 (1 H, m, Har), 7.60-7.52 (2 H, m, Har), 5.45-5.43 (1 H, m, NH), 5.21-5.11 (3 H, m, H-4, H-3, H-2), 4.59 (1 H, bs, H-1), 4.11 (1 H, dd, J = 12.3, 5.3 Hz, H-6), 3.98 (1 H, dd, J = 12.3, 2.2 Hz, H-5), 3.84-3.79 (1 H, m, H-6), 3.61-3.54 (1 H, m, $OC\underline{H}_2CH_2N$), 3.29-3.23 (1 H, m, $OC\underline{H}_2CH_2N$), 3.20-3.14 (2 H, m, $OCH_2C\underline{H}_2N$), 2.13, 2.10, 2.05, 1.99 (12 H, 4 x s, 4 x OAc); ^{13}C NMR (75 MHz, $CDCl_3$): δ 170.59, 169.86, 169.80, 169.60, 134.72, 134.35, 134.17, 129.47, 129.07, 128.52, 127.93, 126.89, 124.159, 97.77, 69.01, 68.82, 68.62, 67.17, 65.78, 62.24, 42.65, 20.77, 20.64, 20.61; MS (ESI): 604.3 $[M+Na]^+$, calcd. 604.1 for $C_{26}H_{31}NO_{12}S + Na^+$

Compound 70

Compound 70 was prepared according to general procedure of de-*O*-acetylation described above. 42 mg, 88% yield. $[\alpha]^{20}_D$ +18.2 (c = 1, CH_3OH).^{1}H NMR (300 MHz, CD_3OD): δ 7.76-7.72 (2H, m, Har), 7.51-7.44 (3H, m, Har), 4.52 (1H, d, J = 1.7 Hz, H-1), 3.73-3.27 (7H, m, H-2,3,4,6_a,6_b and OCH_2), 2.98-2.93 (3H, m, CH_2NH-S); ^{13}C NMR (75 MHz, CD_3OD): δ 133.6, 130.3, 130.2, 127.9, 101.8, 74.7, 72.4, 71.9, 68.5, 67.4, 62.8, 43.9; MS (ESI): 386.30 $[M+Na]^+$, calcd. 386.37 for $C_{14}H_{21}NO_8S + Na^+$

Compound 71

Compound **71** was prepared according to general procedure of de-*O*-acetylation described above. 42 mg, 96% yield. $[\alpha]^{20}_D$ +17.1 (c = 1, CH_3OH).^{1}H NMR (300 MHz, CD_3OD): δ 8.37 (2 H, d, J = 8.5 Hz, Har), 8.04 (2 H, d, J = 8.5 Hz, Har), 4.60 (1 H, bs, H-1), 3.76-3.58 (3 H, m, H-6, H-5, H-2), 3.52-3.28 (3 H, m, H-6, H-4, H-3); ^{13}C NMR (75 MHz, CD_3OD): δ 151.43, 148.71, 129.32, 125.45, 101.76, 74.74, 72.452, 71.88, 68.50, 67.43, 62.82, 43.98; MS (ESI): 431.30 $[M+Na]^+$, calcd. 431.37 for $C_{14}H_{20}N_2O_{10}S + Na^+$

Compound 72

Compound **72** was prepared according to general procedure of de-*O*-acetylation described above. 24.5 mg, 98% yield. $[\alpha]^{20}_D$ +23.6 (c = 1, CH_3OH).^{1}H NMR (300 MHz, CD_3OD): δ 7.73 (2 H, d, J = 8.1 Hz,

Har), 7.37 (2 H, d, J = 8.1 Hz, Har), 4.63 (1 H, d, J = 1.3 Hz, H-1), 3.80-3.73 (2 H, m, H-3, H-2), 3.71-3.54 (4 H, m, H-6, H-5, H-4, $OC\underline{H}_2CH_2N$), 3.47-3.38 (2 H, m, H-6, $OC\underline{H}_2CH_2N$), 3.08-3.03 (2 H, m, $OCH_2C\underline{H}_2N$), 2.42 (3 H, s, $C\underline{H}_3$); ^{13}C NMR (75 MHz, CD_3OD): δ 144.67, 139.10, 130.74, 128.00, 101.75, 74.64, 72.45, 71.89, 68.50, 67.38, 62.78, 43.83, 21.45; MS (ESI): 400.40 $[M+Na]^+$, calcd. 400.40 for $C_{15}H_{23}NO_8S + Na^+$

Compound 73

Compound **73** was prepared according to general procedure of de-*O*-acetylation described above. 24.3 mg, 97% yield. $[\alpha]^{20}_D$ +19.8 (c = 1, CH_3OH).^1H NMR (300 MHz, CD_3OD): δ 7.77 (2 H, d, J = 8.5 Hz, Har), 7.60 (2 H, d, J = 8.5 Hz, Har), 4.64 (1 H, d, J = 1.3 Hz, H-1), 3.81-3.71 (2 H, m, H-3, H-2), 3.69-3.51 (4 H, m, H-6, H-5, H-4, $OC\underline{H}_2CH_2N$), 3.48-3.39 (2 H, m, H-6, $OC\underline{H}_2CH_2N$), 3.11-3.03 (2 H, m, $OCH_2C\underline{H}_2N$), 1.35 (9 H, s, $C(C\underline{H}_3)_3$); ^{13}C NMR (75 MHz, CD_3OD): δ 157.52, 139.03, 127.86, 127.22, 101.77, 74.67, 72.47, 71.92, 68.51, 67.48, 62.80, 43.85, 36.00, 31.48; MS (ESI): 442.40 $[M+Na]^+$, calcd. 442.48 for $C_{18}H_{29}NO_8S + Na^+$

Compound 74

Compound **74** was prepared according to general procedure of de-*O*-acetylation described above. 41.4 mg, 94% yield. $[\alpha]^{20}_D$ +20.6 (c = 1, CH_3OH).^1H NMR (300 MHz, D_2O): δ 7.11 (2H, s, Har), 4.56 (1H, d, =1.7 Hz, H-1), 4..07 (3H, m, CH), 3.71-2.78 (10H, m, H-2, 3, 4, 5, 6a, 6b, OCH_2, CH_2N), 1.13 ppm (18H, d, J =6.9 Hz, CH_3); ^{13}C NMR (75 MHz, CD_3OD): δ 151.7, 134.6, 124.9, 101.6, 74.7, 71.9, 68.5, 67.4, 62.8, 35.4, 30.6, 25.2, 24.0; MS (ESI): 512.40 $[M+Na]^+$, calcd. 512.23 for $C_{23}H_{39}NO_8S + Na^+$

Compound 75

Compound **75** was prepared according to general procedure of de-*O*-acetylation described above. 30 mg, 96% yield. $[\alpha]^{20}_D$ +23.5 (c = 1, CH_3OH).^1H NMR (300 MHz, CD_3OD): δ 9.62-8.60, 8.15-8.13, 8.09-8.07, 7.95-7.94, 7.62-7.52 (7H, m, Har), 5.27 (1H, sl, NH), 4.36, (1H, d, J=1.0 Hz, H-1), 3.47-3.00 ppm (10 H, m, H-2, 3, 4, 5, 6a, 6b, OCH_2 and CH_2N) ; ^{13}C NMR (75 MHz, CD_3OD): δ; 135.2, 130.1, 130.0, 129.0, 128.0, 125.9, 125.3, 101.0, 74.6, 72.4, 71.8, 8.5. 67.3, 62.8, 43.7; MS (ESI): 436.10 $[M+Na]^+$, calcd. 436.10 for $C_{18}H_{23}NO_8S + Na^+$

Compound 76

Compound **76** was prepared as described in the literature: A. Giannis, K. Sandhoff, K. *Tetrahedron Lett.* **1985**, *26,* 1479-1482. ESI$^+$-HRMS: $[M+NH_4]^+$ calcd for $C_{37}H_{44}NO_5$, 582.3214; found, 582.3226.

Compound 77 : full structural characterization

To a solution of allyl-2,3,4,6-tetra-*O*-benzyl-α-mannopyranoside **76** (500 mg, 0.88 mmol) in THF (200 mL) cooled to -78°C was added lithium (10 eq, 62 mg) and NH_3 (100 mL) was condensed until the solution had a persistent blue color (5 h). The reaction mixture was stirred for 3 h and then quenched with ethanol. The mixture was allowed to warm to room temperature and was stirred overnight to allow the complete ammonia evaporation. The remaining solution was concentrated and then the crud product was dissolved in acetic anhydride (10 mL) and pyridine (15 mL) and catalytic DMAP was added. The mixture was stirred overnight and the crude product was extract with CH_2Cl_2 (3 x 20 mL). The crude product was then purified by flash column chromatography using 0-13% AcOEt: hexanes as the eluant, resulting in **77** (310 mg, 94%) as a white solid. Rf: 0.22 (AcOEt/Hexanes 3:7^{1}H NMR (600 MHz, $CDCl_3$): δ 5.77 (1 H, J = 17.4, 10.2, 6.8 Hz, C$\underline{H}$=CH_2), 5.26 (1 H, dd, $J_{2,3}$ = 3.3, $J_{3,4}$ = 8.8 Hz, H-3), 5.20 (1H, dd, $J_{1,2}$ = 3.3 Hz, H-2), 5.19 (1 H, dd, $J_{3,4}$ = $J_{4,5}$ = 8.8 Hz, H-4), 5.18-5.12 (2H, m, CH=C$\underline{H}_2$), 4.32 (1 H, dd, J = 12.1, 6.4 Hz, H-6a), 4.11 (1 H, dd, J = 12.1, 2.9 Hz, H-6b), 4.04 (1 H, ddd, $J_{1,2}$ = 3.3 Hz, $J_{1,CH2A}$ = 15.2 Hz, $J_{1,CH2B}$ = 7.0 Hz, H-1), 3.93-3.86 (1 H, m, H-5), 2.52 (1 H, ddd, $J_{1,CHA}$ = 15.2 Hz, $J_{CHA,CHB}$ = 14.7 Hz, $J_{CHA,CH}$ = 6.4 Hz, C$\underline{H}_2$CH), 2.41 (1 H, ddd, $J_{1,CHB}$ = 7.0 Hz, $J_{CHA,CHB}$ = 14.7 Hz, $J_{CHB,CH}$ = 6.0 Hz, C$\underline{H}_2$CH), 2.12, 2.08, 2.06, 2.02 (12 H, 4 s, OAc); ^{13}C NMR (125 MHz, $CDCl_3$): δ 170.9, 170.4, 170.1, 169.8 (*C*(O)), 132.7 (CH_2*C*HC=CH_2), 118.5 (CH_2CHC=*C*H_2), 74.3 (*C*-1), 70.8 (*C*-5), 70.2 (*C*-4), 70.0 (*C*-3), 67.2 (*C*-2), 62.6 (*C*-6), 33.8, 21.1, 21.0, 20.9 (OAc); MS (ESI): 373.14 $[M+H]^+$, calcd. 373.13 for $C_{17}H_{24}O_9 + H^+$

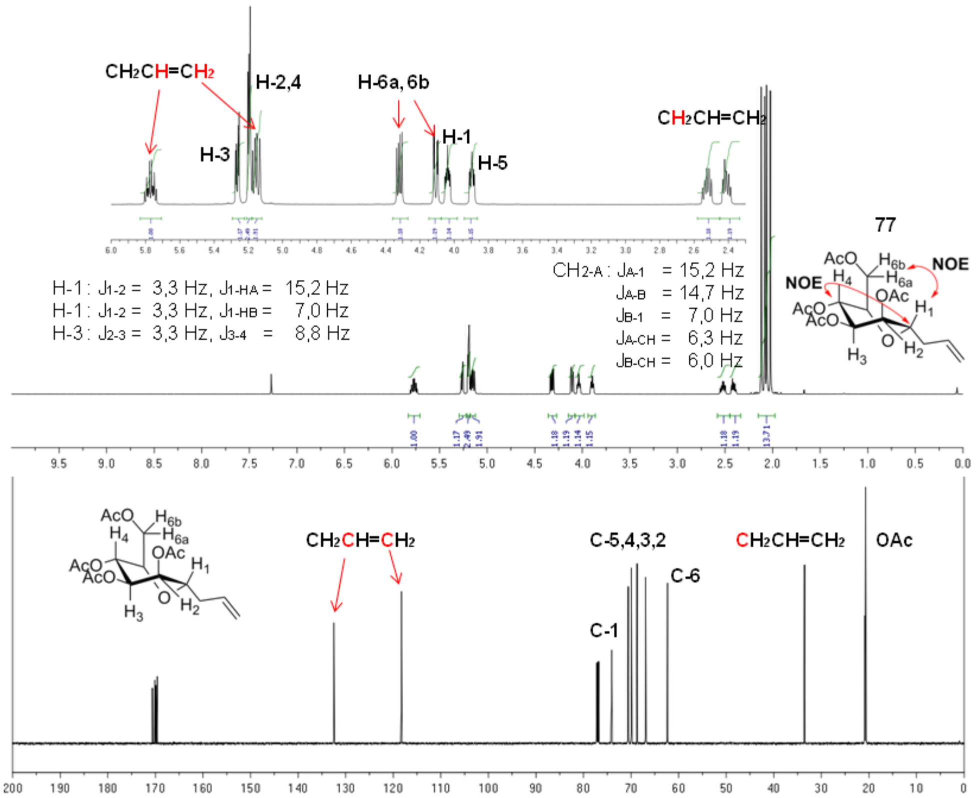

Figure S1. 600 MHz ^{1}H- and ^{13}C-NMR ($CDCl_3$) of **77**.

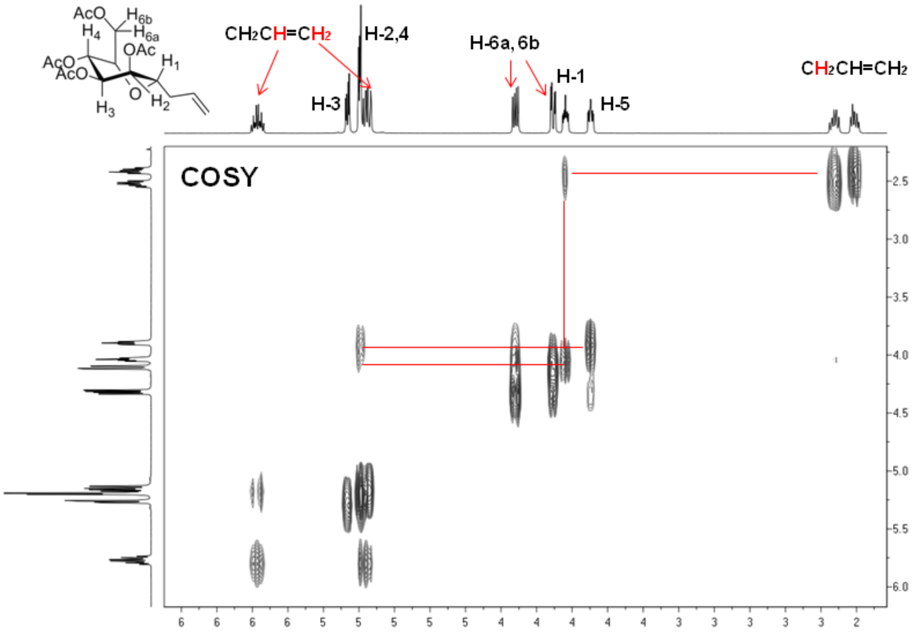

Figure S2. 600 MHz COSY ^{1}H-NMR ($CDCl_3$) of **77** illustrating the protons correlation.

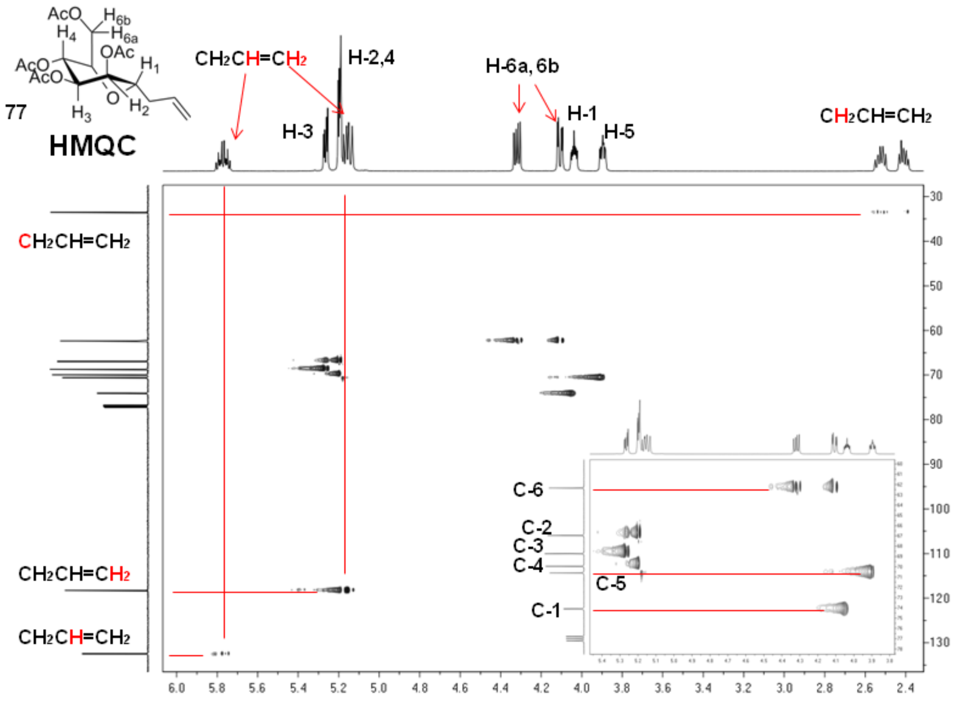

Figure S3. 600 MHz HMQC-NMR ($CDCl_3$) of **77** illustrating the proton-carbon correlation used for the total signal assignments.

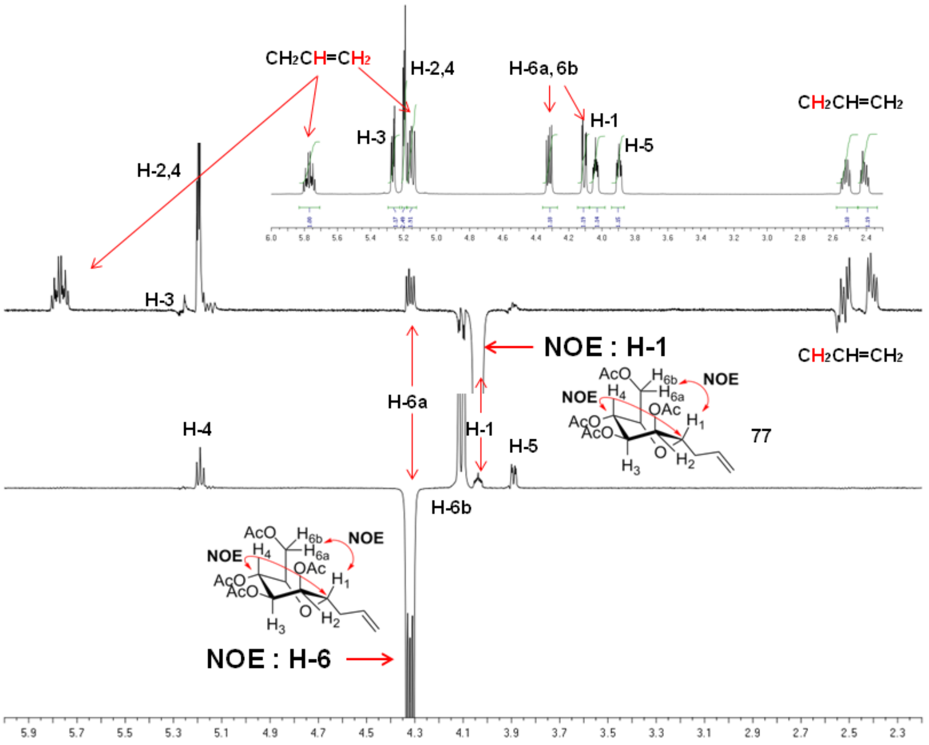

Figure S4. 600 MHz 1D-NOE NMR ($CDCl_3$) of **77** clearly illustrating the connectivity between H1-H6a-H4, thus illustrating that it is in a skewed-boat conformation.

Compound 78

Compound **78** was prepared according to general procedure of de-*O*-acetylation described above. 22 mg, 92% yield. $[\alpha]^{20}_{D}$ +38.2 (c = 1, CH_3OH). ^1H NMR (300 MHz, D_2O): δ 5.75-5.61 (1H, m, C<u>H</u>=CH_2), 5.01 (2 H, m , CH=<u>CH_2</u>), 3.86-3.81 (1 H, m, H-6), 3.76-3.74 (1 H, m, H-5), 3.71-3.64 (2 H, m, H-6, H-1), 3.59-3.39 (3 H, m, H-4, H-3, H-2), 2.44-2.33 (1 H, m, <u>CH_2</u>CH=CH), 2.24-2.16 (1 H, m, <u>CH_2</u>CH=CH); ^{13}C NMR (75 MHz, D_2O): δ 133.69, 117.24, 77.15, 73.28, 70.26, 70.14, 66.80, 60.69, 32.11. ^1H NMR (900 MHz, DMSO-*d*6): δ 5.82 (1H, $J_{b,c}$ = 17.2, $J_{b,c'}$ = 10.2, $J_{a,b}$ = 6.7 Hz, C<u>H</u>=CH_2), 5.08 (1H, dd, $J_{b,c}$ = 17.2, $J_{c,c'}$ = 1.9 Hz, CH=C<u>H_2</u>), 5.01 (1H, dd, $J_{b,c'}$ = 10.2, $J_{c,c'}$ = 1.9, CH=C<u>H_2</u>), 4.69 (1H, d, J = 5.2 Hz, OH-4), 4.59 (1H, d, J = 4.8 Hz, OH-3), 4.42 (1H, d, J = 4.6 Hz, OH-2), 4.33 (1H, t, J = 5.9 Hz, OH-6), 3.68 (1H, ddd, J = 7.8, 6.1, 3.4 Hz, H-1), 3.58 (1H, ddd, $J_{6a,6b}$ = 11.4Hz, $J_{5,6a}$ = 3.3 Hz, $J_{6,OH}$ = 5.9 Hz, H-6a), 3.52 (2H, m, H-2 and H-3), 3.48 (1H, ddd, $J_{6a,6b}$ = 11.4Hz, $J_{5,6b}$ = 5.9 Hz, $J_{6,OH}$ = 5.9 Hz, H-6b), 3.42 (1H, td, $J_{3,4}$ = $J_{4,5}$ = 7.3 Hz, $J_{4,OH}$ = 5.2 Hz, H-4), 3.30 (1H, ddd, $J_{4,5}$ = 7.3 Hz, $J_{5,6b}$ = 5.9 Hz, $J_{5,6a}$ = 3.3 Hz, H-5), 2.27 (2 H, td, J = 7.1, 6.7, 1.9 Hz, C<u>H_2</u>CH=CH); ^{13}C NMR (150 MHz, DMSO-*d*6): δ 136.1 (<u>C</u>H=CH_2), 116.9 (CH=<u>C</u>H_2), 76.4 (C-5), 75.3(C-1), 71.4, 70.1 (C-2 and C-3), 68.6 (C-4), 61.6 (C-6) and 34.4 ppm (<u>C</u>H_2CH=CH). HRMS (ESI): 205.0997 $[M+H]^+$, calcd. 205.1070 for $C_9H_{16}O_5 + H^+$

Full conformational analysis of 78

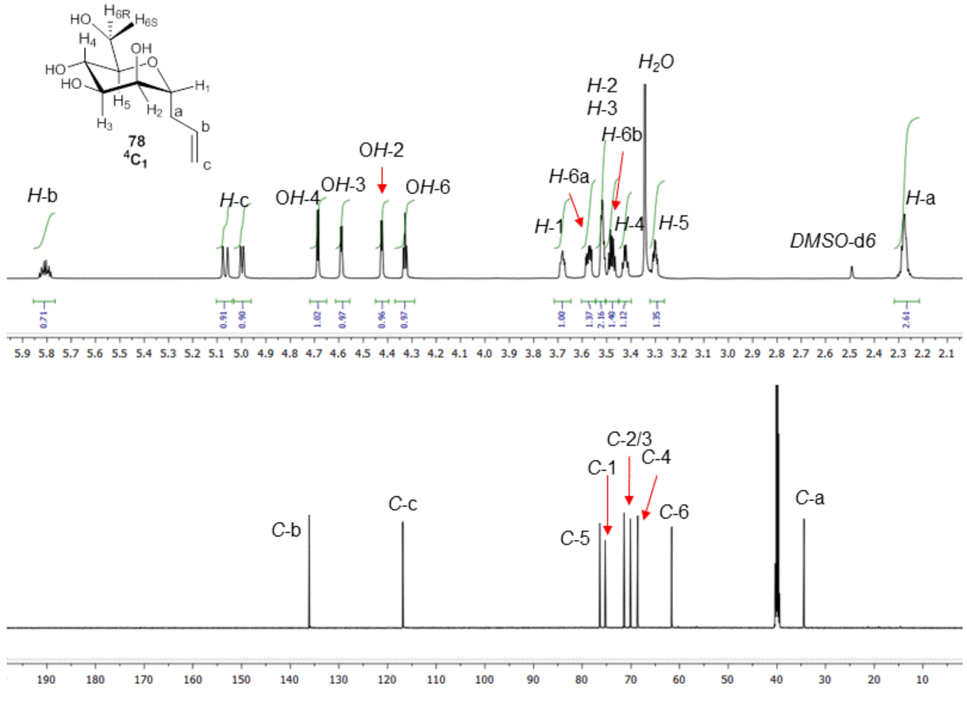

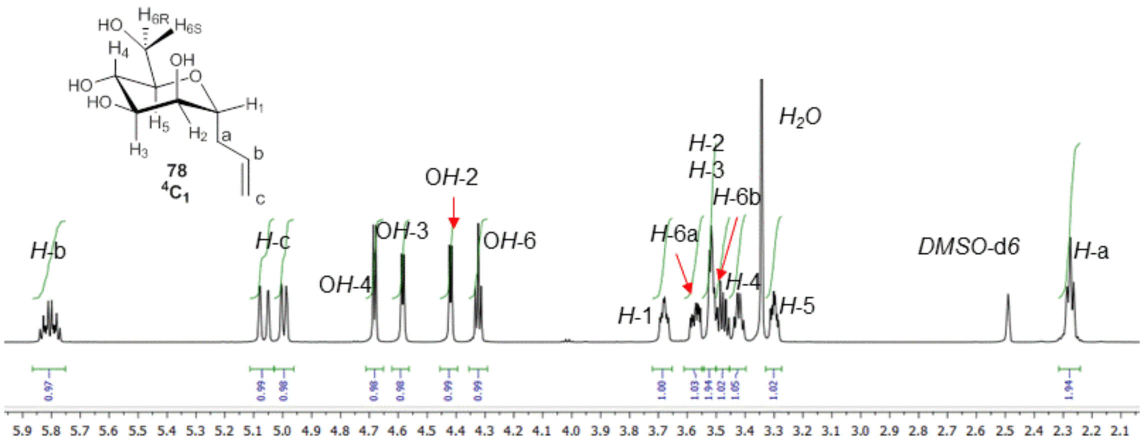

Figure S5. 900 (Top) and 600 (Bottom) MHz ^{1}H and ^{13}C-NMR in DMSO-d6 of **78**.

.

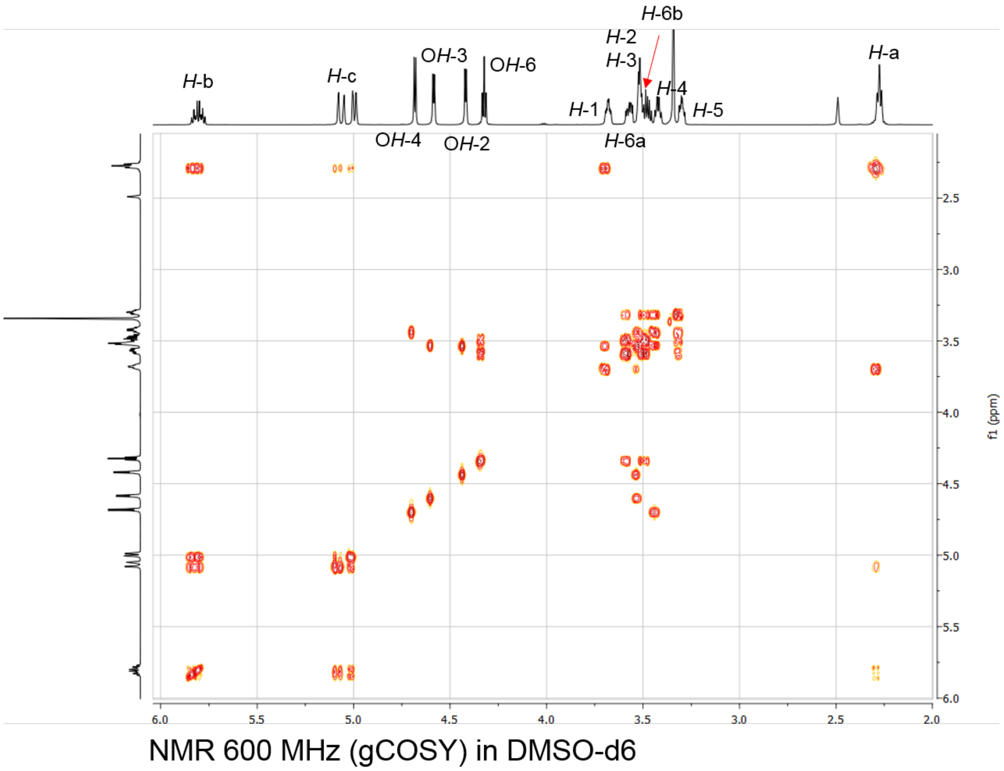

Figure S6. Cpd **78**.

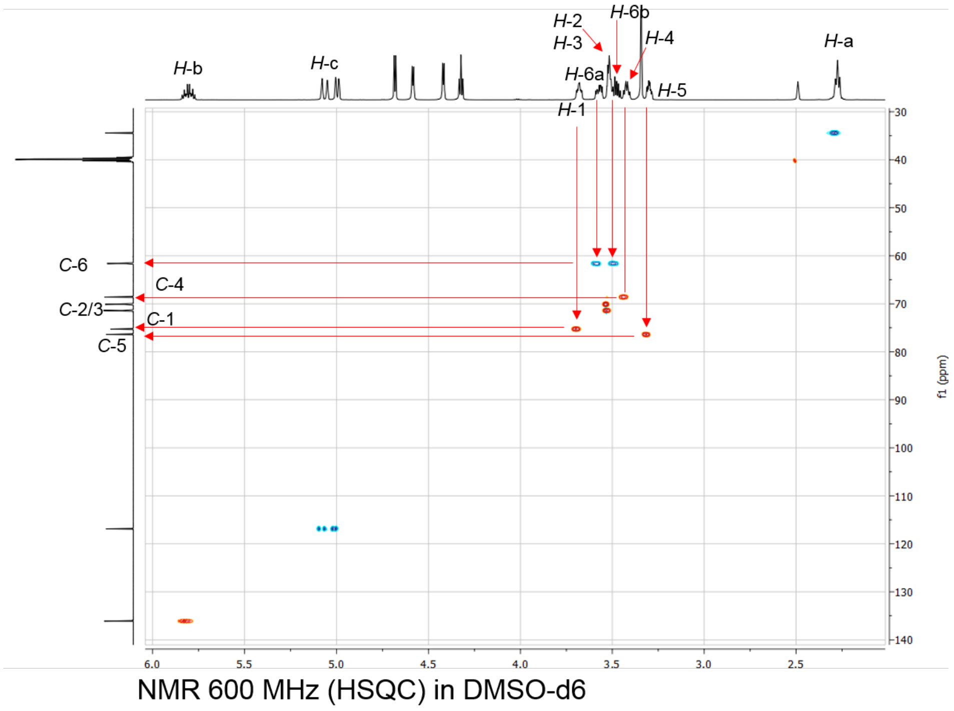

Figure S7. Cpd **78**

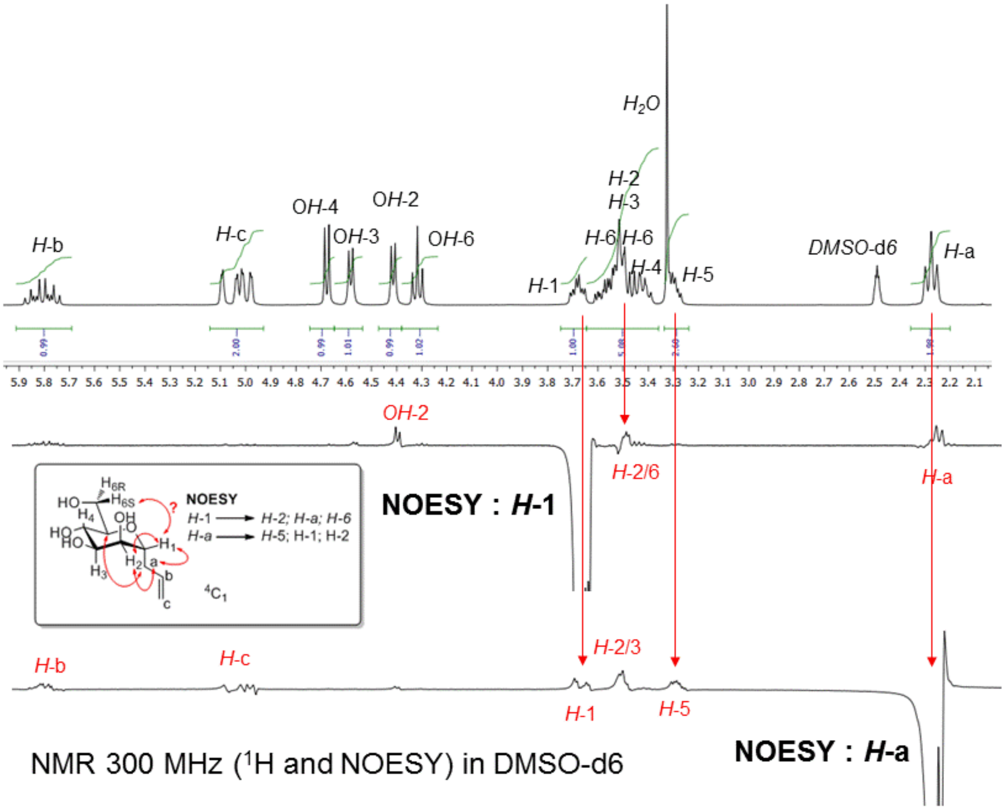

Figure S8.

Compound 79 was prepared according to a published procedure [2].

Compound 80

A solution of allyl 2,3,4,6-tetra-*O*-acetyl-α-mannopyranoside **77** (2.7 g, 7.3 mmol) was dissolved in anhydrous DCM (100 mL) and cooled to -78°C. Ozone was bubbled through the solution until a persistent blue color. The mixture was then deozonized by bubbling nitrogen until it was clear. Acetic acid (6.5 mL) and zinc dust (6.5 g) was added to the solution and the mixture was allowed to warm to room temperature overnight. The reaction mixture was filtered through celite and the washing were concentrated to dryness. The crud aldehyde was used for the next steep without purification. To a solution of a crud 2,3,4,6-tetra-O-acetyl-α-mannopyranosyl aldehyde (1.3 g, 3.5 mmol) in *t*-BuOH (20 mL) and a solution of KH_2PO_4 (1 M, 7 mL) were added to a vigorously stirred solution of $KMnO_4$ (1 M, 7 mL). The reaction mixture was stirred for 30 minutes. A solution of saturated Na_2SO_3 was added until the $KMnO_4$ was neutralized, giving a brown precipitate of MnO_2 which was filtered and washed with EtOH (2 x 20 mL). The EtOH was removed and the remaining solution acidified to pH 3 using 1 N HCl. The acidic solution was then extracted with DCM (3 x 50 mL). The combined extracts were concentrated to dryness resulting in a white foam **80** (1.28 g, 94 %); Rf: 0.41 (Methanol: dichloromethane 1: 9). $[\alpha]^{20}_{D}$ +17.8 (*c* = 1, $CHCl_3$). ^1H NMR (300 MHz, $CDCl_3$): δ 5.25-5.21 (1 H, m, H-3), 5.15-5.09 (2 H, m, H-4, H-2), 5.07-4.39 (2 H, m, H-6, H-1), 4.13 (1 H, dd, J = 12.1, 3.8 Hz, H-6), 4.01-3.95 (1 H, m, H-5), 2.74 (1 H, dd, J = 15.9, 9.6 Hz, $C\underline{H_2}CO$), 2.66 (1 H, dd, J = 15.9, 2.7 Hz, $C\underline{H_2}CO$); ^{13}C NMR (75 MHz, $CDCl_3$): δ 174.19, 170.65, 169.80, 169.61, 169.52, 72.05, 69.32, 69.22, 68.03, 67.41, 61.49, 35.38, 20.63, 20.55. ESI^+-HRMS: $[M+H]^+$ calcd for $C_{16}H_{23}O_{11}$, 391.1235; found, 391.1243.

Compound 81

Compound **81** was prepared according to general procedure described above. Colourless oil, 133.76 mg, 88% yield. Rf: 0.12 (Methanol: dichloromethane 0.5: 9.5); $[\alpha]^{20}_{D}$ +2.87 (*c* = 1, $CHCl_3$). ^1H NMR (300 MHz, $CDCl_3$): δ 8.96 (1 H, m, NH), 7.52-7.46 (2 H, m, Har), 7.33-7.28 (15 H, m, Har), 7.23-7.19 (8 H, m, Har), 4.56-4.44 (8 H, m, 4 $OC\underline{H_2}Ph$), 4.39-4.32 (1 H, m, H-1), 4.22-4.17 (1 H, m, H-5), 4.04-3.98 (1 H, m, H-6), 3.80-3.77 (1 H, m, H-3), 3.64 (1 H, dd, J = 8.2, 3.0 Hz, H-2), 3.59-3.57 (1 H, m, H-4), 3.45 (1 H, dd, J = 3.5, 10.4 Hz, H-6), 2.82-2.65 (2 H, m, $CHC\underline{H_2}$); ^{13}C NMR (75 MHz, $CDCl_3$): δ 168.95, 128.22, 137.70, 137.56, 137.50, 128.63, 128.45, 128.40, 128.37, 128.11, 127.83, 127.73, 123.69, 120.15, 75.41, 74.83, 74.72, 73.62, 73.22, 72.87, 72.51, 71.83, 67.34, 67.11, 38.54; MS (ESI): 658.41 $[M+H]^+$, calcd. 658.32 for $C_{42}H_{43}NO_6 + H^+$

Compound 82

Compound **82** was prepared according to general procedure described above. Colourless oil, 130 mg, 75% yield. Rf: 0.22 (Methanol: dichloromethane 0.5: 9.5); $[\alpha]^{20}_{D}$ +9.4 (*c* = 1, $CHCl_3$). ^{1}H NMR (300 MHz, $CDCl_3$): δ 7.39-7.14 (25 H, m, Har), 4.57-4.47 (8 H, m, 4 OC$\underline{H_2}$Ph), 4.38-4.34 (1 H, m, H-1), 4.31 (2 H, s, NHC$\underline{H_2}$Ph), 4.02-3.85 (1 H, m, H-5), 3.88 (1 H, t, J = 9.1 Hz, H-6), 3.75 (1 H, dd, J = 5.1, 3.0 Hz, H-3), 3.61-3.55 (2 H, m, H-4, H-2), 3.43 (1 H, dd, J = 10.5, 3.4 Hz, H-6), 2.66 (1 H, dd, J = 15.9, 2.7 Hz, C$\underline{H_2}$CO), 2.66 (1 H, dd, J = 15.9, 9.6 Hz, C$\underline{H_2}$CO); ^{13}C NMR (75 MHz, $CDCl_3$): δ 170.56, 138.66, 137.74, 137.69, 137.65, 128.43, 128.39128.33, 128.07, 127.86, 127.82, 127.77, 127.68, 127.41, 126.91, 74.55, 74.26, 74.19, 73.01, 72.67, 71.77, 67.54, 43.03, 37.39; MS (ESI): 694.49 $[M+Na]^+$, calcd. 694.31 for $C_{43}H_{45}NO_6 + Na^+$

Compound 83

Compound **83** was prepared according to general procedure described above. Colourless oil, 108 mg, 91% yield. Rf: 0.28 (Methanol: dichloromethane 0.5: 9.5); $[\alpha]^{20}_{D}$ +24.0 (*c* = 1, $CHCl_3$). ^{1}H NMR (300 MHz, $CDCl_3$): δ 7.34-7.11 (25 H, m, Har), 7.0 8.96 (1 H, m, NH), 4.54-4.46 (8 H, m, 4 OC$\underline{H_2}$Ph), 4.32-4.26 (1 H, m, H-1), 4.08-4.03 (1 H, m, H-5), 3.88 (1 H, t, J = 9.5 Hz, H-6), 3.78-3.57 (1 H, m, H-3), 3.64-3.59 (1 H, m, H-2, H-4), 3.52 (1 H, dd, J = 9.8, 3.1 Hz, H-6), 3.22-3.01 (2 H, m, HNC$\underline{H_2}$), 2.68-2.48 (4 H, m, C$\underline{H_2}$Ph, C$\underline{H_2}$CO), 1.68 (2 H, q, J = 7.8 Hz, HNCH$_2$C$\underline{H_2}$); ^{13}C NMR (75 MHz, $CDCl_3$): δ 170.54, 141.69, 137.78, 137.76, 137.67, 128.43, 128.39, 128.29, 128.29, 128.24, 128.05, 127.93, 127.85, 127.82, 127.78, 127.76, 127.70, 125.71, 75.56, 74.69, 74.43, 74.15, 73.18, 72.75, 72.69, 71.79, 67.88, 67.76, 38.93, 37.29, 33.08, 31.11; MS (ESI): 700.31 $[M+H]^+$, calcd. 699.36 for $C_{45}H_{49}NO_6 + H^+$

Compound 84

Compound **84** was prepared according to general procedure described above. Colourless oil, 256 mg, 75% yield. Rf: 0.25 (Methanol: dichloromethane 0.5: 9.5); $[\alpha]^{20}_{D}$ +11.3 (*c* = 1, $CHCl_3$). ^{1}H NMR (300 MHz, $CDCl_3$): δ 8.92 (1 H, brs, NH), 7.41-7.17 (24 H, m, Har), 4.58-4.27 (8 H, m, OC$\underline{H_2}$Ph), 4.36-4.30 (1 H, m, H-1), 4.16-4.13 (1 H, m, H-5), 3.79 (1 H, t, J = 9.7 Hz, H-6), 3.81-3.77 (1 H, m, H-3), 3.63-3.55 (2 H, m, H-4, H-2), 3.42 (1 H, dd, J = 9.8, 36 Hz, H-6), 2.73 (1 H, dd, J = 15.3, 3.1 Hz, C$\underline{H_2}$CO), 2.66 (1 H, dd, J = 15.3, 9.5 Hz, C$\underline{H_2}$CO); ^{13}C NMR (75 MHz, $CDCl_3$): δ 169.09, 168.20, 137.91, 137.74, 137.70, 134.62, 134.03, 129.63, 128.50, 128.46, 128.43, 128.37, 128.22, 128.13, 128.06, 127.95, 127.91, 127.89, 127.86, 127.79, 127.56, 121.03, 120.43, 77.21, 75.76, 75.24, 75.03, 74.93, 74.04, 73.50, 73.36, 73.06, 72.59, 71.97, 67.64, 67.33, 38.68, 24.71; MS (ESI): 715.51 $[M+H]^+$, calcd. 715.34 for $C_{44}H_{46}N_2O_7 + H^+$

Compound 85

Compound **85** was prepared according to general procedure described above. Colourless oil, 263 mg, 76% yield. Rf: 0.15 (Methanol: dichloromethane 0.5: 9.5); $[\alpha]^{20}_{D}$ +27.2 (*c* = 1, $CHCl_3$). ^1H NMR (300 MHz, $CDCl_3$): δ 8.84 (1 H, m, NH), 7.34-7.19 (22 H, m, Har), 6.63 (2 H, d, J = 9.1 Hz, Har), 4.56-4.38 (8 H, m, 4 OC$\underline{H_2}$Ph), 4.39-4.33 (1 H, m, H-1), 4.17-4.14 (1 H, m, H-5), 4.0 (1 H, t, J = 9.7 Hz, H-6), 3.75-3.72 (1 H, m, H-3), 3.65 (1 H, dd, J = 7.7, 2.5 Hz, H-2), 3.59-3.56 (1 H, m, H-4), 3.44 (1 H, dd, J = 10.4, 3.3 Hz, H-6), 2.75 (1 H, dd, J = 14.1, 2.5 Hz, C$\underline{H_2}$CO), 2.68 (1 H, dd, J = 14.1, 9.1 Hz, C$\underline{H_2}$CO); ^{13}C NMR (75 MHz, $CDCl_3$): δ 169.13, 152.67, 137.67, 130.94, 128.49, 128.45, 128.40, 128.16, 127.88, 127.79, 122.51, 115.51, 75.64, 75.02, 74.80, 74.09, 73.34, 73.01, 72.62, 71.98, 67.65, 67.42, 38.35; MS (ESI): 696.38 $[M+Na]^+$, calcd. 696.29 for $C_{42}H_{43}NO_7 + Na^+$

Compound 86

Compound **86** was prepared according to general procedure described above. White solid, m.p. 125-127°C, 246 mg, 88% yield. Rf: 0.18 (Methanol: dichloromethane 0.5: 9.5); $[\alpha]^{20}_{D}$ +19.1 (*c* = 1, $CHCl_3$). ^1H NMR (300 MHz, $CDCl_3$): δ 7.40-7.21 (20 H, m, Har), 7.12 (1 H, m, NH), 6.91 (2 H, d, J = 8.5 Hz, Har), 6.72 (2 H, d, J = 8.5 Hz, Har), 4.57-4.44 (8 H, m, 4 OC$\underline{H_2}$Ph), 4.33-4.26 (1 H, m, H-1), 4.05-4.01 (1 H, m, H-5), 3.92 (1 H, t, J = 9.4 Hz, H-6), 3.78-3.75 (1 H, m, H-3), 3.65-3.58 (2 H, m, H-4, H-2), 3.44 (1 H, dd, J = 10.4, 3.4 Hz, H-6), 3.37-3.26 (2 H, m, HNC$\underline{H_2}$), 2.65-2.47 (4 H, m, C$\underline{H_2}$CO, C$\underline{H_2}$Ph); ^{13}C NMR (75 MHz, $CDCl_3$): δ 171.13, 154.95, 137.78, 137.71, 137.67, 137.61, 130.01, 129.62, 128.41, 128.37, 128.22, 128.05, 127.92, 127.83, 127.79, 127.68, 127.55, 115.50, 115.34, 75.34, 74.58, 74.45, 74.05, 73.32, 73.14, 72.77, 72.61, 71.69, 67.82, 67.71, 40.97, 37.10, 34.64; MS (ESI): 702.59 $[M+H]^+$, calcd. 702.34 for $C_{44}H_{47}NO_7 + H^+$

Compound 87

Compound **87** was prepared according to general procedure described for **81-86** amides. Colourless oil, 108 mg, 78% yield. Rf: 0.12 (Methanol: dichloromethane 0.5: 9.5); $[\alpha]^{20}_{D}$ +32.80 (*c* = 1, $CHCl_3$). ^1H NMR (300 MHz, $CDCl_3$): δ 7.82 (2 H, t, J = 9.1 Hz, Har), 7.48-7.45 (1 H, m, Har), 7.35-7.30 (1 H, m, Har), 5.28-3.24 (1 H, m, H-3), 5.18 (1 H, dd, J = 7.4, 3.3 Hz, H-2), 5.03-4.99 (1 H, m, H-4), 4.61-4.49 (2 H, m, H-6, H-1), 4.18-4.06 (2 H, m, H-6, H-5), 2.83-2.81 (2 H, m, C$\underline{H_2}$CO), 2.11, 2.09, 2.05, 2.02 (12 H, 4 s, 4 OAc); ^{13}C NMR (75 MHz, $CDCl_3$): δ 170.82, 169.61, 169.41, 169.26, 167.79, 158.17, 147.99, 132.05, 126.36, 124.08, 121.51, 120.68, 73.11, 68.51, 67.75, 67.30, 67.10, 60.60, 37.67, 20.75, 20.63, 20.60; MS (ESI): 523.12 $[M+H]^+$, calcd. 523.13 for $C_{23}H_{26}N_2O_{10}S + H^+$

Compound 88

Compound **88** was prepared according to general procedure described for **81-86** amides. Colourless oil, 192.7 mg, 68% yield. Rf: 0.15 (Methanol: dichloromethane 0.5: 9.5); $[\alpha]^{20}_{D}$ +11.68 (*c* = 1, $CHCl_3$). ^{1}H NMR (300 MHz, $CDCl_3$): δ 8.41 (1 H, brs, NH), 7.57 (1 H, d, J = 7.7 Hz, Har), 7.35 (1 H, d, J = 7.4 Hz, Har), 7.21-7.16 (1 H, m, Har), 7.13-7.07 (1 H, m, Har), 7.03 (1 H, d, J = 2.4 Hz, C$\underline{H}$=C), 6.11 (1 H, brs, HN), 5.22 (1 H, dd, J = 6.8, 3.1 Hz, H-3), 5.10-5.05 (2 H, m, H-4, H-2), 4.39-4.33 (1 H, m, H-1), 4.26 (1 H, dd, J = 12.1, 6.7 Hz, H-6), 4.09 (1 H, dd, J = 12.1, 4.2 Hz, H-6), 3.89-3.83 (1 H, m, H-5), 3.63-3.56 (2 H, m, HNC$\underline{H_2}$), 2.97 (2H, t, J = 6.7 Hz, HNCH_2C$\underline{H_2}$), 2.51-2.27 (2 H, m, C$\underline{H_2}$CO), 2.06, 2.05, 2.04, 2.02 (12 H, 4 s, 4 OAc); ^{13}C NMR (75 MHz, $CDCl_3$): δ 170.62, 169.87, 169.62, 169.46, 168.73, 136.35, 127.31, 122.10, 122.05, 119.33, 118.55, 112.70, 111.27, 71.77, 69.59, 69.17, 67.84, 67.34, 61.52, 39.93, 37.18, 25.07, 20.70, 20.64; MS (ESI): 555.32 $[M+Na]^+$, calcd. 555.19 for $C_{26}H_{32}N_2O_{10}$ + Na^+

Compound 89 : full structural characterization

Compound **89** was prepared according to general procedure described for **81-86** amides. White solid, 170 mg, 72% yield. Rf: 0.23 (Methanol: dichloromethane 0.4:9.6); $[\alpha]^{20}_{D}$ +15.48 (*c* = 1, $CHCl_3$ ^{1}H NMR (600 MHz, $CDCl_3$): δ 7.54 (2 H, sb, NH, H_{ar}), 7.29 (3 H, m, H_{ar}), 5.27 (1 H, dd, $J_{1,2}$ = 5.4 Hz, $J_{2,3}$ = 3.3 Hz, H-2), 5.19 (1 H, dd, $J_{3,4}$ = 7.0, Hz, H-3), 5.08 (1 H, t, $J_{3,4}$ = $J_{4,5}$ = 7.0 Hz, H-4), 4.58 (1 H, ddd, $J_{1,CH2A}$ = 8.6 Hz, $J_{1,2}$ = 5.4 Hz, $J_{1,CH2B}$ = 5.2 Hz, H-1), 4.40 (1 H, dd, J = 12.0, 6.9 Hz, H-6a), 4.10 (1 H, dd, J = 12.1, 4.0 Hz, H-6b), 3.88 (1 H, dd, J = 10.3, 6.2 Hz, H-5), 2.98 (1 H, dd, $J_{CH2A,CH2B}$ = 14.7 Hz, $J_{CH2A,1}$ = 8.6 Hz, C$\underline{H_{2A}}$CO), 2.90 (1 H, dd, $J_{CH2A,CH2B}$ = 14.7 Hz, $J_{CH2B,1}$ = 5.2 Hz, C$\underline{H_{2A}}$CO), 2.04, (9 H, s, 3 OAc), 1.95 (3H, s, OAc); ^{13}C NMR (125 MHz, $CDCl_3$): δ 170.5, 170.0, 169.7, 169.4, 169.4 (CO), 147.8, 136.32, 129.33, 128.90, 122.6, 72.66, 69.3, 68.9, 67.8, 67.2, 61.3, 37.6, 20.7, 20.7, 20.6, 20.5. ESI^+-HRMS: $[M+H]^+$ calcd for $C_{23}H_{28}N_3O_{10}$, 506.1769; found, 506.1787.

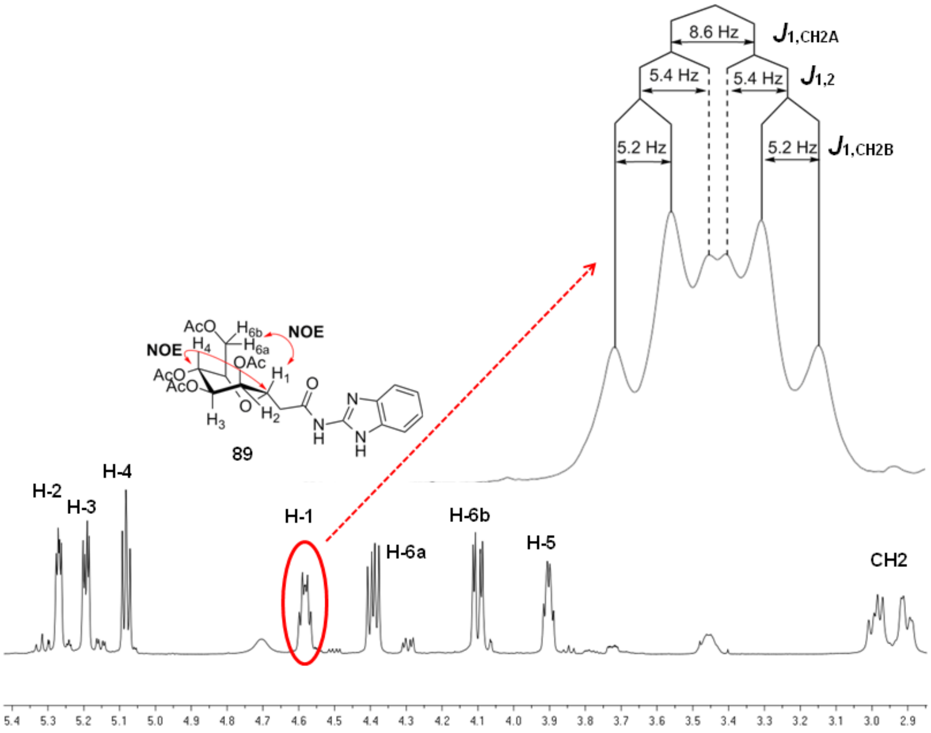

Figure S9. 600 MHz ^{1}H-NMR ($CDCl_3$) of **89** illustrating the complete signal analysis of H1 and its connectivity (NOE) to H6a and H4, hence a skewed-boat conformation.

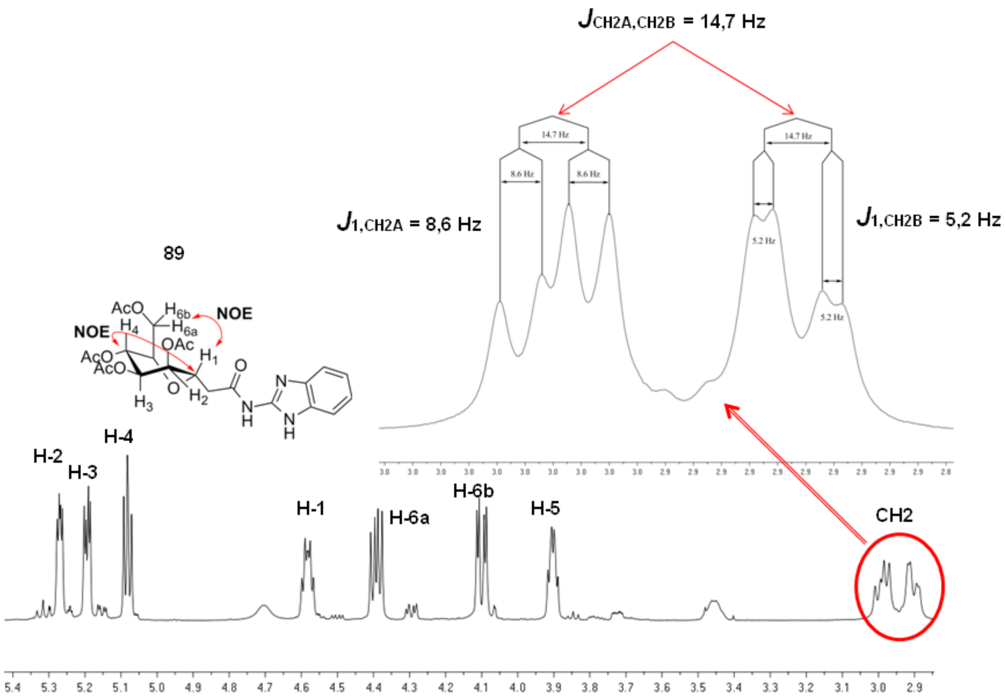

Figure S10. 600 MHz ^{1}H-NMR ($CDCl_3$) of **89** with inset showing the exo-methylene HAB system.

Compound 90

Compound **90** was prepared according to general procedure described for **81-86** amides. Colourless oil, 91 mg, 69% yield. Rf: 0.18 (Methanol: dichloromethane 0.5: 9.5); $[\alpha]^{20}_{D}$ +2.87 (*c* = 1, $CHCl_3$). ^1H NMR (300 MHz, $CDCl_3$): δ 7.53-7.48 (2 H, m, Har), 7.29-7.24 (2 H, m, Har), 5.31-3.25 (1 H, m, H-3), 5.18 (1 H, dd, J = 6.8, 3.1 Hz, H-2), 5.07 (1 H, t, J = 6.1 Hz, H-4), 4.61-4.54 (1 H, m, H-1), 4.41 (1 H, dd, J = 12.1, 6.8 Hz, H-6), 4.11 (1 H, dd, J = 12.1, 4.1 Hz, H-6), 3.91-3.85 (1 H, m, H-5), 3.01-2.86 (2 H, m, CH_2CO), 2.04, 2.0, 1.97, 1.95 (12 H, 4 s, 4 OAc). ^{13}C NMR (75 MHz, $CDCl_3$): δ170.54, 170.01, 169.69, 169.46, 169.42, 147.85, 122.67, 72.29, 69.26, 68.92, 67.83, 67.20, 61.27, 37.62, 20.74, 20.69, 20.60, 20.52. MS (ESI): 506.16 $[M+H]^+$, calcd. 506.17 for $C_{23}H_{27}N_3O_{10} + H^+$

Compound 91

Compound **91** was prepared according to general procedure of debenzylation described above. Colourless oil, 30 mg, 83% yield. $[\alpha]^{20}_{D}$ +43.69 (*c* = 1, CH_3OH). ^1H NMR (300 MHz, CD_3OD): δ 7.56 (2 H, d, J = 7.9 Hz, Har), 7.28 (2 H, t, J = 7.9 Hz, Har), 7.07 (1 H, t, J = 7.3 Hz, Har), 4.41-4.35 (1 H, m, H-1), 3.87 (1 H, dd, J = 12.1, 6.6 Hz, H-6), 3.79-3.60 (5 H, m, H-6, H-5, H-4, H-3, H-2), 2.78 (1 H, dd, J = 14.8, 8.9 Hz, CH_2CO), 2.66 (1 H, dd, J = 14.8, 5.1 Hz, CH_2CO); ^{13}C NMR (75 MHz, CD_3OD): δ 171.62, 139.67, 129.74, 125.29, 121.47, 121.32, 77.63, 73.89, 72.47, 71.70, 69.63, 62.19, 38.54; MS (ESI): 320.10 $[M+Na]^+$, calcd. 320.12 for $C_{14}H_{18}NO_6 + H^+$

Compound 92

Compound **92** was prepared according to general procedure of debenzylation described above. Colourless oil, 16 mg, 88% yield. $[\alpha]^{20}_{D}$ +21.43 (*c* = 1, CH_3OH). ^1H NMR (300 MHz, CD_3OD): δ 7.38-7.23 (5 H, m, Har), 4.88-4.62 (3 H, m, H-1, CH_2Ph), 3.82 (1 H, dd, J = 11.8, 6.3 Hz, H-6), 3.74-3.62 (5 H, m, H-6, H-5, H-4, H-3, H-2), 2.88 (1 H, dd, J = 14.8, 9.1 Hz, CH_2CO), 2.72 (1 H, dd, J = 14.8, 5.1 Hz, CH_2CO); ^{13}C NMR (75 MHz, CD_3OD): δ - 173.20, 139.85, 129.52, 128.55, 128.18, 77.36, 74.21, 72.47, 71.82, 69.54, 62.23, 44.16, 37.59, 31.97, 27.00, 23.76; HRMS; MS (ESI): 334.13 $[M+Na]^+$, calcd. 334.14 for $C_{15}H_{21}NO_6 + Na^+$

Compound 93

Compound **93** was prepared according to general procedure of debenzylation described above. Colourless oil, 33 mg, 86% yield. $[\alpha]^{20}_{D}$ +39.6 (*c* = 1, CH_3OH). ^1H NMR (300 MHz, CD_3OD): δ 7.25-7.20 (2 H, m, Har), 7.15-7.09 (3 H, m, Har), 4.20-4.15 (1 H, m, H-1), 3.74-3.44 (5 H, m, H-6, H-5, H-4, H-3, H-2),

3.47-3.39 (1 H, m, H-6), 3.06 (2 H, t, J = 6.8 Hz, $HNCH_2$), 2.61-2.48 (3 H, m, $HC\underline{H}CO$, $C\underline{H_2}Ph$), 2.27 (1 H, dd, J = 15.1, 4.6 Hz, $\underline{H}CHCO$), 1.68 (2 H, q, J = 7.1 Hz, $HNCH_2C\underline{H_2}$); ^{13}C NMR (75 MHz, CD_3OD): δ 163.02, 132.70, 119.31, 119.19, 116.75, 65.71, 65.16, 61.47, 61.21, 57.77, 51.59, 29.69, 26.06, 23.06, 20.63; MS (ESI): 340.17 $[M+H]^+$, calcd. 340.17 for $C_{17}H_{25}NO_6 + H^+$

Compound 94

Compound **94** was prepared according to general procedure of debenzylation described above. Colourless oil. 95 mg, 83% yield. $[\alpha]^{20}_D$ +28.4 (*c* = 1, CH_3OH). ^1H NMR (300 MHz, CD_3OD): δ 7.46-7.39 (4 H, m, Har), 4.39-4.36 (1 H, m, H-1), 3.83-3.81 (3 H, m, 2 H6, H5), 3.76-3.70 (3 H, m, H-4, H-3, H-2), 2.83 (1 H, dd, J = 14.8, 9.6 Hz, $C\underline{H_2}CO$), 2.61 (1 H, dd, J = 14.8, 4.9 Hz, $C\underline{H_2}CO$), 2.09 (3 H, s, $OCC\underline{H_3}$). ^{13}C NMR (75 MHz, CD_3OD): δ 172.30, 171.62, 135.86, 135.42, 122.26, 121.98, 76.66, 75.09, 72.16, 71.93, 68.94, 62.13, 37.86, 23.78; MS (ESI): 355.15 $[M+H]^+$, calcd. 355.15 for $C_{16}H_{22}N_2O_7 + H^+$

Compound 95

Compound **95** was prepared according to general procedure of debenzylation described above. Colourless oil, 43 mg, 91% yield. $[\alpha]^{20}_D$ +32.8 (*c* = 1, CH_3OH). ^1H NMR (300 MHz, CD_3OD): δ 7.33 (2 H, d, J = 8.8 Hz, Har), 6.73 (2 H, d, J = 8.8 Hz, Har), 4.39-4.33 (1 H, m, H-1), 3.86 (1 H, dd, J = 12.1, 6.8 Hz, H-6), 3.78-3.59 (5 H, m, H-6, H-5, H-4, H-3, H-2), 2.75 (1 H, dd, J = 14.8, 9.1 Hz, $C\underline{H_2}CO$), 2.65 (1 H, dd, J = 14.8, 4.9 Hz, $C\underline{H_2}CO$); ^{13}C NMR (75 MHz, CD_3OD): δ - 171.33, 155.48, 131.48, 123.62, 116.17, 77.52, 74.06, 72.48, 71.75, 69.61, 62.25, 38.30; MS (ESI): 314.12 $[M+H]^+$, calcd. 314.12 for $C_{14}H_{19}NO_7 + H^+$

Compound 96

Compound **96** was prepared according to general procedure of debenzylation described above. Colourless oil, 88 mg, 83% yield. $[\alpha]^{20}_D$ +22.6 (*c* = 1, CH_3OH). ^1H NMR (300 MHz, CD_3OD): δ 7.01 (2 H, dd, J = 6.3, 1.9 Hz, Har), 6.69 (2 H, dd, J = 6.3, 1.9 Hz, Har), 4.29-4.23 (1 H, m, H-1), 3.82 (1 H, dd, J = 11.8, 6.6 Hz, H-6), 3.78-3.59 (4 H, m, H-5, H-4, H-3, H-2), 3.56-3.51 (1 H, m, H-6), 3.34 (2 H, t, J = 7.6 Hz, $HNC\underline{H_2}CH_2$), 2.68 (2 H, t, J = 7.6 Hz, $HNCH_2C\underline{H_2}$), 2.56 (1 H, dd, J = 14.8, 9.0 Hz, $C\underline{H_2}CO$), 2.44 (1 H, dd, J = 14.8, 5.2 Hz, $C\underline{H_2}CO$); ^{13}C NMR (75 MHz, CD_3OD): δ 173.14, 156.87, 131.26, 130.71, 116.25, 77.25, 74.18, 72.41, 71.78, 69.49, 62.20, 42.43, 37.44, 35.63; MS (ESI): 365.40 $[M+Na]^+$, calcd. 365.14 for $C_{16}H_{23}NO_7 + Na^+$

Compound 97

Compound **97** was prepared according to general procedure of de-*O*-acetylation described above. 91 mg, 91% yield. $[\alpha]^{20}_{D}$ +19.8 (c = 1, CH_3OH). ^1H NMR (300 MHz, CD_3OD): δ 7.56 (2 H, d, J = 8.5 Hz, Har), 7.29 (2 H, d, J = 8.5 Hz, Har), 4.48 (1 H, br s, NH), 4.39-4.34 (1 H, m, H-1), 3.88 (1 H, dd, J = 11.8, 6.9 Hz, H-6), 3.77-3.62 (5 H, m, H-6, H-5, H-4, H-3, H-2), 2.76 (1 H, dd, J = 14.8, 9.0 Hz, C$\underline{H_2}$CO), 2.69 (1 H, dd, J = 15.9, 5.9 Hz, C$\underline{H_2}$CO); ^{13}C NMR (75 MHz, CD_3OD): δ 171.67, 138.59, 130.06, 129.71, 122.72, 122.58, 77.80, 73.55, 72.46, 71.59, 69.71, 62.14, 38.63; MS (ESI): 354.40 $[M+Na]^+$, calcd. 354.07 for $C_{14}H_{18}ClNO_6 + Na^+$

Compound 98

Compound **98** was prepared according to general procedure of de-*O*-acetylation described above. 68 mg, 91% yield. $[\alpha]^{20}_{D}$ +23.8 (c = 1, CH_3OH). ^1H NMR (600 MHz**,** DMSO-*d*6): δ 12.3 (1H, sb, NH), 7.95 (1H, d, J = 7.6 Hz, Har), 7.71 (1H, d, J = 7.6 Hz, Har), 7.41 (1H, ddd, J = 8.2, 7.2, 1.3 Hz, Har), 7.28 (1H, ddd, J = 8.2, 7.3, 1.2 Hz, Har), 4.83 (1H, d, 4.4 Hz, OH-4), 4.72 (1H, d, J = 4.5 Hz, OH-2), 4.63 (1H, sb, OH-3), 4.23 (1H, t, J = 5.8 Hz, OH-6), 4.16 (1H, m, H-1), 3.52 (4H, m, H-2, H-3, H-6[a] and H-6b), 3.46 (1H, td, J = 6.4, 4.7 Hz, H-4), 3.36 (1H, td, J = 6.0, 4.3 Hz, H-5) and 2.72 ppm (2H, m, C$\underline{H_2}$CO); ^{13}C NMR (150 MHz, DMSO-*d*6): δ 170.8 (CO), 158.2 ($C_{q\text{-}arom}$), 149.0 ($C_{q\text{-}arom}$), 131.9 ($C_{q\text{-}arom}$), 126.5, 124.0, 122.2, 121.0 (4 x CH-arom), 77.3 (C-5), 71.9 (C-1), 71.4, 70.0, 68.5 (C-2, C-3, C-4), 61.1 (C-6) and 37.4 ppm (CH_2). MS (ESI): 355.10 $[M+H]^+$, calcd. 355.10 for $C_{15}H_{18}N_2O_6S + H^+$.

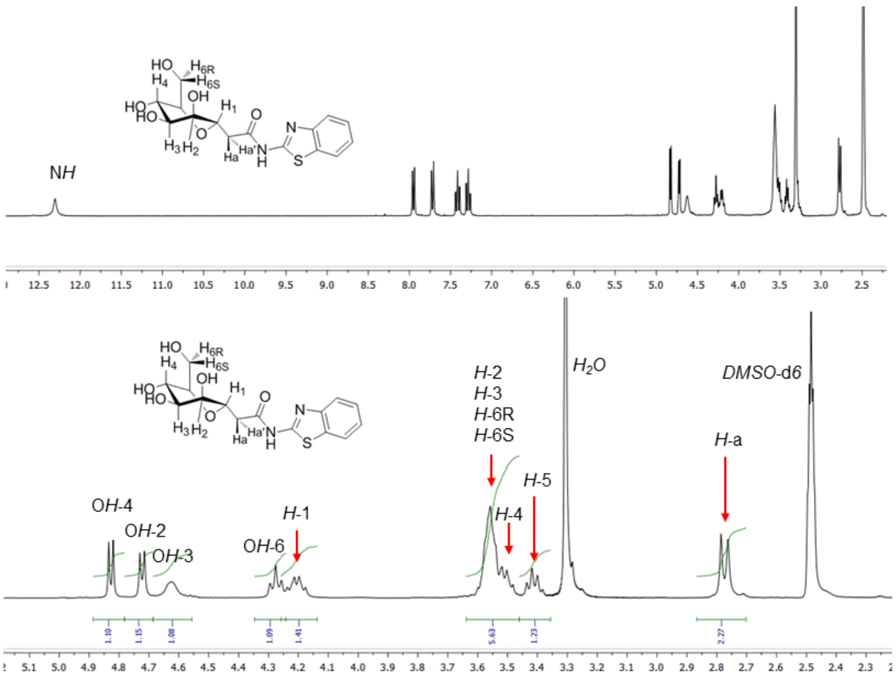

Figure S11. 300 MHz ^1H-NMR (DMSO-d6) of Cpd **98**.

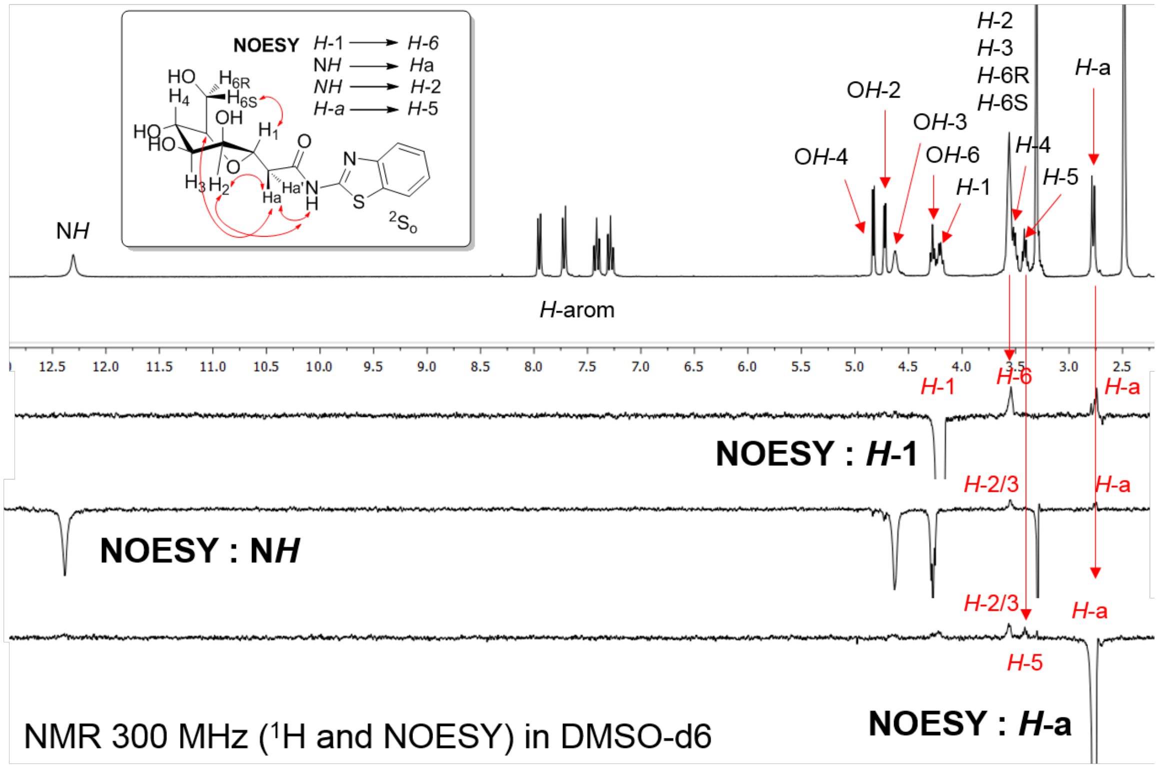

Figure S12. Cpd **98** NOESY spectrum.

Compound 99

Compound **99** was prepared according to general procedure of de-*O*-acetylation described above. 43 mg, 90% yield. $[\alpha]^{20}_{D}$ +17.6 (*c* = 1, CH_3OH). ^{1}H NMR (300 MHz,): δ 7.61-7.52 (2 H, m, Har), 7.27-7.24 (2 H, m, Har), 4.46-4.44 (1 H, m, H-1), 4.33-4.27 (1 H, m, H-6), 3.85-3.73 (4 H, m, H-6. H-5, H-4, H-3), 3.58-3.49 (1 H, m, H-2), 2.94 (1 H, dd, J = 14.8, 9.1 Hz, C$\underline{H_2}$CO), 2.84 (1 H, dd, J = 14.8, 5.4 Hz, C$\underline{H_2}$CO); ^{13}C NMR (75 MHz,): δ 173.71, 147.16, 136.02, 123.78, 115.14, 77.26, 76.95, 74.26, 72.11, 71.48, 69.03, 62.11, 52.83, 37.18. ESI^{+}-HRMS: $[M+H]^{+}$ calcd for $C_{15}H_{21}N_4O_5$, 338.1347; found, 338.1352. MS (ESI): 338.13 $[M+H]^{+}$, calcd. 338.14 for $C_{15}H_{19}N_3O_6 + H^{+}$

Compound 100

Compound **100** was prepared according to general procedure of de-*O*-acetylation described above. 86 mg, 78% yield. $[\alpha]^{20}_{D}$ +22.6 (*c* = 1, CH_3OH). ^{1}H NMR (300 MHz, CD_3OD): δ 8.09 (1 H, brs, NH), 7.56 (1 H, d, J = 7.4 Hz, Har), 7.29 (1 H, d, J = 8.2 Hz, Har), 7.09-7.07 (1 H, m, C$\underline{H}$=C), 7.04-6.96 (1 H, m, Har), 4.28-4.25 (1 H, m, H-1), 3.83 (1 H, dd, J = 11.8, 6.9 Hz, H-6), 3.71-3.65 (4 H, m, H-6, H-5, H-4, H-3), 3.55-3.48 (3 H, m, HNC$\underline{H_2}$CH$_2$, H-2), 2.94 (3 H, t, J = 7.1 Hz, HNCH$_2$C$\underline{H_2}$), 2.57 (1 H, dd, J = 14.8, 9.1 Hz, C$\underline{H_2}$CO), 2.84 (1 H, dd, J = 14.8, 4.9 Hz, C$\underline{H_2}$CO); ^{13}C NMR (75 MHz, CD_3OD): δ 173.21, 138.16,

128.76, 123.46, 122.30, 119.58, 119.26, 113.22, 112.22, 81.97, 77.36, 74.14, 72.46, 71.77, 69.55, 62.28, 41.39, 37.60, 26.22; MS (ESI): 365.40 $[M+H]^+$, calcd. 365.40 for $C_{18}H_{24}N_2O_6 + H^+$

Compound 101

Compound **101** was prepared according to general procedure described above. Colourless oil, 115.6 mg, 83% yield. Rf: 0.19 (AcOEt: Hexanes 3: 7); $[\alpha]^{20}_D$ +18.5 (*c* = 1, $CHCl_3$). ^1H NMR (300 MHz,): δ 7.25-7.16 (5 H, m, Har), 5.18-5.14 (2 H, m, H-3, H-4), 5.11-5.08 (1 H, m, H-2), 4.26 (1 H, dd, J = 12.1, 6.3 Hz, H-6), 4.02 (1 H, dd, J = 12.1, 3.0 Hz, H-5), 3.89-3.84 (1 H, m, H-1), 3.80-3.75 (1 H, m, H-6), 3.65 (2 H, s, $C\underline{H_2}Ph$), 2.46-2.35 (2 H, m, $CH_2CH_2C\underline{H_2}S$), 2.08, 2.04, 2.01, 1.97 (12 H, 4 s, OAc), 1.87-1.76 (2 H, m, $CH_2C\underline{H_2}CH_2S$), 1.67-1.56 (2 H, m, $C\underline{H_2}CH_2CH_2S$); ^{13}C NMR (75 MHz,): δ 170.55, 170.15, 169.86, 169.55, 138.23, 128.71, 128.41, 126.91, 74.68, 70.63, 70.12, 68.88, 66.75, 62.41, 36.04, 30.52, 27.20, 24.75, 20.86, 20.67, 20.65, 20.59; MS (ESI): 497.30 $[M+H]^+$, calcd. 497.20 for $C_{24}H_{32}SO_9 + H^+$

Compound 102

Compound **102** was prepared according to general procedure described above. Colourless oil, 93 mg, 78% yield. Rf: 0.22 (AcOEt: Hexanes 3: 7); $[\alpha]^{20}_D$ +25.6 (*c* = 1, $CHCl_3$). ^1H NMR (300 MHz,): δ 5.30-5.13 (3 H, m, H-4, H3, H-2), 4.30 (1 H, dd, J = 12.1, 6.3 Hz, H-6), 4.06 (1 H, dd, J = 12.1, 2.7 Hz, H-5), 3.97-3.92 (1 H, m, H-1), 3.87-3.82 (1 H, m, H-6), 2.56-2.45 (4 H, m, $CH_2CH_2C\underline{H_2}S$, $SC\underline{H_2}CH_2CH_2CH_3$), 2.12, 2.07, 2.05, 2.01 (12 H, 4 s, OAc), 1.96-1.85 (2 H, m, $CH_2C\underline{H_2}CH_2S$), 1.72-1.58 (2H, m, $SCH_2C\underline{H_2}CH_2CH_3$), 1.56-1.51 (2 H, m, $C\underline{H_2}CH_2CH_2S$), 1.43-1.31 (2 H, m, $SCH_2CH_2C\underline{H_2}CH_3$), 0.89 (3 H, t, J = 7.4 Hz, $SCH_2CH_2CH_2C\underline{H_3}$); ^{13}C NMR (75 MHz,): δ170.18, 169.92, 169.88, 169.57, 74.70, 70.69, 70.17, 68.90, 66.80, 62.41, 31.63, 31.34, 27.26, 25.23, 21.89, 20.87, 20.67, 20.65, 20.60, 13.53; MS (ESI): 463.30 $[M+H]^+$, calcd. 463.20 for $C_{21}H_{34}SO_9 + H^+$

Compound 103

Compound **103** was prepared according to general procedure described above. Colourless oil, 165 mg, 91% yield. Rf: 0.22 (AcOEt: Hexanes 3: 7); $[\alpha]^{20}_D$ +22.4 (*c* = 1, $CHCl_3$). ^1H NMR (300 MHz,): δ 5.23-5.12 (3 H, m, H-4, H-3, H-2), 4.29 (1 H, dd, J = 12.0, 6.3 Hz, H-6), 4.05 (1 H, dd, J = 12.0, 2.7 Hz, H-5), 3.96-3.91 (1 H, m, H-1), 3.86-3.82 (1 H, m, H-6), 2.55-2.42 (4 H, m, $CH_2CH_2C\underline{H_2}S$, $SC\underline{H_2}CH_2CH_3$), 2.08, 2.07, 2.04, 2.01 (12 H, 4 s, OAc), 1.96-1.84 (2 H, m, $CH_2C\underline{H_2}CH_2S$), 1.71-1.51 (4 H, m, $C\underline{H_2}CH_2CH_2S$, $SCH_2C\underline{H_2}CH_3$), 0.95 (3 H, t, J = 7.41 Hz, $SCH_2CH_2C\underline{H_3}$); ^{13}C NMR (75 MHz,): δ 170.54,

170.15, 169.85, 169.55, 74.66, 70.66, 70.15, 68.87, 66.76, 62.38, 33.95, 31.24, 27.23, 25.22, 22.80, 20.84, 20.64, 20.63, 20.57, 13.36; MS (ESI): 449.20 $[M+H]^+$, calcd. 449.17 for $C_{20}H_{32}SO_9 + H^+$

Compound 104

Compound **104** was prepared according to general procedure described above. Colourless oil, 145 mg, 95% yield. Rf: 0.22 (AcOEt: Hexanes 3: 7); $[\alpha]^{20}_D$ +29.3 (*c* = 1, $CHCl_3$). ^{1}H NMR (300 MHz,): δ 5.27-5.13 (2 H, m, H-4, H-3), 5.12-5.11 (1 H, m, H-2), 4.29 (1 H, dd, J = 12.1, 6.3 Hz, H-6), 4.05 (1 H, dd, J = 12.1, 2.7 Hz, H-5), 3.96-3.91 (1 H, m, H-1), 3.86-3.81 (1 H, m, H-6), 2.59-2.52 (4 H, m, $CH_2CH_2C\underline{H_2}S$, $S C\underline{H_2}CH_3$), 1.91, 1.89, 1.88, 1.73 (12 H, 4 s, OAc), 1.84-1.74 (2 H, m, $CH_2C\underline{H_2}CH_2S$), 1.73-1.59 (2 H, m, $C\underline{H_2}CH_2CH_2S$), 1.22 (3 H, t, J = 7.4 Hz, $SCH_2C\underline{H_3}$); ^{13}C NMR (75 MHz,): δ 170.55, 170.16, 169.86, 169.55, 74.64, 70.65, 70.14, 68.86, 66.76, 62.40, 30.79, 27.22, 25.66, 25.11, 20.84, 20.64, 20.57, 14.64; MS (ESI): 435.16 $[M+H]^+$, calcd. 435.16 for $C_{19}H_{30}SO_9 + H^+$

Compound 105

Compound **105** was prepared according to general procedure described above. Colourless oil, 136 mg, 71% yield. Rf: 0.18 (AcOEt: Hexanes 3: 7); $[\alpha]^{20}_D$ +32.6 (*c* = 1, $CHCl_3$). ^{1}H NMR (300 MHz,): δ 5.25-5.15 (3 H, m, H-4, H-3, H-2), 4.31 (1 H, dd, J = 12.1, 6.0 Hz, H-6), 4.07 (1 H, dd, J = 12.1, 2.7 Hz, H-5), 3.97-3.93 (1 H, m, H-1), 3.87-3.83 (1 H, m, H-6), 2.89 (1 H, sep, J = 6.6 Hz, $SC\underline{H}(CH_3)_2$), 2.62-2.50 (2 H, m, $CH_2CH_2C\underline{H_2}S$), 2.12, 2.08, 2.03, 2.02 (12 H, 4 s, OAc), 1.91-1.86 (2 H, m, $CH_2C\underline{H_2}CH_2S$), 1.75-1.61 (2 H, m, $C\underline{H_2}CH_2CH_2S$), 1.23 (6 H, d, J = 6.6 Hz, $SCH(C\underline{H_3})_2$); ^{13}C NMR (75 MHz,): δ 170.61, 170.22, 169.91, 169.60, 74.74, 70.72, 70.18, 68.94, 66.81, 62.45, 34.74, 29.78, 27.43, 25.45, 23.34, 20.90, 20.68, 20.63; MS (ESI): 449.20 $[M+H]^+$, calcd. 449.17 for $C_{20}H_{32}SO_9 + H^+$

Compound 106

Compound 106 was prepared according to general procedure of de-*O*-acetylation described above. 48 mg, 92% yield. $[\alpha]^{20}_D$ +41.7 (*c* = 1, CH_3OH). ^{1}H NMR (300 MHz, D_2O): δ 7.02-6.92 (5 H, m, Har), 3.70-3.58 (4 H, m, 2 H-6, H-5, H-1), 3.56-3.53 (2 H, m, H-3, H-2), 3.40 (2 H, s, $C\underline{H_2}Ph$), 3.20-3.14 (1 H, m, H-4), 1.49-1.39 (4 H, m, $CH_2C\underline{H_2}CH_2S$, $C\underline{H_2}CH_2CH_2S$); ^{13}C NMR (75 MHz, D_2O): δ 138.16, 128.46, 128.11, 126.54, 77.54, 72.79, 71.16, 70.57, 66.59, 60.74, 35.08, 30.21, 26.26, 24.68; HRMS (ESI): 329.1422 $[M+H]^+$, calcd. 329.1417 for $C_{16}H_{24}SO_5 + H^+$

Compound 107

Compound **107** was prepared according to general procedure of de-*O*-acetylation described above. 58 mg, 95% yield. $[\alpha]^{20}{}_D$ +19.6 (c = 1, CH_3OH). ^1H NMR (300 MHz, D_2O): δ3.78-3.71 (3 H, H-6, H-5, H-1), 3.68-3.59 (2 H, m, H-6, H-3), 3.57-3.50 (1H, m, H-2), 3.35-3.32 (1 H, m, H-4), 2.47-2.38 (4 H, m, $CH_2CH_2C\underline{H_2}S$, $S\underline{CH_2}CH_2CH_2CH_3$), 1.76-1.69 (2 H, m, $CH_2C\underline{H_2}\mathit{CH_2}S$), 1.59-1.37 (4 H, m, $SCH_2C\underline{H_2}CH_2CH_3$, $C\underline{H_2}CH_2CH_2S$), 1.31-1.19 (2 H, m, $SCH_2CH_2C\underline{H_2}CH_3$), 0.76 (3 H, t, J = 7.2 Hz, $SCH_2CH_2CH_2C\underline{H_3}$); ^{13}C NMR (75 MHz, D_2O): δ 77.51, 73.08, 71.18, 70.56, 66.65, 60.75, 30.94, 30.79, 26.36, 25.04, 21.24, 21.24, 12.87; HRMS (ESI): 317.1407 $[M+Na]^+$, calcd. 317.1501 for $C_{13}H_{26}SO_5 + H^+$

Compound 108

Compound **108** was prepared according to general procedure of de-*O*-acetylation described above. 86 mg, 92 % yield. $[\alpha]^{20}{}_D$ +23.6 (c = 1, CH_3OH). ^1H NMR (300 MHz, D_2O): δ 3.79-3.71 (3 H, m, H-6, H-5, H-1), 3.67-3.63 (1 H, m, H-6), 3.56 (1 H, dd, J = 12.1, 5.6 Hz, H-3), 3.51-3.44 (1H, m, H-2), 3.39-3.34 (1H, m, H-4), 1.57-1.38 (4 H, m, $CH_2CH_2C\underline{H_2}S$, $S\underline{CH_2}CH_2CH_3$), 1.76-1.67 (2 H, m, $CH_2C\underline{H_2}CH_2S$), 1.57-1.38 (4 H, m, $C\underline{H_2}CH_2CH_2S$, $SCH_2C\underline{H_2}CH_3$), 0.79 (3 H, t, J = 7.3 Hz, $SCH_2CH_2C\underline{H_3}$); ^{13}C NMR (75 MHz, D_2O): δ 77.52, 73.07, 71.04, 70.39, 66.85, 60.79, 32.80, 30.25, 26.08, 24.75, 21.89, 12.32; HRMS (ESI): 281.1417 $[M+H]^+$, calcd. 281.1344 for $C_{12}H_{24}SO_5 + H^+$

Compound 109

Compound **109** was prepared according to general procedure of de-*O*-acetylation described above. 39 mg, 91% yield. $[\alpha]^{20}{}_D$ +13.9 (c = 1, CH_3OH). ^1H NMR (300 MHz, D_2O): δ 3.76-3.69 (4 H, m, 2 H-6, H-5, H-1), 3.61-3.41 (3 H, m, H-4, H-3, H-2), 2.51-2.43 (4 H, m, $CH_2CH_2C\underline{H_2}S$, $SC\underline{H_2}CH_3$), 1.75-1.71 (2 H, m, $CH_2C\underline{H_2}CH_2S$), 1.65-1.48 (2 H, m, $C\underline{H_2}CH_2CH_2S$), 1.11 (3 H, t, J = 7.2 Hz, $SCH_2C\underline{H_3}$); ^{13}C NMR (75 MHz, D_2O): δ 77.57, 73.08, 71.05, 70.37, 66.88, 60.81, 29.76, 26.07, 24.64, 13.48; HRMS (ESI): 267.1266 $[M+H]^+$, calcd. 267.1260 for $C_{11}H_{22}SO_5 + H^+$

Compound 110

Compound **110** was prepared according to general procedure of de-*O*-acetylation described above. 68 mg, 91% yield. $[\alpha]^{20}{}_D$ +23.8 (c = 1, CH_3OH). ^1H NMR (300 MHz, D_2O): δ 3.79-3.71 (3 H, m, H-6, H-5, H-1), 3.67-3.63 (1 H, m, H-6), 3.57 (1 H, dd, J = 11.8, 5.8 Hz, H-3), 3.51-3.44 (1 H, m, H-2), 3.38-3.33 (1 H, m, H-4), 2.87 (1 H, sep, J = 6.7 Hz, $SC\underline{H}(CH_3)_2$), 2.57-2.43 (2 H, m, $CH_2CH_2C\underline{H_2}S$), 1.80-1.67 (2 H, m, $CH_2C\underline{H_2}CH_2S$), 1.57-1.39 (2 H, m, $C\underline{H_2}CH_2CH_2S$), 1.10 (6 H, d, J = 6.6 Hz, $SCH(C\underline{H_3})_2$); ^{13}C NMR (75

MHz, D_2O): δ 77.52, 73.05, 71.02, 70.35, 66.83, 60.77, 34.07, 28.76, 26.16, 24.86, 22.06; HRMS (ESI): 303.1244 $[M+Na]^+$, calcd. 303.1344 for $C_{12}H_{24}SO_5 + Na^+$

Compound 111

Compound **111** was prepared according to general Heck cross coupling procedure described above. Colourless oil, 61 mg, 92% yield. Rf: 0.27 (AcOEt: Hexanes 3: 7); $[\alpha]^{20}_D$ +24.3 (*c* = 1, $CHCl_3$). ^1H NMR (300 MHz, $CDCl_3$): δ 7.45-7.15 (5 H, m, Har), 6.51 (1 H, d, J = 15.6 Hz, CH=CHPh), 6.20-6.06 (1 H, m, CH=CHPh), 5.31 (1 H, dd, J = 8.52, 3.3 Hz, H-3), 5.24-5.15 (2 H, m, H-2, H-4), 4.34 (1 H, dd, J = 12.1, 6.8 Hz, H-6), 4.19-4.06 (2 H, m, H-1, H-6), 3.95 (1 H, td, J = 11.2, 2.7 Hz, H-5), 2.74-2.55 (2 H, m, CH_2CH=CHPh). 2.10, 2.05 (12H, 4s, 4 OAc); ^{13}C NMR (75 MHz, $CDCl_3$): δ 170.6, 170.13, 169.87, 169.63, 136.86, 133.07, 129.57, 128.45, 127.38, 126.09, 132.93, 73.93, 70.64, 68.62, 67.03, 62.24, 32.78, 20.86, 20.70, 20.64, 20.47; MS (ESI): 449.19 $[M+H]^+$, calcd. 449.17 for $C_{23}H_{28}O_9 + H^+$

Compound 112

Compound **112** was prepared according to general procedure described above. Colourless oil, 320 mg, 87% yield. Rf: 0.18 (AcOEt: Hexanes 3: 7); $[\alpha]^{20}_D$ +22.6 (*c* = 1, $CHCl_3$). ^1H NMR (300 MHz, $CDCl_3$): δ 7.62-7.36 (9 H, m, Har), 6.57 (1 H, d, J = 15.9 Hz, CH=CHPh), 6.22 (1 H, dt, J = 15.9, 7.1 Hz, CH=CHPh), 5.35 (1 H, dd, J = 8.5, 3.3 Hz, H-3), 5.28-5.22 (2 H, m, H-4, H-2), 4.38 (1 H, dd, J = 12.1, 6.8 Hz, H-6), 4.20-4.10 (2 H, m, H-6, H-1), 3.99 (1 H, m, H-5), 2.27-2.57 (2 H, m, CH_2CH=CHPh), 2.15-2.04 (12 H, 4s, 4 OAc); ^{13}C NMR (75 MHz, $CDCl_3$): δ 170.68, 170.17, 169.91, 169.66, 140.59, 140.16, 135.95, 132.69, 128.73, 127.25, 127.16, 126.84, 126.56, 125.75, 124.11, 73.98, 70.75, 70.07, 68.67, 67.09, 62.29, 32.91, 20.89, 20.73, 20.67, 20.55; MS (ESI): 525.20 $[M+H]^+$, calcd. 525.20 for $C_{29}H_{32}O_9 + H^+$

Compound 113

Compound **113** was prepared according to general procedure described above. Colourless oil, 52 mg, 98% yield. Rf: 0.25 (AcOEt: Hexanes 3: 7); $[\alpha]^{20}_D$ +18.17 (*c* = 1, $CHCl_3$). ^1H NMR (300 MHz, $CDCl_3$): δ 7.61-7.58 (1 H, m, Har), 7.46-7.30 (8 H, m, Har), 6.5 (1 H, d, J = 15.6 Hz, CH=CHPh), 6.1 (1 H, dt, J = 15.6, 6.8 Hz, CH=CHPh), 5.29-5.20 (3 H, m, H-4, H-3, H-2), 4.34 (1 H, d, J = 12.1, 6.3 Hz, H-6), 4.31-4.04 (2 H, m, H-6, H-1), 3.88 (1 H, m, H-5), 2.62-2.49 (2 H, m, CH_2CH=CHPh), 2.13-2.06 (12 H, 4s, 4 OAc); ^{13}C NMR (75 MHz, $CDCl_3$): δ 170.58, 170.07, 169.75, 169.55, 140.73, 140.37, 134.92, 132.10,

130.04, 129.65, 127.97, 127.35, 127.30, 126.91, 129.96, 124.80, 74.24, 70.53, 69.94, 68.59, 66.84, 62.20, 32.81, 20.84, 20.64, 20.58, 20.51; MS (ESI): 547.39 $[M+Na]^+$, calcd. 547.19 for $C_{29}H_{32}O_9 + Na^+$

Compound 114

Compound **114** was prepared according to general procedure described above. Colourless oil, 300 mg, 89% yield. Rf: 0.22 (AcOEt: Hexanes 3: 7); $[\alpha]^{20}_D$ +19.6 (*c* = 1, $CHCl_3$). ^{1}H NMR (300 MHz, $CDCl_3$): δ 8.12 (2 H, d, J = 7.7 Hz, Har), 7.86-7.76 (2 H, m, Har), 7.59-7.41 (4 H, m, Har), 7.28 (1 H, d, J = 15.6 Hz, CH=C<u>H</u>Ph), 6.25-6.15 (1 H, m, C<u>H</u>=CHPh), 5.40-5.21 (3 H, m, H-4, H-3, H-2), 4.38 (1 H, dd, J = 11.8, 6.6 Hz, H-6), 4.26-4.11 (2 H, m, H-6, H-1), 4.05-4.01 (1 H, m, H-5), 2.89-2.65 (2 H, m, <u>CH_2</u>CH=CHPh), 2.10-2.0 (12 H, 4s, 4 OAc); ^{13}C NMR (75 MHz, $CDCl_3$): δ 170.71, 170.23, 169.97, 169.68, 134.76, 133.52, 130.53, 128.45, 127.84, 127.32, 126.04, 125.75, 125.54, 123.83, 74.20, 70.79, 70.11, 68.74, 67.05, 62.38, 33.12, 20.96, 20.78, 20.72, 20.56; MS (ESI): 499.34 $[M+H]^+$, calcd. 499.20 for $C_{27}H_{30}O_9 + H^+$

Compound 115

Compound 115 was prepared according to general procedure of de-*O*-acetylation described above. 18 mg, 88% yield. $[\alpha]^{20}_D$ +19.8 (*c* = 1, CH_3OH). ^{1}H NMR (300 MHz, CD_3OD): δ 7.38-7.35 (2 H, m, Har), 77.28-7.23 (2 H, m, Har), 7.19-7.14 (1 H, m, Har), 6.49 (1 H, d, J = 15.9 Hz, CH=C<u>H</u>Ph), 6.33-6.23 (1 H, m, C<u>H</u>=CHPh), 3.98 (1 H, td, J = 6.6, 2.4 Hz, H-6), 3.82-3.80 (1 H, m, H-5), 3.78-3.72 (3 H, m, H-6, H-3, H-1), 3.67-3.62 (1 H, m, H-2), 3.56-3.50 (1 H, m, H-4), 2.69-2.59 (1 H, m, <u>CH_2</u>CH=CH), 2.54-2.44 (1 H, m, <u>CH_2</u>CH=CH). ^{13}C NMR (75 MHz, CD_3OD): δ 138.88, 133.46, 129.48, 128.14, 127.17, 127.12, 78.65, 76.26, 72.61, 72.14, 69.37, 63.03, 33.94; HRMS (ESI): 303.1202 $[M+Na]^+$, calcd. 303.1203 for $C_{15}H_{20}O_5 + Na^+$

Compound 116

Compound **116** was prepared according to general procedure of de-*O*-acetylation described above. 29.3 mg, 96% yield. $[\alpha]^{20}_D$ +30.2 (*c* = 1, CH_3OH). ^{1}H NMR (300 MHz, CD_3OD): δ 7.63 (4 H, dd, J = 12.8, 7.9 Hz, Har), 7.48-7.31 (5 H, m, Har), 6.5 (1 H, d, J = 15.6 Hz, CH=C<u>H</u>Ph), 6.40-6.31 (1 H, m, C<u>H</u>=CHPh), 4.74-4.62 (1 H, m, H-6), 4.49-4.38 (1 H, m, H-5), 3.79-3.78 (1 H, m, H-1), 3.58-3.41 (4 H, m, H-4, H-3, H-2), 2.49-2.44 (2 H, m, <u>CH_2</u>CH=CH); ^{13}C NMR (75 MHz, CD_3OD): δ 139.71, 138.56, 130.55, 128.92, 127.33, 126.74, 126.44, 126.39, 76.17, 74.96, 70.98, 69.69, 68.24, 61.14, 33.24. ESI^+-HRMS: $[M+NH_4]^+$ calcd for $C_{21}H_{28}NO_5$, 374.1962; found, 374.1960. HRMS (ESI): 379.1515 $[M+Na]^+$, calcd. 379.1521 for $C_{21}H_{24}O_5 + Na^+$

Compound 117 : full structural characterization

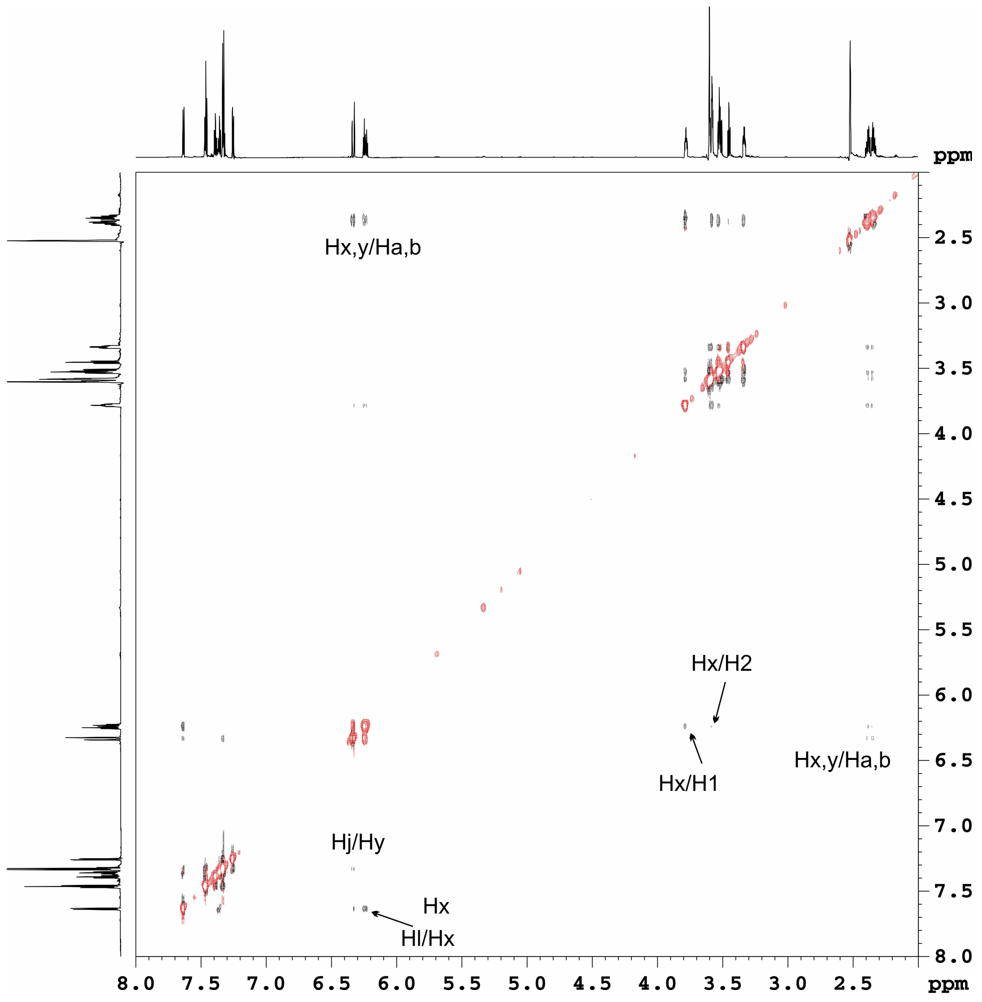

Figure S13. High field (900 MHz) NMR ROESY spectra of **117** in DMSO-d_6.

Compound **117** was prepared according to general procedure of de-*O*-acetylation described above. 31 mg, 92% yield. $[\alpha]^{20}_{D}$ +27.2 (*c* = 1, CH_3OH). ^{1}H NMR (300 MHz, CD_3OD): δ 7.62-7.59 (1 H, m, Har), 7.43-7.18 (8 H, m, Har), 6.4 (1 H, d, J = 15.6 Hz, CH=CHPh), 6.23-6.13 (1 H, m, CH=CHPh), 3.96 (1 H, td, J = 6.6, 2.2 Hz, H-6), 3.77-3.67 (3 H, m, H-6, H-5, H-1), 3.69-3.63 (2 H, m, H-3, H-2), 3.60-3.44 (1 H, m, H-4), 2.55-2.47 (1 H, m, CH_2CH=CH), 2.41-2.32 (1 H, m, CH_2CH=CH); ^{13}C NMR (75 MHz, CD_3OD): δ 142.45, 141.78, 136.65, 132.38, 130.77, 129.15, 128.52, 128.20, 128.15, 128.05, 127.08, 78.73, 76.02, 72.54, 72.19, 69.27, 62.98, 34.16; MS (ESI): 379.15 $[M+Na]^+$, calcd. 379.15 for $C_{21}H_{24}O_5 + Na^+$

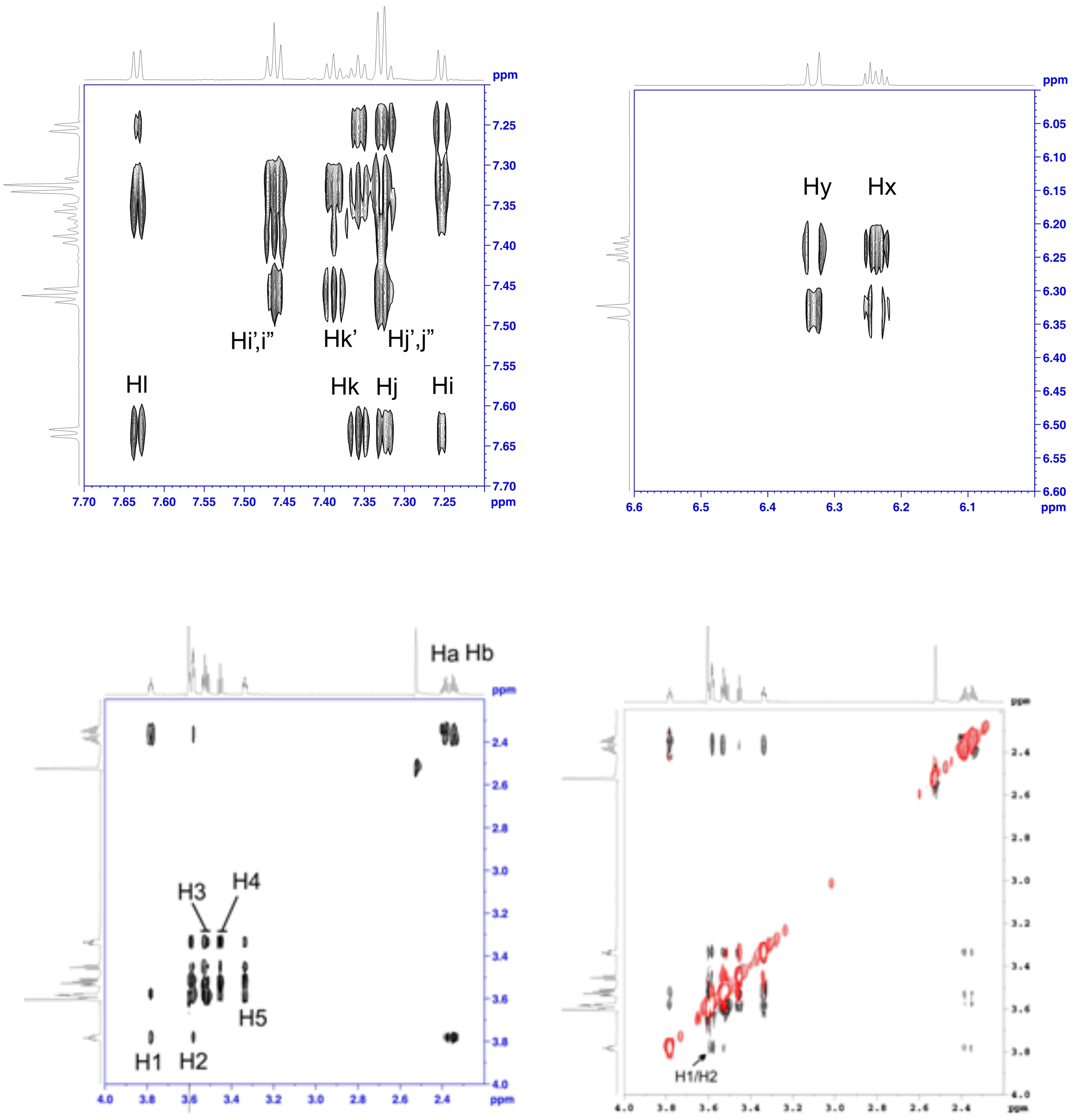

Figure S14-S16. ^{1}H-NMR analysis of **117** in DMSO-d_6.

Proton	δ	δ Carbon	Coupling constants (Hz)	main nOe contacts with
H1	3,783	76,8		H2
H2	3,582	71,3	$^3J_{2,1}$: 2.7 $^3J_{2,3}$: 3.1	
H3	3,531	72,4	$^3J_{3,4}$: 7.5	
H4	3,453	69,3	$^3J_{4,5}$: 7.5	
H5	3,335	77,3	$^3J_{5,6}$: 7.0 $^3J_{5,6'}$: 3.2	
H6	3,598	62,6	$^2J_{6,6'}$: 12.0	
H6'	3,517			
Ha	2,385	35,3		Hx, Hy
Hb	2,347			Hx,Hy
Hx	6,238	130		Hl, H1
Hy	6,331	131,2	$^3J_{x,y}$: 15.3	Ha, Hb,Hi',i"
Hl	7,634	127,5	$^3J_{l,j}$: 7.3 $^4J_{l,k}$: 1.7	Hx, Hj
Hj	7,358	128,9		
Hk	7,325	129		
Hi	7,254	131,5	$^3J_{i,k}$: 7.3 $^4J_{l,j}$: 1.7	Hi' or i"
Hj',j"	(t)7.462	129,8	$^3J_{i',i''}$: 7.6	
Hi',i"	(d)7.329	131,1	$^3J_{j',j''}$: 6.8	
Hk'	7,388	128,8	$^3J_{k,j',j''}$: 7.3	

**Quaternary carbon for benzyl (ppm)**	
	Cbzl 1,1 : 136.5
	Cbzl 1,2 : 141.2
	Cbzl 2,1 : 141.9

Table S1. Results from the detailed analysis of **117** by 600 MHz NMR.

Assignments based on COSY, ROESY and TOCSY. Value obtained at 293 K in DMSO, quaternary carbons were attributed with the help of HMBC experiments.

Compound 118

Compound **118** was prepared according to general procedure of de-*O*-acetylation described above. 82 mg, 97 % yield. $[\alpha]^{20}_D$ +13.6 (c = 1, CH_3OH). ^{1}H NMR (300 MHz, DMSO-d6): δ 8.19-8.17 (1 H, m, Har), 7.94-7.79 (2 H, m, Har), 7.66-7.46 (4 H, m, Har), 7.26 (1H, d, J = 15.9 Hz, CH=C<u>H</u>Ph), 6.35-6.28 (1 H, m, C<u>H</u>=CHPh), 4.76-4.65 (2H, m, H-6, H-5), 4.52-4.41 (2 H, m, H-6, H-1), 3.64-3.32 (3 H, m, H-4, H-3, H-2), 2.59-2.49 (2 H, m, <u>CH_2</u>CH=CH); ^{13}C NMR (75 MHz, DMSO-d6): δ 134.81, 133.25, 130.75,

130.44, 128.32, 127.96, 127.20, 126.03, 125.77, 125.36, 123.77, 123.17, 76.12, 75.00, 71.04, 69.81, 68.26, 61.16, 33.40; HRMS (ESI): 353.1365 $[M+Na]^+$, calcd. 353.1364 for $C_{19}H_{22}O_5 + Na^+$

Compound 119

Compound 119 was prepared according to general procedure of hydrogenation described above. 53.5 mg, 95% yield. $[\alpha]^{20}_D$ +33.5 (*c* = 1, CH_3OH). ^1H NMR (300 MHz, CD_3OD): δ 7.17-7.01 (5 H, m, Har), 3.79-3.70 (1H, m, H-6), 3.68 (1 H, dd, J = 11.8, 2.7 Hz, H-5), 3.63-3.51 (4 H, m, H-6, H-3, H-2, H-1), 3.27-3.24 (1 H, m, H-4), 2.60-2.49 (2H, m, $CH_2CH_2C\underline{H_2}Ph$), 1.75-1.52 (3 H, m, $\underline{H}CHC\underline{H_2}CH_2Ph$), 1.42-1.32 (1 H, m, $HC\underline{H}CH_2CH_2Ph$); ^{13}C NMR (75 MHz, CD_3OD): δ 143.45, 129.4, 129.3, 126.75, 78.82, 75.56, 73.08, 72.78, 69.19, 62.98, 36.41, 29.05, 28.81; HRMS (ESI): 283.1539 $[M+H]^+$, calcd. 283.1540 for $C_{15}H_{22}O_5 + H^+$

Compound 120

Compound **120** was prepared according to general procedure of hydrogenation described above. 48.2 mg, 96% yield. $[\alpha]^{20}_D$ +21.8 (*c* = 1, CH_3OH). ^1H NMR (300 MHz, CD_3OD): δ 7.49-7.41 (4 H, m, Har), 7.33-7.27 (2 H, m, Har), 7.22-7.18 (3 H, m, Har), 3.81-3.75 (1 H, m, H-6), 3.69 (1 H, dd, J = 11.8, 2.7 Hz, H-5), 3.62-3.48 (4 H, m, H-6, H-3, H-2, H-1), 3.31-3.27 (1 H, m, H-4), 2.65-2.52 (2H, m, $CH_2CH_2C\underline{H_2}Ph$), 1.79-1.65 (2H, m, $CH_2C\underline{H_2}CH_2Ph$), 1.45-1.37 (2 H, m, $C\underline{H_2}CH_2CH_2Ph$); ^{13}C NMR (75 MHz, CD_3OD): δ 142.68, 142.37, 139.99, 129.96, 129.78, 128.03, 127.90, 127.78, 78.80, 75.70, 73.11, 72.84, 69.31, 63.11, 36.03, 29.12, 28.74; HRMS (ESI): 381.1682 $[M+Na]^+$, calcd. 381.1672 for $C_{21}H_{26}O_5 + Na^+$

Compound 121

Compound **121** was prepared according to general procedure of hydrogenation described above. 48.2 mg, 96% yield. $[\alpha]^{20}_D$ +18.4 (*c* = 1, CH_3OH). ^1H NMR (300 MHz, CD_3OD): δ 7.45-7.21 (7 H, m, Har), 7.13 (1 H, d, J = 7.1, Har), 7.0 (1 H, d, J = 7.1, Har), 3.58-3.52 (1 H, m, H-6), 3.44-3.26 (4 H, m, H-6, H-5, H-3, H-1), 3.16-3.14 (2H, m, H-2, H-4), 2.56-2.48 (2H, m, $CH_2CH_2C\underline{H_2}Ph$), 1.56-1.43 (3 H, m, $\underline{H}CHC\underline{H_2}CH_2Ph$), 1.38-1.13 (1 H, m, $HC\underline{H}CH_2CH_2Ph$). ^{13}C NMR (75 MHz, CD_3OD): δ 142.14, 141.36, 141.28, 139.40, 130.23, 129.68, 128.90, 128.17, 127.52, 75.38, 74.98, 71.16, 70.54, 68.05, 61.25, 32.39, 28.64, 27.04; HRMS (ESI): 359.1855 $[M+H]^+$, calcd. 359.1853 for $C_{21}H_{26}O_5 + H^+$

Compound 122

Compound **122** was prepared according to general procedure of debenzylation described above. 57.3 mg, 96% yield. $[\alpha]^{20}_D$ +16.8 (*c* = 1, CH_3OH). ^{1}H NMR (300 MHz, CD_3OD): δ 7.95 (1 H, d, J = 7.9 Hz, Har), 7.69-7.55 (2 H, m, Har), 7.39-7.21 (4 H, m, Har), 3.80-3.77 (1 H, m, H-6), 3.69-3.94 (4 H, m, H-6, H-5, H-3, H-1), 3.28-3.20 (1 H, m, H-2), 3.04-2.99 (1 H, m, H-4), 2.62-2.54 (2H, m, $CH_2CH_2C\underline{H_2}Ph$), 1.82-1.55 (2H, m, $CH_2C\underline{H_2}CH_2Ph$), 1.49-1.40 (2H, m, $C\underline{H_2}CH_2CH_2Ph$); ^{13}C NMR (75 MHz, CD_3OD): δ 139.52, 135.40, 133.15, 129.90, 129.69, 127.57, 126.99, 126.72, 126.40, 124.84, 78.65, 75.60, 73.09, 72.81, 69.17, 62.92, 33.28, 29.43, 28.18; HRMS (ESI): 333.1700 $[M+H]^+$, calcd. 333.1696 for $C_{19}H_{24}O_5 + H^+$

Compound 123

Compound **123** was prepared according to general procedure of hydrogenation of **78** as described above. 48 mg, 95% yield. $[\alpha]^{20}_D$ +24.6 (*c* = 1, CH_3OH). ^{1}H NMR (300 MHz, CD_3OD): δ 3.75-3.69 (1 H, m, H-6), 3.65 (m, dd, J = 11.8, 2.7 Hz, H-5), 3.60-3.48 (4 H, m, H-6, H-3, H-2, H-1), 3.29-3.23 (1 H, m, H-4), 1.67-1.57 (1 H, m, $\underline{H}CHCH_2CH_3$), 1.42-1.17 (3H, m, $HC\underline{H}C\underline{H_2}CH_3$), 0.82 (3H, t, J = 7 Hz, $CH_2C\underline{H_3}$); ^{13}C NMR (75 MHz, CD_3OD): δ 78.75, 75.48, 73.14, 72.83, 69.22, 63.06, 31.74, 20.13, 14.17; HRMS (ESI): 207.1221 $[M+H]^+$, calcd. 207.1227 for $C_9H_{18}O_5 + H^+$

Surface plasmon resonance solution affinity

Synthesis of 8-aminooctyl α-D-mannopyranoside (5b) as a competitive steady state binder

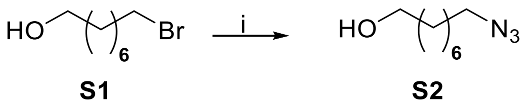

Reagents: (i) NaN_3, DMF, 80° C, 2 h, 97%

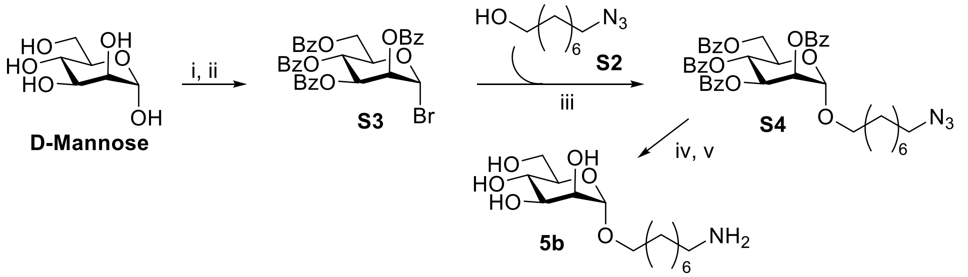

Scheme S5. Reagents: (i) BzCl, DMAP, pyridine, 0° C → rt, 36 h, quant.; (ii) HBr (33% in AcOH), AcOH, 0° C → rt, 36 h; (iii) AgOTf, DCM, 0° C → rt, 24 h, 85% over 2 steps; (iv) NaOMe, MeOH, rt, 2 h, 95%; (v) Pd/C, H_2, MeOH, (H-cube), 80%.

1-Azidooctan-8-ol (**S2**). Compound **S1** (200 mg, 0.96 mmol) and sodium azide were dissolved in DMF (5 mL) and stirred for 2 h at 80 °C. The reaction mixture was poured onto ice-water (50 mL) and stirred for 30 min, extracted two times with EtOAc (25 mL), dried over Na_2SO_4, filtrated, concentrated *in vacuo* and purified with flash column chromatography (toluene) to yield compound **S2** (156 mg, 0.931mmol, 97%). ^{1}H NMR (500 MHz, $CDCl_3$): δ = 3.62 (t, *J*=6.5, 2H) and 3.24 (t, *J*=7.0, 2H) (H-1, H-8), 1.54-1.63 (m, 5H, H-2, H-7, OH), 1.34-1.41 (m, 8H, H-3 - H-6). ^{13}C NMR (125 MHz, $CDCl_3$): δ = 62.9 ($\mathit{C}H_2OH$), 51.4 ($\mathit{C}H_2N_3$), 32.7, 29.2, 29.0, 28.8, 26.6 and 25.6 (C-2-7).

8-Azidooctyl 2,3,4,6-tetra-O-benzoyl-α-D-mannopyranoside (**S4**). D-Mannose (1.0 g, 5.6 mmol) was dissolved in pyridine (20 mL) and DMAP (catalytic amount, 15 mg) was added. Benzoyl chloride (10.2 mL, 88.8 mmol) was added dropwise. After 36 h the solvent was removed under reduced pressure and co-evaporated with toluene and purified by flash silica gel chromatography (toluene) to give perbenzoylated mannose which was dissolved in HBr/HOAc (39% HBr in AcOH, 45 mL) and the mixture was stirred for 36 h at rt. The reaction mixture was diluted with CH_2Cl_2 (75 mL) and washed with ice water and $NaHCO_3$ (aqueous saturated solution, 50 mL), dried over $MgSO_4$, filtrated and concentrated *in vacuo* to give crude compound **S3**. The bromo sugar **S3** (152 mg, 0.23 mmol) was dissolved in dry CH_2Cl_2 (2 mL) and stirred for 15 min in the presence of molecular sieves (4 Å) at 0 °C under an Ar atmosphere. Then a solution of AgOTf (77 mg, 0.30 mmol) and **S2** (80 mg, 0.48 mmol) in toluene (1 mL) was added drop wise. After 24 h TLC (1:1, CH_2Cl_2-toluene) showed complete reaction and the mixture was filtered through Celite and concentrated. Purification using silica gel flash chromatography gave compound **S4** (148 mg, 0.197 mmol, 85%). R_f.: 0.72 (toluene). ^{1}H NMR (500 MHz, $CDCl_3$): δ = 8.14 – 8.02 (m, 4H), 8.01 – 7.93 (m, 2H), 7.88 – 7.79 (m, 2H), 7.63 – 7.53 (m, 2H), 7.49 (t, *J*=7.4, 1H), 7.45 – 7.32 (m, 7H), 7.25 (t, *J*=7.8, 2H), (all H-aromatic), 6.11 (t, *J*=10.1, 1H, H-4), 5.94 (dd, *J*=10.1, 3.3, 1H, H-3), 5.70 (dd, *J*=3.1, 1.8, 1H, H-2), 5.10 (d, *J*=1.4, 1H, H-1), 4.71 (dd, *J*=12.1, 2.5, 1H, H-6_a), 4.50 (dd, *J*=12.1, 4.6, 1H, H-6_b), 4.46 – 4.40 (m, 1H, H-5), 3.83 (dt, *J*=9.6, 6.7, 1H, H-$1'_a$), 3.58 (dt, *J*=9.6, 6.6, 1H, H-$1'_b$), 3.26 (t, *J*=7.0, 2H, $C\mathit{H}_2N_3$), 1.88 – 1.66 (m, 2H, H-2’), 1.66 – 1.56 (m, 2H, H-7’), 1.50 – 1.30 (m, 8H, H-3’-H-6’). ^{13}C NMR (125 MHz, $CDCl_3$): δ = 166.13, 165.51 (double peak) and 165.46 (C=O), 133.43, 133.40, 133.14 and 133.04 (C-quaternary), 129.92, 129.84, 129.79, 129.72, 129.72, 129.41, 129.15, 129.05, 128.57, 128.42 and 128.29 (C-aromatic), 97.67 (C-1), 70.69 (C-2), 70.18 (C-3), 68.88 (C-5), 68.74 (C-1’), 67.13 (C-4), 63.01 (C-6), 51.46 ($\mathit{C}H_2N_3$), 29.34, 29.23, 29.06, 28.84, 26.66 and 26.05 (C-2’ – C-7’). LRMS $[M+Na]^+$ calculated for $C_{42}H_{43}N_3O_{10}$: 772.8, found 772.4

8-Aminooctyl α-D-mannopyranoside (**5b**). Compound **S4** (148 mg, 0.197 mmol) was dissolved in MeOH (5 mL) and CH_2Cl_2 (1 mL) and treated with NaOMe (0.2 mL, 1M in MeOH). After stirring for 2 h at rt the reaction was neutralized using Dowex® acidic ion-exchange resin and purified by silica gel flash column chromatography (EtOAc-MeOH, 7:2) to give the 8-azidooctyl α-D-mannopyranoside (60 mg, 95%) as a clear resin. This azide (60 mg, 0.180 mmol) was dissolved in MeOH (6 mL) and hydrogenated using an H-cube at 25° C, 30 bar and a flow rate of 0.3 mL/min. Purification over a 600 mg C18 MAXI-CLEAN cartridge (Alltech) afforded compound **5b** (48 mg, 80%). R_f: 0.15 (9:3:2:0.1, EtOAc/DCM/MeOH/H_2O); ^{1}H NMR (500 MHz, D_2O): δ = 4.76 (s, 1H, H-1), 3.57 (m 3H); 3.71 (m, 2H), 3.43 (m, 1H); 3.51 (m, 2H), 2.54 (t, *J*=7.0, 2H), 1.23-1.52 (m, 12H, H-3' - H-6'). ^{13}C NMR (125 MHz, D_2O): δ = 99.7 (C-1), 72.7, 70.7, 70.1, 67.9 and 66.8 (C-2-5, C-1'), 60.9 (C-6), 40.4 (CH_2NH_2), 31.0, 28.5, 28.43, 28.41, 26.0 and 25.3 (C-2'-7'). LRMS $[M+H]^+$ calculated for $C_{14}H_{29}NO_6$: 308.4, found 308.2.

Steady state affinity measurement with compound 8-aminooctyl α-D-mannopyranoside (5b)

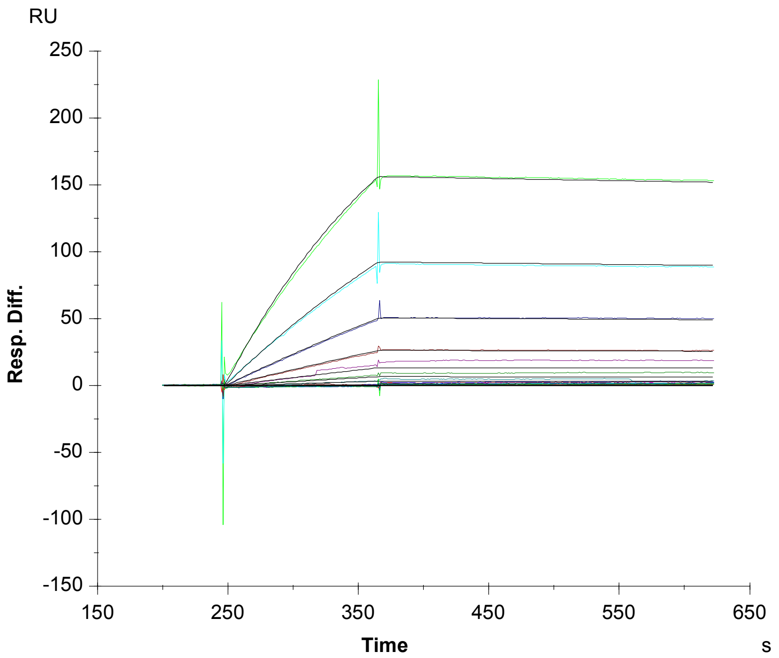

Figure S18. Steady state affinity (K_d = 16.9 nM ± 0.4, chi^2 = 3.37) of FimH lectin with **5b**

<u>SPR measurements and solution affinity fits for lead compounds oligomannose-3, n-heptyl α-D-mannose (5) and n-octyl α-D-mannose (5a)</u>

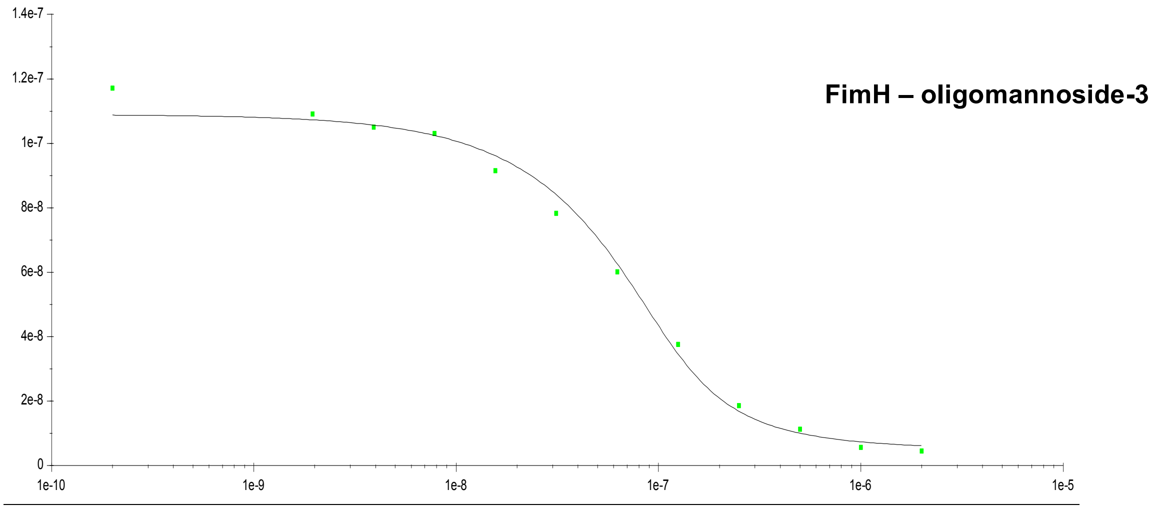

KA(1/M)	KD(M)	Initial Conc B	constant	Chi2
49462067.1	2.02175133e-8	5.10445473e-9	1.70E-17	1.0371967e-7

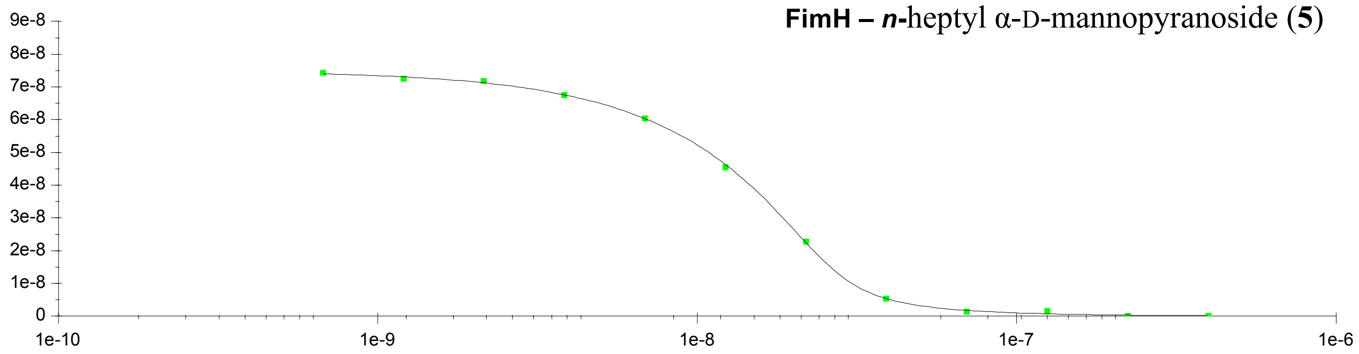

KA(1/M)	KD(M)	Initial Conc B	Chi2
231691100	4.31609155e-9	7.49411143e-8	2.51E-19

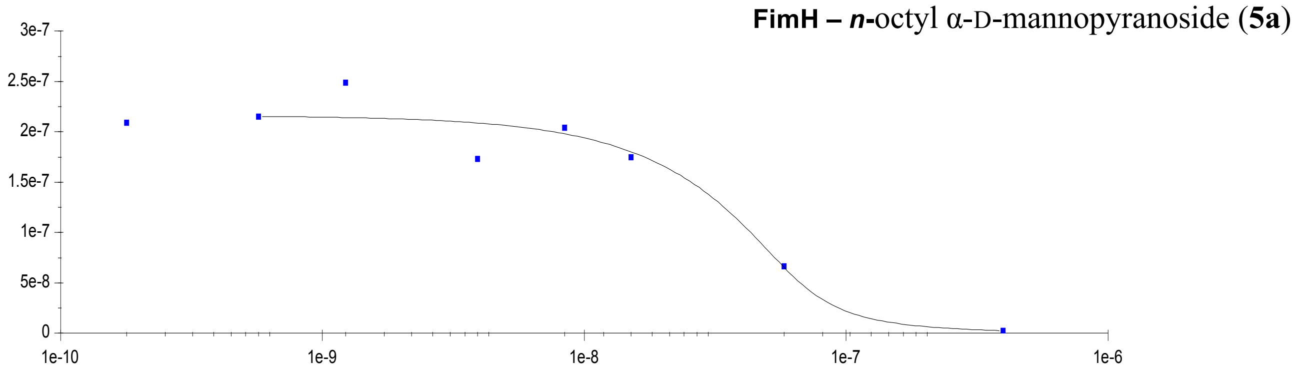

KA(1/M)	KD(M)	Initial Conc B	Chi2
46550590.5	2.14820046e-8	2.16099467e-7	5.11E-16

Figure S19. Inhibition curves and fitted equilibrium constants for lead compounds with FimH

The FimH lectin domain concentrations were plotted in function of the mannoside concentration to obtain inhibition curves. The fit of the solution affinity interaction model yielded the FimH – mannoside affinity

in most cases. Exceptions were compounds that either had low solubility in aqueous solvents, or subnanomolar affinity compounds. The latter showed an apparent stoichiometrical binding to FimH within the concentration range of sugar from micromolar up to nanomolar, for which no solution affinity sigmoidal could be fitted (**Figure S10**). The affinity of these compounds (**62**, **118**, **120** and **122**) was marked by nf (no good fit could be obtained).

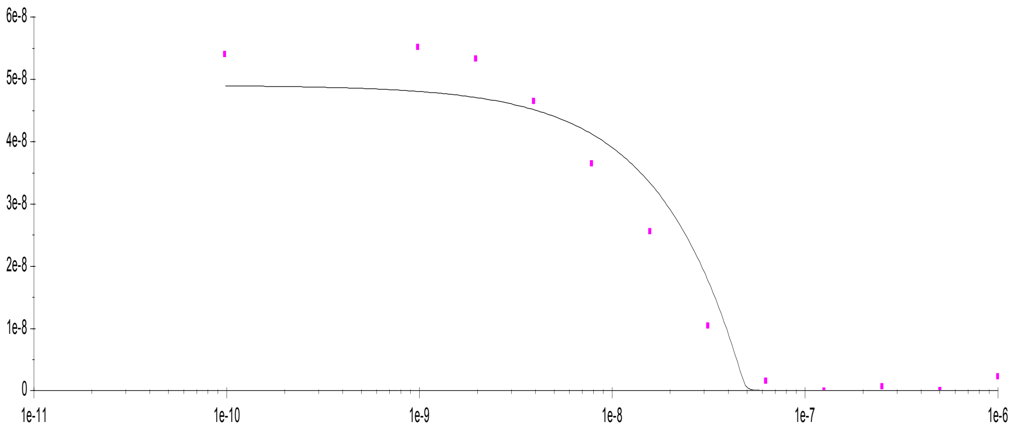

Figure S20. Example of a linear titration curve (fuchsia) that does not follow a sigmoidal fit (black line)

This apparent stoichiometric binding to FimH was obtained for inhibitors with low solubilities and nanomolar affinities. Although SPR did thus not allow to measure subnanomolar affinities, HAI (this manuscript) and ITC measurements [15] confirmed that none of the ligands has subnanomolar affinities for FimH.

<u>Comparisons of the potency of compounds using inhibition assays based on whole cell interactions (HAI) *vs* based on molecular interactions either via competition (SPR) or direct (ITC)</u>

In this tabulated comparison appear two series of *O*-linked compounds with linkers from alkynyl over alkenyl to alkylyl to otherwise the structurally similar substituents: series **51**, **23**, and **27**, and series **53**, **25** and **29**. When combining HAI, SPR and ITC data for the first series, **51**, **23**, **27**, as well as the second one, **53**, **25**, **29**, it can be concluded that in both cases the alkenyl linker is favored. Alkylyls are slightly less soluble than alkenyls and it is presumable that **27** and **29** have suffered from the lack of DMSO in the SPR experiment, indicated by the observation in the HAI assay that they are equally well or better inhibitors as compared to the compounds with alkenyl linkers. By enabling complete solubility of the compound in the HAI assay, using 10% DMSO, the HAI data disambiguate the higher affinities for a double bond (**22**-**25**) over a single bond (**26**-**29**) at post-glycosidic linkage atomic positions two to three. Whereas SPR displays non-fitable data in competition assay for less water-soluble compounds with close to nanomolar or subnanomolar affinities, such as for the alkenyl **62**, the HAI assay overcomes this problem because it is very tolerant to the presence of DMSO.

**Cpd**	**K_d SPR (nM)**	**IC_{50} HAI (μM)**	**K_d ITC (nM)**
**51**	405 ± 24	100	60 ± 9.5
**23**	3.0 ± 1.1	25	nd
**27**	10 ± 0.3	25	1.5 ± 0.3
**53**	120 ± 18	50	nd
**25**	5.0 ± 1.4	50	71 ± 23
**29**	22 ± 3.0	25	nd
**5**	4.3 ± 0.3	6.25	7.7 ± 1.7
**61**	6.9 ± 1.0	12.5	21 ± 6.8
**62**	nf	25	90 ± 21
**37**	14 ± 5.0	25	59 ± 5
**116**	17 ± 3.5	100	58 ± 14
**120**	nf	12.5	nd
**56**	53 ± 2.3	50	91 ± 11
**55**	83 ± 5.6	50	105 ± 34
**4**	151	100	156 ± 45
**118**	nf	25	23 ± 5.5
**122**	nf	12.5	nd

Table S2: Comparison of affinity trends using three complementary methods.

Inhibition of haemagglutination was performed to bridge lacking information on the affinity from SPR and/or ITC. In the HAI assay, DMSO was added at a 10% concentration to maintain these most hydrophobic compounds in solution. nd: not determined. nf: not fittable.

Molecular dynamics simulations

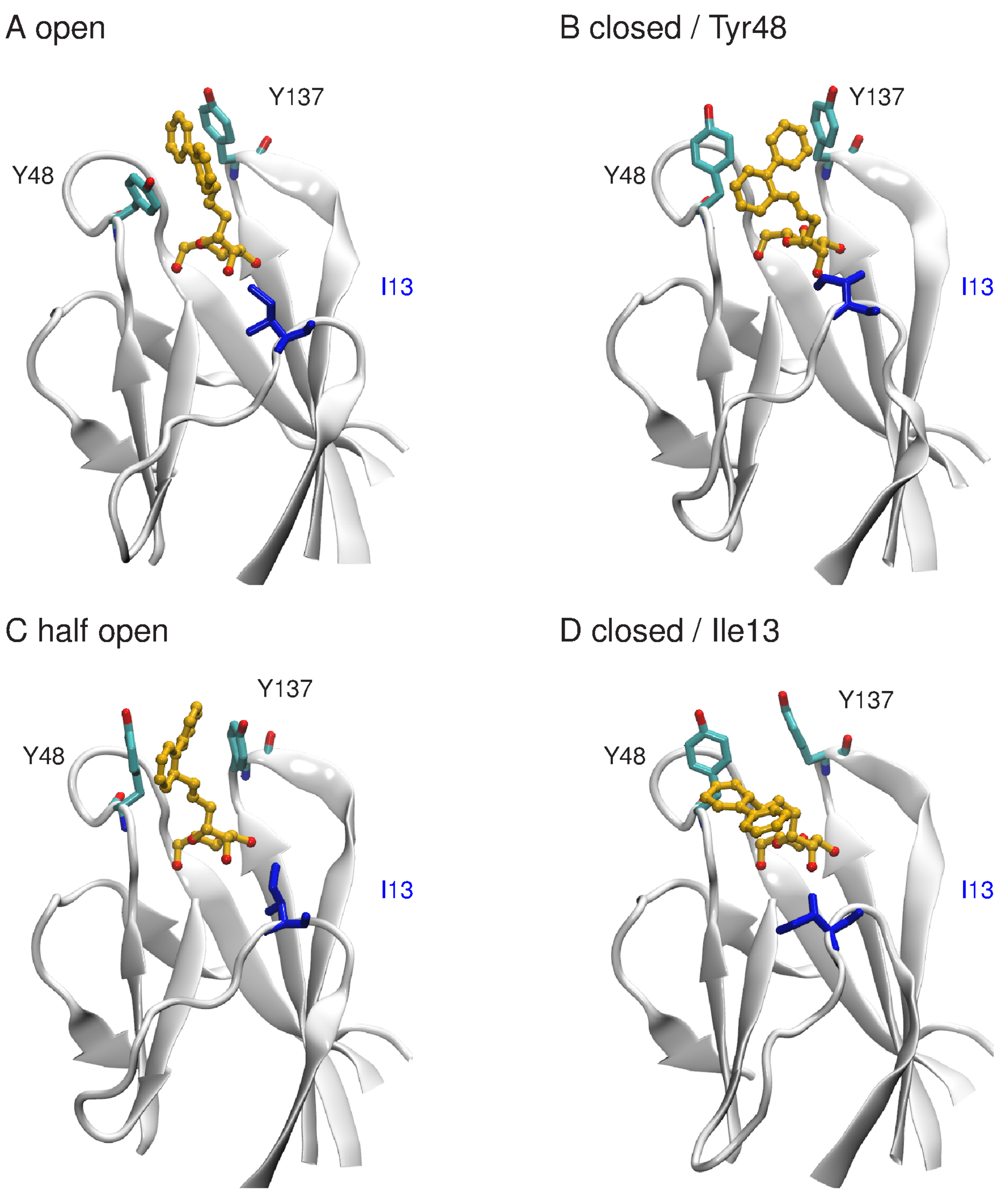

Figure S21: Four binding positions have been observed for the aglycon of 117 bound to FimH during molecular dynamics simulations. (1) the *open* conformer, in which the tyrosine gate is *open* and the second aromatic ring of **117** is almost parallel π-π stacked between Tyr48 and Tyr137; (2) the closed/*Tyr48* conformer, in which the tyrosine gate is *closed* with the first aromatic ring of **117** in a nearly parallel aromatic stacking interaction with Tyr48 and the second ring in T-stacking with Tyr137, (3) the *half-open* conformer, in which the aglycon part of **117** inserts partially between the two tyrosines; and (4) the *closed/Ile13* conformer, in which the first ring of the aglycon of **117** has stabilised the closing of the gate and the aglycon is oriented towards the Ile13 residue that resides in the clamp loop of the FimH

adhesin [3]. Upon visual inspection the poses in the lower row are less populated as the ones in the upper row. In all images, the protein is depicted as a white cartoon. The **117** ligand (orange) and important protein residues are shown in ball-and-stick representation. Ile13 in the clamp loop of FimH is colored blue.

Movie S1: The movement of **117** in the simulation after removal of the structural water. The protein is shown in white cartoon, the clamp loop region in lime, and important residues are shown as sticks: the tyrosine gate residues Tyr48, Ile52 and Tyr137 are in purple and the clamp loop residue Ile13 is shown in lime. Additionally, the oxygens of all waters within 3 Å of the sugar oxygens of **117** and the protein are shown as small reds spheres.

References

1. Touaibia, M.; Wellens, A.; Shiao, T.C.; Wang, Q.; Sirois, S.; Bouckaert, J.; Roy, R. Mannosylated g(0) dendrimers with nanomolar affinities to escherichia coli fimh. *ChemMedChem.* **2007**, *2*, 1190-1201.
2. Roy, R.; Dominique, R.; Das, S.K. Extended alkenyl glycosides by ruthenium-catalyzed cross-metathesis reaction and application toward novel c-linked pseudodisaccharides. *J Org Chem* **1999**, *64*, 5408-5412.
3. Le Trong, I.; Aprikian, P.; Kidd, B.A.; Forero-Shelton, M.; Tchesnokova, V.; Rajagopal, P.; Rodriguez, V.; Interlandi, G.; Klevit, R.; Vogel, V.*, et al.* Structural basis for mechanical force regulation of the adhesin fimh via finger trap-like beta sheet twisting. *Cell* **2010**, *141*, 645-655.
